# Functional characterization and allelic mining of *OsGLR* genes for potential uses in rice improvement

**DOI:** 10.3389/fpls.2023.1236251

**Published:** 2023-08-11

**Authors:** Wei Zeng, Hua Li, Fanlin Zhang, Xinchen Wang, Shamsur Rehman, Shiji Huang, Chenyang Zhang, Fengcai Wu, Jianfeng Li, Yamei Lv, Chaopu Zhang, Min Li, Zhikang Li, Yingyao Shi

**Affiliations:** ^1^ School of Agronomy, Anhui Agricultural University, Hefei, China; ^2^ Peking University Institute of Advanced Agricultural Sciences, Shandong Laboratory of Advanced Agriculture Sciences in Weifang, Weifang, China; ^3^ Institute of Crop Sciences, Chinese Academy of Agricultural Sciences, Beijing, China

**Keywords:** glutamate-like receptor (GLR) genes, gene-CDS-haplotype (gcHap) diversity, functional alleles, knockout mutants, rice improvement

## Abstract

Glutamate-like receptor (GLR) genes are a group of regulatory genes involved in many physiological processes of plants. With 26 members in the rice genome, the functionalities of most rice GLR genes remain unknown. To facilitate their potential uses in rice improvement, an integrated strategy involving CRISPR-Cas9 mediated knockouts, deep mining and analyses of transcriptomic responses to different abiotic stresses/hormone treatments and gene CDS haplotype (gcHap) diversity in 3,010 rice genomes was taken to understand the functionalities of the 26 rice GLR genes, which led us to two conclusions. First, the expansion of rice GLR genes into a large gene family during evolution had gone through repeated gene duplication events occurred primarily in two large GLR gene clusters on rice chromosomes 9 and 6, which was accompanied with considerable functional differentiation. Secondly, except for two extremely conserved ones (*OsGLR6.2* and *OsGLR6.3*), rich gcHap diversity exists at the remaining GLR genes which played important roles in rice population differentiation and rice improvement, evidenced by their very strong sub-specific and population differentiation, by their differentiated responses to day-length and different abiotic stresses, by the large phenotypic effects of five GLR gene knockout mutants on rice yield traits, by the significant association of major gcHaps at most GLR loci with yield traits, and by the strong genetic bottleneck effects and artificial selection on the gcHap diversity in populations *Xian* (*indica*) and *Geng* (*japonica*) during modern breeding. Our results suggest the potential values of the natural variation at most rice GLR loci for improving the productivity and tolerances to abiotic stresses. Additional efforts are needed to determine the phenotypic effects of major gcHaps at these GLR loci in order to identify ‘favorable’ alleles at specific GLR loci specific target traits in specific environments to facilitate their application to rice improvement in future.

## Introduction

Glutamate receptors (GLRs) are an important gene family that are known to play important roles in a variety of physiological processes of eukaryotes. In animals, GLRs are known to act as neurotransmitters that allow animals to respond external stimuli through ion channels (Lam et al., 1998). In plants, GLRs are known to play a role in a variety of physiological processes, including growth and development (Kang and Turano, 2003; Li et al., 2006; Michard et al., 2011; Vincill et al., 2013; Kong et al., 2015; Singh et al., 2016; Wudick et al., 2018), stress responses (Lu et al., 2014; Cheng et al., 2016; Cheng et al., 2018; Li et al., 2019; Philippe et al., 2019; Wang et al., 2019; Zheng et al., 2021), and pathogen defense (Mousavi et al., 2013; Shao et al., 2020; Liu et al., 2021; Xue et al., 2022; Yu et al., 2022). However, since GLR genes represent a large gene family that encodes membrane proteins embedded within the cell membrane, studying their functionalities in plants through traditional biochemical methods has been proven difficult.

In recent years, there has been growing interest to understand the molecular structure, expression patterns and functional roles of GLR genes in plants. Specially, technical advances in structural biology have enabled researchers to gain insights into the molecular structure of plant GLRs. One breakthrough study in this regard was the high-resolution structure of a glutamate receptor-like *AtGLR3.4* resolved from the model plant *Arabidopsis thaliana* using a cryo-electron microscopy (cryo-EM) in which the tetrameric assembly of *AtGLR3.4* subunits was revealed in a three-layer domain architecture that implies how GLRs transmit signals in plants (Green et al., 2021). In parallel, the expression patterns of plant GLR genes in response to different environmental cues such as abiotic stresses, pathogen infections and developmental transitions have recently been investigated. GLR genes have been shown to be essential for proper root growth and branching in *Arabidopsis thaliana* and rice, while plants with reduced GLR activity exhibit abnormal root growth (Li et al., 2006; Vincill et al., 2013; Singh et al., 2016). Plant GLR genes are also involved in plant responses to biotic and abiotic stresses. In particular, they have been shown to be vital for plant responses to salt, drought and chilling (cold) stresses (Lu et al., 2014; Cheng et al., 2016; Cheng et al., 2018; Zheng et al., 2021). In addition to their roles in abiotic stress tolerances, GLR genes also play an important role in plant defense systems against their pathogens. Cotton lines with CRISPR/Cas9-mediated *GhGLR4.8* knockout become highly susceptible to the fungal pathogen, *Fusarium oxysporum* f. sp. *vasinfectum* (*Fov*) and lost the ability to induce the calcium influx in response to the secreted proteins of *Fov* (Liu et al., 2021). *AtGLR3.3* and *AtGLR3.6* were shown to trigger leaf-to-leaf systemic signaling and induce systemic defenses against its piercing-sucking insect, *myzus persicae* (green peach aphid) or its chewing insect, *spodoptera litura* (cotton leafworm) when under insect attack (Xue et al., 2022). These findings highlight the importance of GLR genes in sensing and responding to pest and disease erosions and suggest their potential uses in developing new strategies for enhancing plant resistance to pests and diseases. While the importance of GLR genes in plant growth/development, stress responses and pathogen defense are becoming increasingly clear, how different GLR genes are involved in these complex processes remains unclear. Thus, it remains an interesting subject regarding how these GLR receptors can be used as targets for improving crop production and sustainability.

As the most important cereal crop grown worldwide, rice is also known to be the model species of cereals for functional and population genomic research. Today, more than 4,600 rice genes have been cloned and functionally characterized at the molecular level (https://funricegenes.github.io/). Meanwhile, the 3010 diverse rice accessions (3KRG) from 89 countries worldwide have been sequenced, revealing extremely rich genomic diversity in the primary gene pool of this important species (Project, 2014; Wang et al., 2018; Zhang et al., 2021). Unfortunately, the rapid progresses in rice functional and population genomic research have not yet been widely applied to development of more efficient breeding technologies. This is because, three pieces of information are essential to most plant breeders, but generally lacking for virtually all cloned rice genes. First, the current information on phenotypic effect(s) of a cloned gene is incomplete because this information was obtained from typical molecular biology experiments using near isogenic lines in specific genetic backgrounds and environments and thus is irrelevant to target environments of most breeding programs. In other words, the genotype by environment interaction and genetic background effects on important agronomic traits are largely uncharacterized for most cloned rice genes. Secondly, although there is rich natural allelic variation at most gene loci in rice populations (Zhang et al., 2021), but it remains a huge challenge regarding how to determine and mine desirable allele(s) at the cloned rice gene loci from rice germplasm accessions for improving specific target trait(s). Third, the functionalities of most rice genes, such as most GLR genes, remain unknown. Thus, given the fact that the functionalities of most rice genes remain unknown, one important challenge is to obtain the required information on these uncharacterized genes for breeding application without going through the typical time-consuming gene cloning processes.

In this study, we employed an integrated approach that involved phylogenetic analysis of rice GLR genes alongside their counterparts in closely related plants, phenotypic and functional characterization of important rice GLR genes using CRISPR technology and transcriptomic analyses, as well as comprehensive population genetic analyses of their gene-CDS-haplotype (gcHap) diversity in rice populations. Collectively, we demonstrated a more efficient strategy to generate important information for large numbers of gene loci required for developing novel breeding technologies in future.

## Materials and methods

### The phylogenetic relations between plant GLRs and mammals iGluRs (ionotropic glutamate receptors)

To understand the relationships between the rice GLR genes and those in animals and other plants, we compared all GLR genes in the Os-Nipponbare-Reference-IRGSP-1.0 reference genome with those in other flowering plants (Simon et al., 2023), including *Arabidopsis thaliana* (*At*), *Zea mays* (*Zm*), *Solanum lycopersicum* (*Sl*), *Gossypium hirsutum* (*Gh*), and *Raphanus sativus* (*Rs*), as well as basal land plants such as the moss *Physcomitrium patens* (*Pp*), the liverwort *Marchantia polymorpha* (*Mp*), and the lycophyte *Selaginella moellendorffii* (*Sm*), compared to the invertebrate *Caenorhabditis elegans* (*Ce*) and α-amino-3-hydroxy-5-methyl-4-isoxazolepropionic acid receptors (AMPARs), N-methyl-d-aspartate receptors (NMDARs), kainate receptors (KARs), and δ-receptors from mammals. Also included is the bacterial GluR0 from *Synechocystis PCC 6803* and AvGluR1 from the freshwater rotifer *Adineta vaga* (*Av*). Sequences are available in the **Supplementary Data 1**. Phylogenetic analysis of all plant GLRs and mammals iGluRs was conducted using the MEGA7.0 software (Shi et al., 2022). Muscle sequence comparison, which is a logarithmic expected multi-sequence comparison method, was performed to compare the protein sequences of GLR proteins of the relevant species. Then, a Bootstrap method with 1,000 replicates using the neighbor-joining (NJ) method (with p-distance correction) was used to assess the robustness of all nodes in the tree. The resulting phylogenetic tree was visualized using the website iTOL (https://itol.embl.de/), which allowed for the interactive exploration and customization of phylogenetic trees.

### The gene-CDS-haplotype (gcHap) diversity analyses

The construction method of gcHap dataset in the 3,010 rice genomes (3KRG, Wang et al., 2018) was referred to the *Molecular Plant* article of Zhang (Zhang et al., 2021). A total of 913,360 single nucleotide polymorphisms (SNPs) were identified within the coding sequence (CDS) regions of 42,497 of the 45,963 annotated genes in the Os-Nipponbare-Reference-IRGSP-1.0 reference genome. In the rice genome, no SNPs were found within the CDS regions of 3,466 genes, which were defined as house-keeping (HK) genes. Shannon’s equitability (*E_H_*) was used to evaluate the level of gcHap diversity at all rice GLR loci in specific rice populations or the whole species (Sheldon, 1969). Nei’s genetic identity ((*I_Nei_*) was used to measure the genetic similarity between two populations or individuals based on their gcHap frequencies at different GLR loci (Nei, 1972). The formula for calculating Shannon’s equability (*E_H_*) and Nei’s genetic identity (*I_Nei_*) were referred to the method used in this article by Zhang (Zhang et al., 2021). The GraphPad Prism 9 software was used for statistical analysis and picture plotting of the obtained data.

### The gcHaps diversity of rice GLR genes in modern varieties (MVs) and landraces (LANs)

To understand how modern breeding during past decades has affected gcHap diversity of rice GLR genes, detailed information was gathered for the total of 3,010 rice accessions of 3KRG. Of these, 732 were identified as *Xian* (*indica*) landraces (LANs-*Xian*), 358 were identified as *Geng* (*japonica*) landraces (LANs-*Geng*), 328 were identified as modern *Xian* varieties (MVs-*Xian*), and 139 were identified as modern *Geng* varieties (MVs-*Geng*). The first step was to download the predominant gcHaps (with the highest frequency in 3KRG) of each GLR gene from the RFGB webpage (https://www.rmbreeding.cn/Index). The frequency shift of the predominant gcHap for each GLR gene between modern varieties (MVs) and landraces (LANs) was calculated based on an R script (available at website https://github.com/isaac-Tsang/gcHap_diversity_LANs_MVs-). Then, A comparison was made between the distribution of gcHaps in modern *Xian* and *Geng* varieties and their respective landraces. At the same time, the loss and emergence of gcHap diversity were also analyzed in modern *Xian*/*Geng* varieties. Finally, the above data was plotted using GraphPad Prism 9 software.

### Determination of phenotypic effects of the major gcHaps of rice GLR genes

Firstly, we compiled a large-scale phenotypic data for 15 agronomic traits across 3010 Asia cultivated rice accessions. The study examined 15 agronomic traits, including Days to Heading (DTH, day), Plant Height (PH, cm), Flag Leaf Length (FLL, cm), Flag Leaf Width (FLW, cm), Panicle Number (PN, count), Panicle Length (PL, cm), Culm Number (CN, count), Culm length (CL, cm), Grain Length (GL, mm), Grain Width (GW, mm), Grain Length/Width Ratio (GLWR, ratio), Thousand Grain Weight (TGW, g), Leaf Rolling Index (LRI, %), Seedling Height (SH, cm) and Ligule Length (LL, mm). Phenotypic data for 15 rice traits were downloaded from the RFGB website (https://www.rmbreeding.cn/Index/). Next, the major gcHaps (with frequency ≥1% in 3KRG) of all rice GLR genes were obtained using an R script (https://github.com/isaac-Tsang/haplotype_network). Finally, the associations of major gcHaps with these agronomic traits across the diverse 3,010 rice accessions were achieved using an R script (https://github.com/isaac-Tsang/functional-importance-). The significance was calculated using one-way analysis of variance, and Tukey’s multiple comparison method was utilized to compare the significance among major gcHaps. The layout of the image is done in the Adobe Illustrator software (https://www.adobe.com/cn/products/illustrator.html).

### Expression profiles analysis of rice GLR genes under normal and abiotic stress conditions

Firstly, we downloaded a rice expression database (RED), a repository of gene expression profiles for rice (Xia et al., 2017). The database includes rice gene expression data derived from RNA-Seq analysis of eight different tissues (aleurone, anthers, callus, leaves, panicles, pistils, roots, seeds and shoot) at different growth stages, as well as under a variety of biotic and abiotic treatments, including ABA, JA, Cd, drought, and cold treatment conditions. The expression profiles of all GLR genes are available in the **Supplementary Data 2**. The expression patterns of all rice GLR genes from the dataset were analyzed using the heatmap.2 function of the R language and the layout of the image was done in the Adobe Illustrator software (https://www.adobe.com/cn/products/illustrator.html).

### Knockout and field phenotypic experiments of rice GLR genes

Based on their strong expression in more rice tissues and/or their strong responses to abiotic stresses, five rice *GLR* genes, *OsGLR2.2*, *OsGLR9.8*, *OsGLR6.8*, *OsGLR4.1* and *OsGLR7.1*, were chosen for functional analysis using the CRISPR-Cas9 technology in three steps. First, we designed two target sequences and corresponding detection primers for each of the five GLR genes (**Supplementary Data 3**). Cloning and transformation of these GLR genes were commissioned by Wuhan Boyuan Biotechnology Co., Ltd, using a *Geng* variety, Nipponbare as a background material for genetic transformation. Screening of the transformants *via* PCR amplification and sequencing to determine those carrying the desired mutations for the five target GLR genes were described previously (Zeng et al., 2020). 2×Spark Taq PCR Master Mix (with dye) (Shandong Sparkjade Biotechnology Co., Ltd.) is used for PCR amplification. The PCR products were sent to Sangon Biotech (Shanghai, China) for Sanger sequencing. DNASTAR Lasergene software was used for sequence analysis and alignment (https://www.dnastar.com/software/lasergene/).

Field phenotypic experiments of these GLR gene knockout mutants were conducted in the short-day environment of Hainan (18°15’ N and 109°30’ E) from Nov. 25 of 2021 – April 10 of 2022 and in the long-day environment of Hefei (31°52’ N and 117°17’ E) from May 27 – Oct. 7 of 2022. Each homozygous GLR knockout mutant was planted in a 3-row plot with 8 plants per row at a spacing of 15 cm between individual plants and 20 cm between rows, with three replications for each of the knockout mutant lines and Nipponbare as the wild type check in the field experiments. Four agronomic traits, 1000-grain weight (TGW), seed setting rate (SR), panicle number (PN), panicle length (PL), and plant height (PH) were measured on the five middle plants in each plot. Statistical analyses of the phenotypic data of the experiments were performed using the GraphPad Prism 9 software, while the layout of the obtained picture plotting data was done using the Adobe Illustrator software (https://www.adobe.com/cn/products/illustrator.html).

### Construction of gene CDS haplotype (gcHap) networks of rice GLR genes and association analyses of the major gcHaps at the GLR loci with yield traits in the 3KRG accessions

The construction of the gcHap networks containing major gcHaps with frequencies ≥1% in the 3KRG accessions or specific populations for each GLR gene was performed using the R package pegas (Paradis, 2010). The network of gcHaps for each GLR gene was produced using statistical parsimony algorithm, which is a method that connects the most closely related haplotypes first *via* the smallest number of mutations (Templeton et al., 1992). For more detailed steps, please refer to this *Molecular Plant* article of Zhang (Zhang et al., 2021). Finally, the layout of the image is done in the Adobe Illustrator software (https://www.adobe.com/cn/products/illustrator.html).

## Results

### The phylogenetic relations between plant GLRs and mammals iGluRs (ionotropic glutamate receptors)

**Figure 1:**
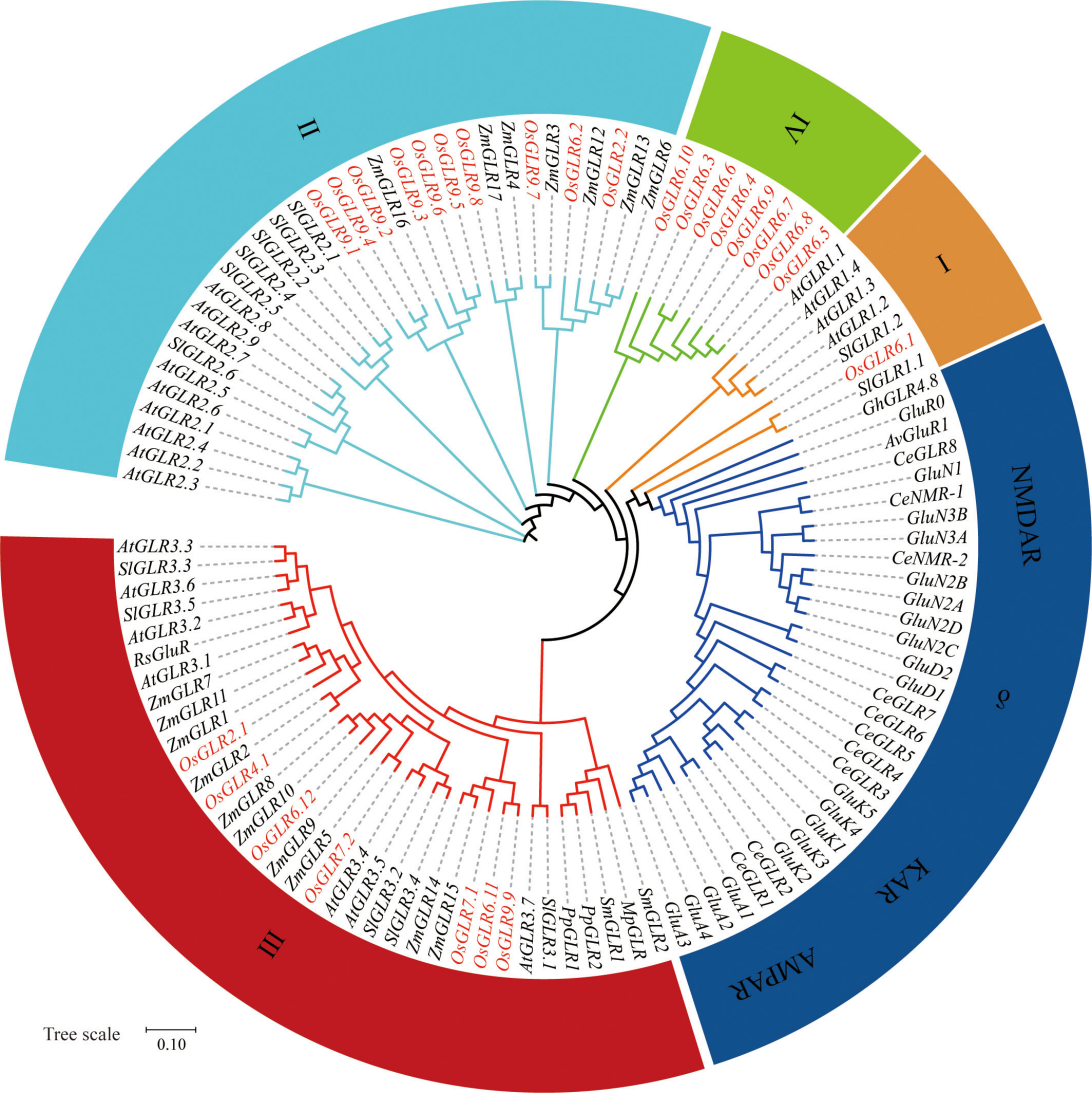
The phylogenetic relations between plant GLRs and mammals iGluRs (ionotropic glutamate receptors). The phylogenetic relations of glutamate receptors from *Oryza sativa Geng* (*Os*) based on the Os-Nipponbare-Reference-IRGSP-1.0 reference genome and other flowering plants that are described to show a conserved phenotype, including *Arabidopsis thaliana* (*At*), *Zea mays* (*Zm*), *Solanum lycopersicum* (*Sl*), *Gossypium hirsutum* (*Gh*), and *Raphanus sativus* (*Rs*), as well as basal land plants such as the moss *Physcomitrium patens* (*Pp*), the liverwort *Marchantia polymorpha* (*Mp*), and the lycophyte *Selaginella moellendorffii* (*Sm*), compared to the invertebrate *Caenorhabditis elegans* (*Ce*) and AMPARs, NMDARs, KARs, and δ-receptors from mammals (without prefix). Also included is the bacterial GluR0 from *Synechocystis PCC 6803* and AvGluR1 from the freshwater rotifer *Adineta vaga* (*Av*). *OsGLRs* are shown in colored text for clarity. Sequences were aligned using MUSCLE software, and the phylogenetic tree was constructed using the neighbor-joining method. Sequences are available in the **Supplementary Data 1**. AMPAR, α-amino-3-hydroxy-5-methyl-4-isoxazolepropionic acid receptor; KAR, kainate receptor; NMDAR, N-methyl-d-aspartate receptor; δ, δ-receptor.

We identified 26 GLR genes in the Os-Nipponbare-Reference-IRGSP-1.0 reference genome clustered primarily on chromosomes 2, 6, and 9. To understand the evolution and functional diversification of GLR genes, we performed a phylogenetic analysis to compare the rice GLR genes with their corresponding ones from various flowering plants, including *Arabidopsis thaliana* (*At*), *Zea mays* (*Zm*), *Solanum lycopersicum* (*Sl*), *Gossypium hirsutum* (*Gh*), and *Raphanus sativus* (*Rs*), three basal land plants, moss *Physcomitrium patens* (*Pp*), liverwort *Marchantia polymorpha* (*Mp*), and lycophyte *Selaginella moellendorffii* (*Sm*), one invertebrate *Caenorhabditis elegans* (*Ce*) and four mammals, AMPARs, NMDARs, KARs, and δ-receptors (**Figure 1**). Obviously, there is a clear distinction between GLRs in plants and animals, indicating a distant genetic relationship between the two groups. This is not unexpected as plants and animals represent distinct evolutionary lineages that diverged from a common ancestor more than a billion years ago. Consistent with our previous results (Shi et al., 2022), the 26 GLR genes could be roughly classified into four groups (**Figure 1**).

Group I contains a single *OsGLR* gene, *OsGLR6.1*, related to two *SlGLR* genes. Group II comprises 10 *OsGLR* genes in three subgroups: subgroup II-1 (*OsGLR9.1* – *OsGLR9.6*) related to maize *ZmGLR16*, subgroup II-2 (*OsGLR9.8*) closely related to maize *ZmGLR4* and *ZmGLR17*, and subgroup II-3 (*OsGLR9.7*, *OsGLR2.2* and *OsGLR6.2*) related to maize *ZmGLR3*, *ZmGLR6*, *ZmGLR12* and *ZmGLR13*. Group III has seven rice *OsGLR* genes in three subgroups: subgroup III-1 (*OsGLR6.11*, *OsGLR7.1* and *OsGLR9.9*) closely related to maize *ZmGLR14* and *ZmGLR15*, subgroup III-2 (*OsGLR6.12* and *OsGLR7.2*) related to maize *ZmGLR5*, *ZmGLR9*, *ZmGLR8* and *ZmGLR10*, and subgroup III-3 (*OsGLR4.1* and *OsGLR2.1*) related to maize *ZmGLR1*, *ZmGLR2*, *ZmGLR7* and *ZmGLR11*. Group IV is unique and has eight GLRs (*OsGLR6.4* – *OsGLR6.10*) found only in rice. The sequence differentiation of the 26 rice GLR genes suggested that the rice GLR genes had undergone unique evolutionary processes, including gene duplication events and functional differentiation that distinguished them from GLR genes in other species. The close relationships of most rice GLR genes with maize GLR genes suggest these rice GLRs might share similar functionalities with the maize ones, while the unique group IV GLRs clustered on rice chromosome 6 are the most interesting ones for future research focuses.

### Functional differentiation and tissue specificity of rice GLR genes

**Figure 2:**
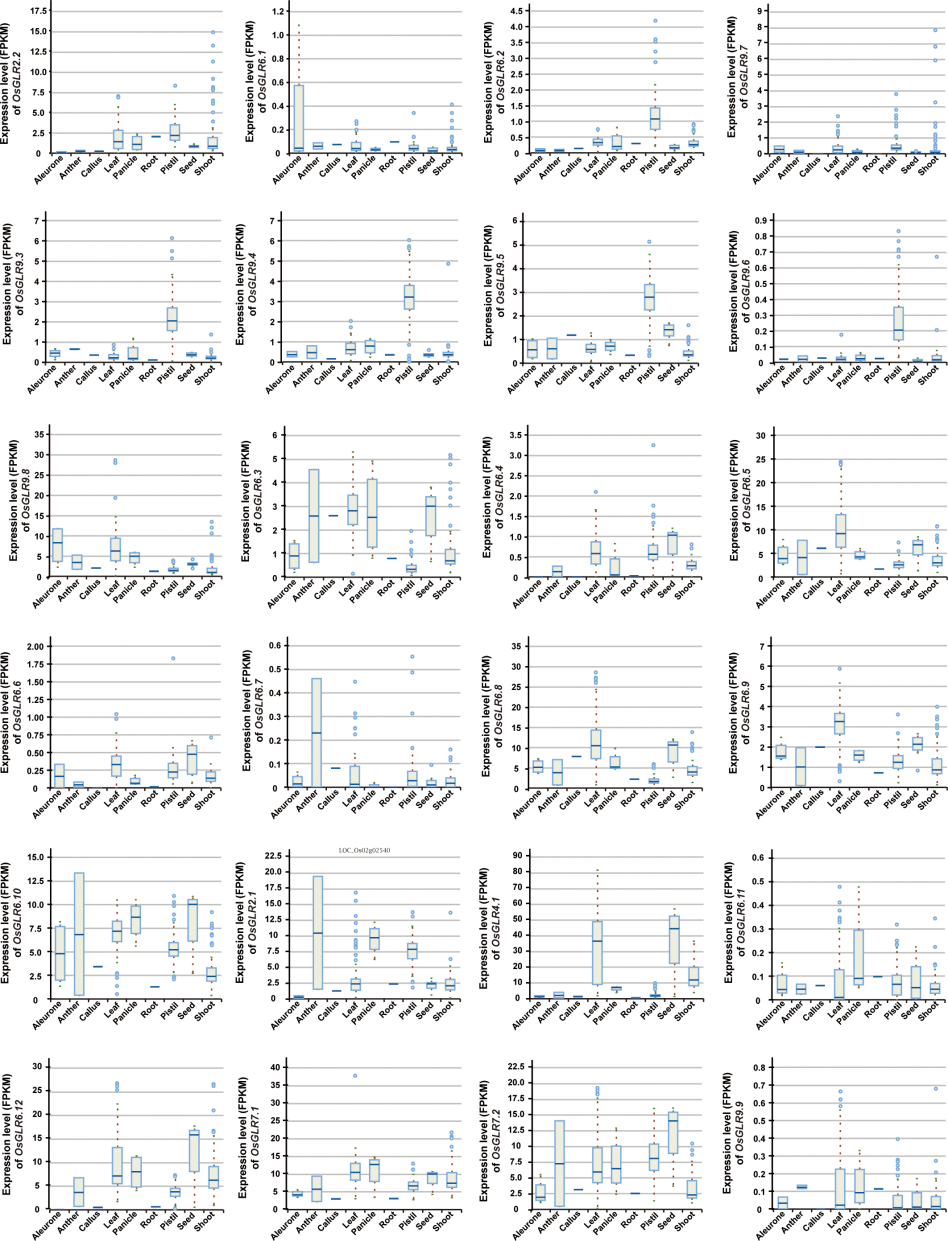
The expression level of the *OsGLR* genes in different rice tissues under the normal conditions. The plant tissues mainly include aleurone, anther, callus, leaf, panicle, pistil, root, seed and shoot.

To understand the functionalities of different rice GLR genes, we analyzed the expression levels of all GLR genes in eight different tissues of rice, including aleurone, anther, callus, leaf, panicle, pistil, root, seed and shoot tissues. Under the normal conditions, different GLR genes showed varied expression levels in different tissues of rice (**Figure 2**). Notably, *OsGLR2.1*, *OsGLR4.1*, *OsGLR6.5*, *OsGLR6.8*, *OsGLR6.10*, *OsGLR6.12*, *OsGLR7.1*, *OsGLR7.2*, and *OsGLR9.8* were each expressed strongly (mean FPKM > 5) in more than one tissue, indicating their importance in rice growth and development. Among these genes, *OsGLR4.1* had the highest expression levels in leaf and seed tissues (**Figure 2**). *OsGLR2.2*, *OsGLR6.3* and *OsGLR6.9* showed moderate levels of expression in one or more tissues, while the remaining ones (*OsGLR6.1*, *OsGLR6.2*, *OsGLR6.4*, *OsGLR6.6*, *OsGLR6.9*, *OsGLR6.11*, *OsGLR9.7* and *OsGLR9.9*) showed low levels of expression (<1) in all tissues. Interestingly, four GLRs (*OsGLR9.3 - OsGLR9.6*) showed a moderate expression specifically in the pistil tissue but extremely low expression in other tissues. These results indicated that one important aspect in the functional differentiation of rice GLR genes was reflected in their tissue specificity, though it remains a mystery regarding how the expression pattern of each rice GLR gene related its functions in its specifically expressed tissues.

### Expression profiles of rice GLR genes in response to abiotic stresses

**Figure 3:**
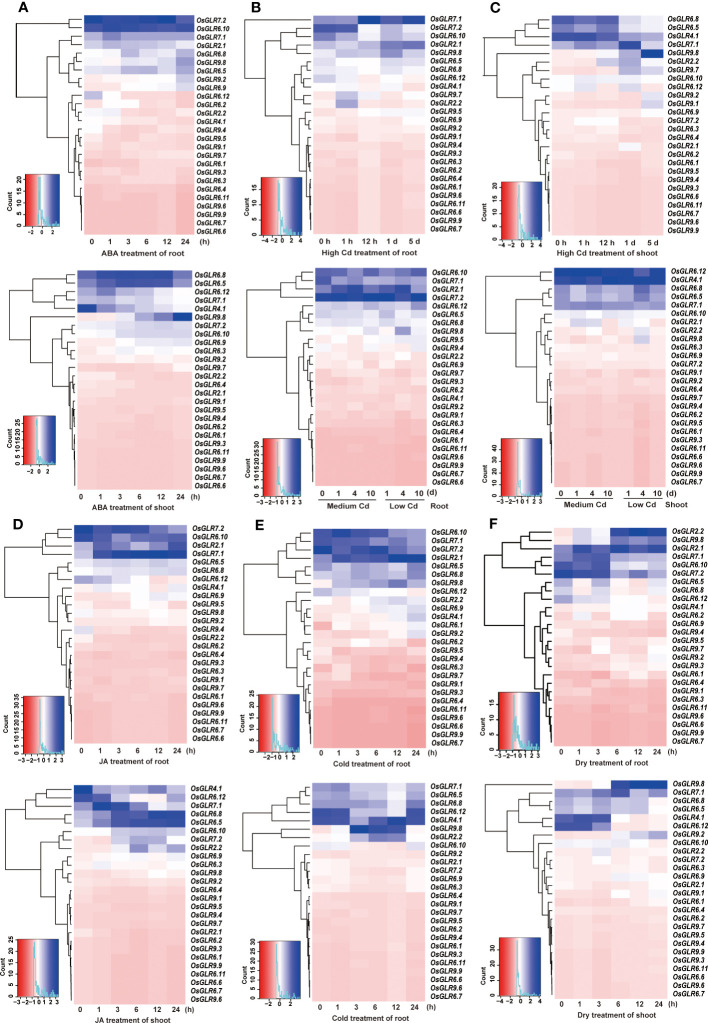
Integrative analysis of the rice *OsGLR* gene family expression profiles under ABA **(A)**, Cd **(B, C)**, JA **(D)**, cold **(E)**, and dry **(F)** treatment conditions.

In order to gain insights into the roles of rice GLR genes under different abiotic stress conditions, we conducted an integrative analysis of their expression profiles under abscisic acid (ABA), JA, cadmium (Cd), drought, and cold treatments. When treated with ABA, rice *OsGLR6.5*, *OsGLR6.8*, *OsGLR6.10, OsGLR7.2* and *OsGLR9.8* showed increased expression in both shoots and roots (**Figure 3A**), indicating that they might be involved in the ABA regulated pathways. Interestingly, *OsGLR4.1* and *OsGLR9.8* showed opposite expression patterns in shoot under the ABA treatment (**Figure 3A**), in which *OsGLR4.1* showed gradually decreased expression in response to the ABA treatment over time, whereas *OsGLR9.8* showed gradually increased expression over time (**Figure 3A**). Under three different Cd (cadmium) ion stress conditions, *OsGLR2.1*, *OsGLR4.1*, *OsGLR6.5, OsGLR6.8*, *OsGLR7.1*, *OsGLR7.2* and *OsGLR9.8* showed increased but varied expression (**Figures 3B, C**). Among them, *OsGLR4.1* and *OsGLR7.1* genes had the highest expression in both roots and shoots under different concentrations of Cd stress. Under the JA treatment, *OsGLR2.1*, *OsGLR7.1*, *OsGLR7.2*, *OsGLR6.5*, *OsGLR6.8* and *OsGLR6.10* showed significantly increased expression in either or both roots and shoots (**Figure 3D**), indicating they might be involved in the JA signaling pathways. Interestingly, *OsGLR4.1* showed a similarly decreasing expression in shoots under the JA treatment as it did in response to the ABA treatment. Under the cold stress, *OsGLR2.1*, *OsGLR2.2, OsGLR7.1*, *OsGLR7.2*, *OsGLR6.5*, *OsGLR6.10, OsGLR6.12*, and *OsGLR9.8*, showed significantly increased but varied expression in either or both tissues (**Figure 3E**). Similarly, the expression of *OsGLR2.1*, *OsGLR2.2, OsGLR7.1*, *OsGLR4.1*, *OsGLR6.12* and *OsGLR9.8* was significantly upregulated by the drought treatment (**Figure 3F**). Taking together, ten rice *GLR* genes showed significantly increased expression in response to the five external treatments. Of these, *OsGLR7.1*, *OsGLR2.1* and *OsGLR9.8* were the most noteworthy ones. The expression of *OsGLR7.1* in both roots and shoots and expression of *OsGLR2.1* in roots were strongly upregulated in response to JA, Cd, drought and cold, suggesting their important roles in the JA signaling pathways involved in abiotic stresses. In contrast, the expression of *OsGLR9.8* in shoots was strongly upregulated under the Cd, drought and cold stresses, indicating its important role in rice responses to abiotic stresses.

### Functional determination of five rice GLR genes by knockout and phenotypic assessment

**Figure 4:**
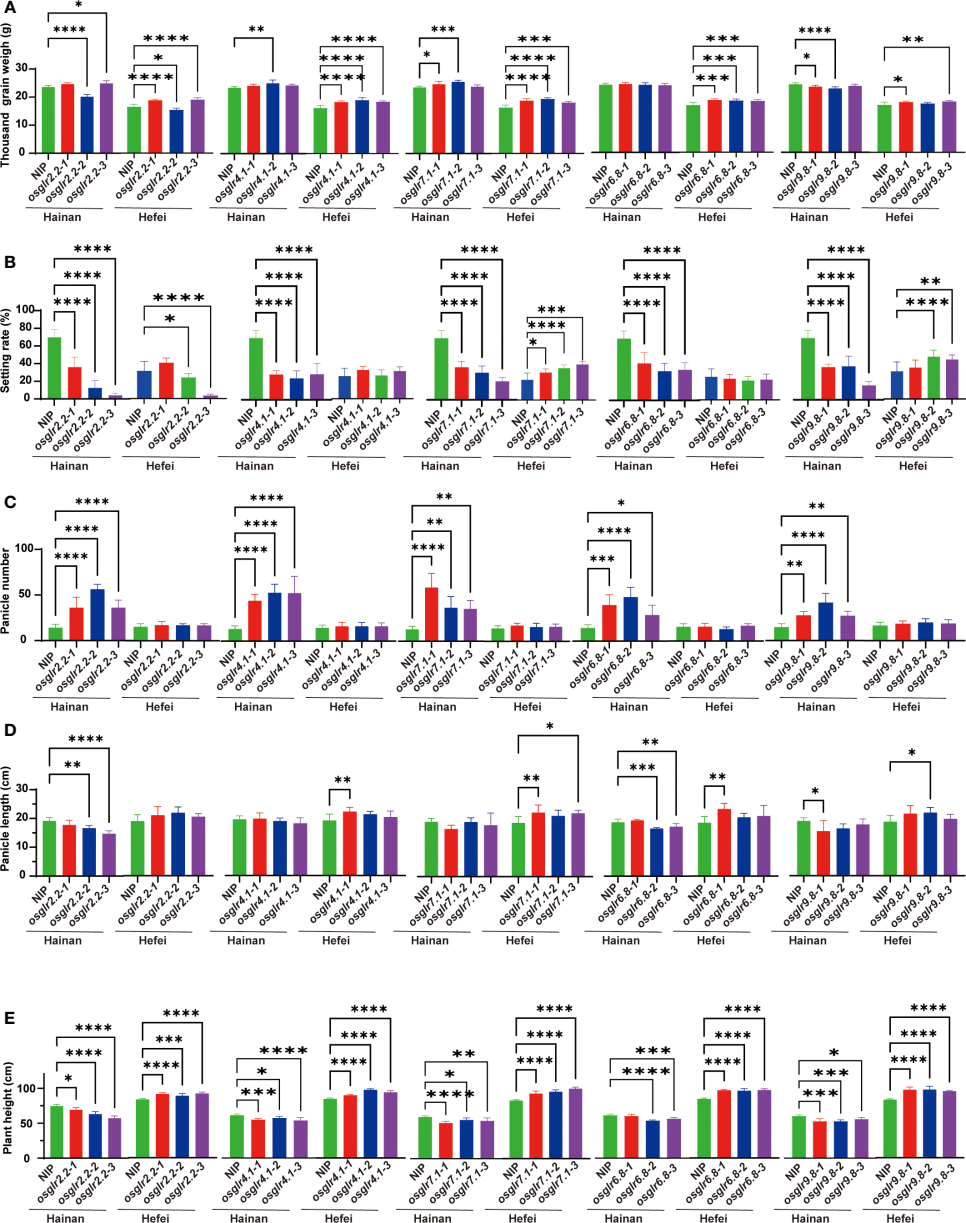
Phenotypic comparisons for five agronomic traits of knockout mutants of five rice *OsGLR* genes evaluated in two environments. **(A-E)** The agronomic traits represented by **(A-E)** are 1000-grain weight (g), seed setting rate (%), panicle number, panicle length (cm) and plant height (cm), respectively. *, **, *** and **** meant statistically significant at a level of 0.05, 0.01, 0.001 and 0.0001, respectively.

To assess the functional importance of rice GLR genes, five important *OsGLR* genes, *OsGLR2.2*, *OsGLR9.8*, *OsGLR6.8*, *OsGLR4.1* and *OsGLR7.1* were selected based on previous transcriptomic analyses for further gene knockout experiments using the CRISPR-Cas9 technology. Specifically, three independent homozygous mutants carrying different mutation sites for each of the GLR genes were obtained (**Supplementary Figure 1**) and assessed for 1000-grain weight (TGW), seed setting rate (SR), panicle number (PN), panicle length (PL) and plant height (PH) under the short-day and long-day environments of Hainan and Hefei. Three interesting observations were noted regarding the phenotypic differences for the measured traits between the GLR knockout mutants and the wild type (**Figure 4**). First, significant differences were observed between the knockout mutants of each GLR gene and the wild type for all the measured traits, but these differences varied greatly depending on the environments where these materials were assessed (**Figures 4A–E**). In other words, all five GLR genes showed huge genotype x environment interactions for their phenotypic effects on the measured traits, particularly for SR, PN and PH. When compared with the wild type, the mutant lines of all five GLR genes showed greatly reduced SR under the short-day environment of Hainan, but their SR was either significantly increased (*OsGLR7.1* and *OsGLR9.8*), unchanged (*OsGLR4.1* and *OsGLR6.8*) or varied (*OsGLR2.2*) in the long-day environment of Hefei (**Figure 4B**). The mutant lines of all five GLR genes showed greatly increased PN under the short-day environment of Hainan, but not in the long-day environment of Hefei (**Figure 4C**). More interestingly, the mutant lines of all five GLR genes showed significantly reduced PH under the short-day environment of Hainan, but significantly increased PH in the long-day environment of Hefei (**Figure 4E**). Secondly, one or more mutant lines of the GLR genes, except for *OsGLR6.8* in Hainan, showed significant increased TGW in both environments (**Figure 4A**). Thirdly, the phenotypic differences between the mutant lines and wild type varied among different mutant lines of the same GLR genes, particularly for PL (**Figure 4D**), indicating that mutation sites in different GLR genes did make differences in generating phenotypically detectable mutants. These results led us to an important conclusion, i.e. obvious functional differentiation and functional redundancy regarding their phenotypic effects on the measured traits, the five rice GLR genes appeared to play an important role in contributing to the significant G x E interaction of agronomic traits in rice across the short-day and long-day environments.

### The rich genetic and allelic diversity at rice GLR loci in rice populations

**Figure 5:**
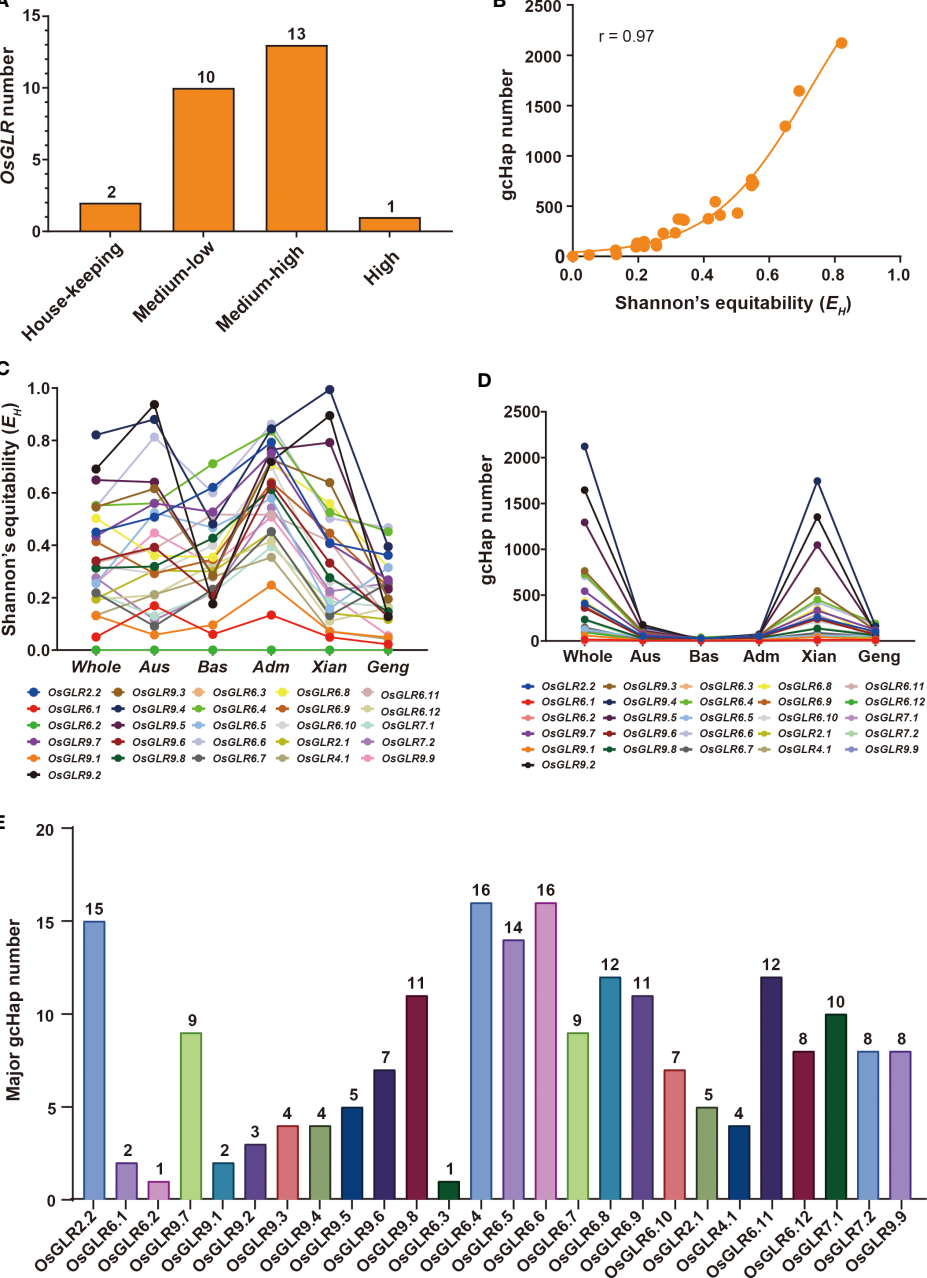
Population diversity of *OsGLR* genes in 3010 Asia cultivated rice accessions. **(A)** *OsGLR* genes distribution of five diversity gene types (house-keeping (HK) genes without SNP variants, low-diversity genes (0 < *E_H_* < 0.05, *E_H_* = 0.020 ± 0.015, and gcHapN = 12 ± 10), medium- to low-diversity genes (0.05 ≤ *E_H_* < 0.3, *E_H_* = 0.171 ± 0.065, and gcHapN = 82 ± 67), medium- to high-diversity genes (0.3 ≤ *E_H_* < 0.7, *E_H_* = 0.444 ± 0.110, and gcHapN = 498 ± 305) and a high-diversity gene (0.7 ≤ *E_H_* ≤ 1, *E_H_* = 0.804 ± 0.075, and gcHapN = 1767 ± 458), Shannon’s equitability (*E_H_*) takes into account both the number of species present (species richness) and the relative abundance of each species within the community. It ranges from 0 to 1, with values closer to 1 indicating a more even distribution of species abundance, and values closer to 0 indicating a less even distribution. **(B)** The relationship between Shannon’s equitability (*E_H_*) and gcHapN of *OsGLR* genes. **(C)** *E_H_* values of *OsGLR* genes in different population organization. **(D)** gcHap number (gcHapN) of *OsGLR* genes in different population organization. **(E)** The major gcHap (≥ 30 rice accessions) number of *OsGLR* genes.

Given the functional importance of the rice GLR genes, it is important to understand their genetic diversity in rice populations in order to facilitate their applications in rice improvement. Using the gene CDS haplotype (gcHap) data from the 3,010 rice genome project (3KRG), we obtained the Shannon’s equitability, *E_H_*, and number of gcHaps and major gcHaps (with frequency ≥1% in 3KRG) of the 26 GLR genes in four major rice populations (**Supplementary Data 4**, **Figure 5**). Of the 26 GLR genes, *OsGLR6.2* and *OsGLR6.3* are extremely conserved house-keeping (HK) genes with *E_H_* = 0, and gcHapN = 1 (**Figure 5A**), indicating all rice varieties share the same alleles at the two loci. For the remaining 24 polymorphic GLR genes, the average numbers of gcHaps (major gcHaps) and *E_H_* were 476.4 (8.4) and 0.368 in the 3KRG accessions. However, different GLR genes vary considerably in their genetic diversity. *OsGLR9.4* has the highest genetic diversity with *E_H_* of 0.821 and 2,182 gcHaps (**Figures 5C, D**), followed by *OsGLR9.2* with *E_H_* of 0.691 and 1,681 gcHaps, and *OsGLR9.5* with *E_H_* of 0.649 and 1,374 gcHaps. *OsGLR6.1* has the lowest diversity with *E_H_* of 0.050 and six gcHaps (**Supplementary Data 4**).

Different rice populations also vary considerably in their diversity of the GLR genes. The mean *E_H_* of the 24 GLR genes were 0.367 in population *Xian*, 0.213 in population *Geng*, 0.409 in population *Aus*, and 0.352 in population *Bas*. The numbers of detected gcHaps and major gcHaps were 342.1 and 7.7 in population *Xian*, 76.2 and 4.8 in population *Geng*, 47.8 and 5.2 in population *Aus*, and 17.8 and 5.0 in population *Bas* (**Supplementary Data 4**). Clearly, the numbers of gaHaps and major gcHaps in different rice populations were largely determined by their population sizes, implying that most gcHaps are rare ones with very low frequencies in different rice populations (**Figures 5C, D**).

**Figure 6:**
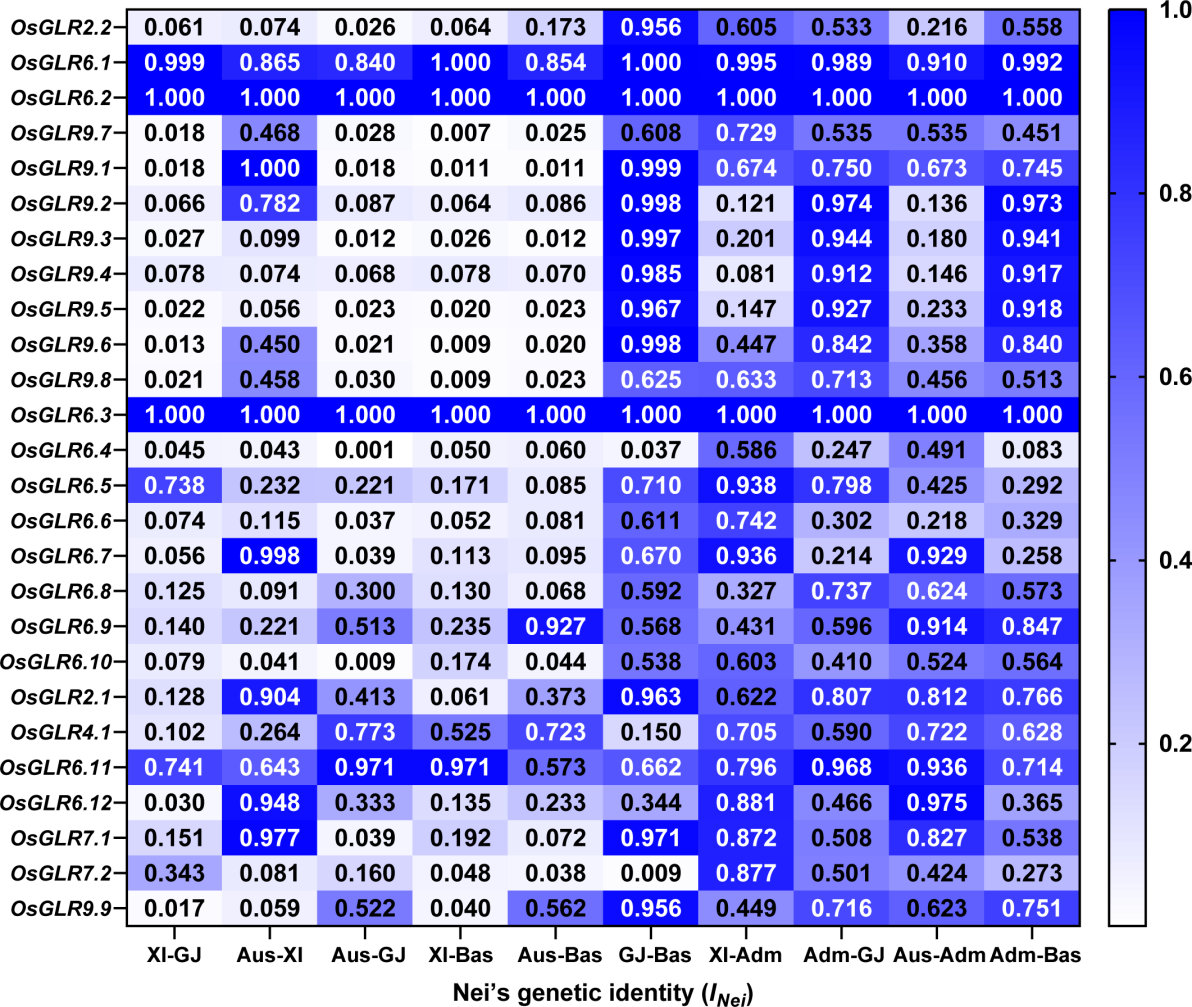
The Nei’s genetic identity (*I_Nei_*) of rice *OsGLR* genes between all pairwise populations calculated from the gcHap data.

To understand how different GLR genes were differentiated among major rice populations, we estimated the Nei’s genetic identity (*I_Nei_*) of all GLR genes using the gcHap data of the 24 polymorphic *OsGLR* genes, except for *OsGLR6.2*, *OsGLR6.3*, between all pairwise populations. Except for *OsGLR6.1*, *OsGLR6.5* and *OsGLR6.11*, strong *Xian*-*Geng* sub-specific differentiation (*I_Nei_* <0.35) was observed at 21 of the GLR genes, 20, 17 and 18 of which also showed strong *Xian*-*Bas*, *Aus*-*Geng* and *Aus*-*Bas* differentiation (**Figure 6**, **Supplementary Data 5**), indicating that the allelic differentiation at most GLR loci had contributed very strongly to the differentiation of major rice populations and expectedly to the adaptations of different rice populations to their environments.

### Impact of modern breeding on gcHap diversity of rice GLR genes

**Figure 7:**
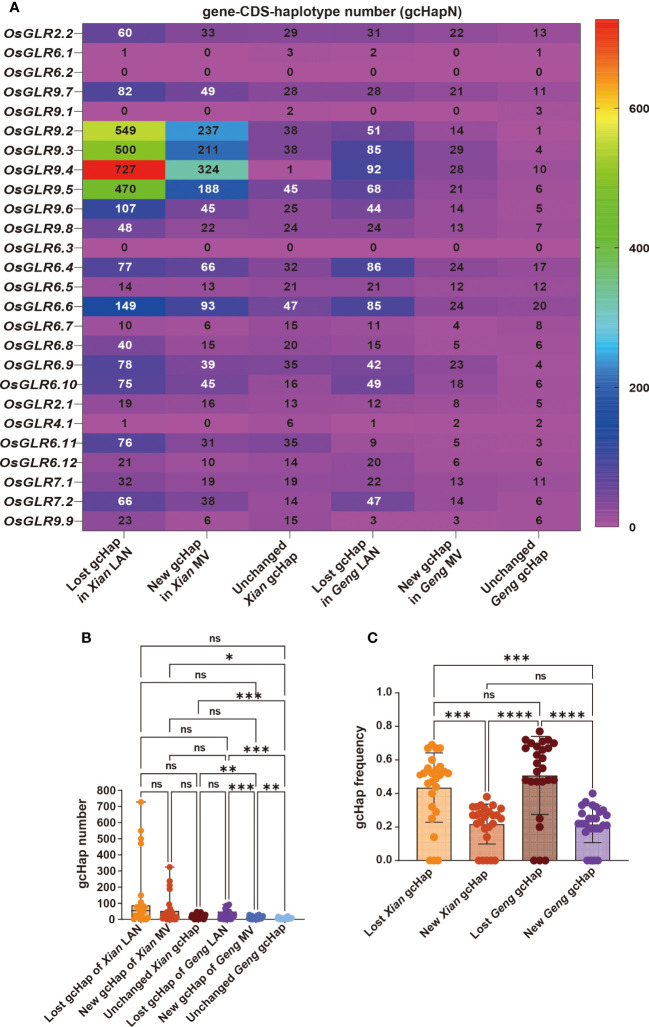
The lost, new and unchanged gcHap variation of rice 26 *OsGLR* genes during modern breeding. **(A)** Heatmap of the numbers of lost, newly emerged, and unchanged gcHaps. **(B)** The comparative analysis of the numbers of lost, newly emerged, and unchanged gcHaps. **(C)** The frequency comparison of lost, newly emerged, and unchanged gcHaps. The Tukey’s multiple comparison method was used. ****P < 0.0001, ***P < 0.001, **P < 0.01, *P < 0.05, and n.s., not significant.

To understand how modern breeding during past decades has affected gcHap diversity of rice GLR genes, we analyzed the diversity parameters of all the GLR genes in modern varieties (MVs) and landraces (LANs), including 732 *Xian* landraces (LANs-*Xian*), 358 *Geng* landraces (LANs-*Geng*), 328 modern *Xian* varieties (MVs-*Xian*), and 139 modern *Geng* varieties (MVs-*Geng*) (**Supplementary Data 6**). In population *Xian*, the average *E_H_* of the 24 GLR genes was 0.427 in MVs-*Xian*, a 13.3% increase compared to 0.377 in LANs-*Xian* (**Table 1**). Increased diversity in MVs-*Xian* was observed in eight GLR genes (*OsGLR6.4*, *OsGLR6.5*, *OsGLR6.6*, *OsGLR6.9*, *OsGLR6.10*, *OsGLR9.7*, *OsGLR7.1* and *OsGLR7.2*) out of the 24. No GLR loci showed reduced diversity. Interestingly, MVs-*Xian* had an average of 85 gcHaps/locus, significantly lower than the 156.7 gcHaps/locus in LANs-*Xian*. Detailed examination revealed that the MVs-*Xian* lost average 134.4 gcHaps/locus and 1.6 major gcHaps/locus that were present the LANs-*Xian*, but absent in MVs-*Xian*, which resulted apparently from the genetic bottlenecks during modern breeding (**Table 1**, **Figure 7A**).

**Table 1:** Comparisons in genetic diversity of the 26 GLR genes between the landraces and modern varieties.

Gene name	*Xian* (*indica*)							*Geng* (*japonica*)						
	LANs		MVs		Change of gcHapN (major gcHapN)			LANs		MVs		Change of gcHapN (major gcHapN)		
	*E_H_*	gcHapN (major gcHapN)	*E_H_*	gcHapN (major gcHapN)	Lost	New	Retained	*E_H_*	gcHapN (major gcHapN)	*E_H_*	gcHapN (major gcHapN)	Lost	New	Retained
***OsGLR2.1***	0.177	32 (4)	0.233	29 (5)	19 (2)	16 (1)	13 (3)	0.098	17 (2)	0.151	13 (4)	12 (3)	8 (1)	5 (1)
***OsGLR2.2***	**0.386**	**89 (13)**	**0.440**	**62 (12)**	**33 (1)**	**60 (2)**	**29 (11)**	**0.349**	**44 (7)**	**0.477**	**35 (8)**	**31(4)**	**22 (3)**	**13 (4)**
***OsGLR4.1***	**0.077**	**7 (2)**	**0.081**	**6 (3)**	1 (1)	0	6 (2)	**0.044**	**4 (4)**	**0.035**	**3 (2)**	1 (1)	2 (3)	2 (1)
***OsGLR6.1***	0.031	4 (2)	0.027	3 (3)	1 (1)	0	3 (2)	0.012	3 (1)	0.000	1 (1)		0	1(1)
***OsGLR6.4***	0.392	109 (12)	0.554	98 (13)	77 (3)	66 (2)	32 (10)	0.636	103 (19)	0.475	41 (11)	86 (4)	24 (12)	17 (7)
***OsGLR6.5***	0.255	35 (11)	0.389	34 (11)	14 (2)	13 (2)	21 (9)	0.368	33 (10)	0.395	24 (12)	21 (4)	12 (2)	12 (8)
***OsGLR6.6***	0.588	196 (13)	0.709	140 (15)	149 (5)	93 (3)	47 (10)	0.603	105 (13)	0.490	44 (10)	85 (2)	24 (5)	20 (8)
***OsGLR6.7***	0.115	25 (4)	0.164	21 (4)	10 (1)	6 (1)	15 (3)	0.263	19 (7)	0.264	12 (5)	11	4 (2)	8 (5)
***OsGLR6.8***	**0.312**	**60 (8)**	**0.367**	**35 (9)**	40 (2)	15 (1)	20 (7)	**0.305**	**21 (8)**	**0.270**	**11 (8)**	15 (4)	5 (4)	6 (4)
***OsGLR6.9***	0.423	113 (12)	0.507	74 (15)	78 (4)	39 (1)	35 (11)	0.268	46 (3)	0.314	27 (5)	42 (2)	23	4 (3)
***OsGLR6.10***	0.230	91 (6)	0.326	61 (5)	75 (1)	45 (2)	16 (4)	0.299	55 (3)	0.271	24 (4)	49 (2)	18 (1)	6 (2)
***OsGLR6.11***	0.502	112 (17)	0.474	66 (14)	76 (1)	31 (4)	35 (13)	0.109	12 (3)	0.150	8 (3)	9	5	3 (3)
***OsGLR6.12***	0.202	35 (5)	0.166	24 (5)	21 (1)	10 (1)	14 (4)	0.167	26 (2)	0.190	12 (8)	20 (6)	6	6 (2)
***OsGLR7.1***	**0.266**	**51 (7)**	**0.385**	**38 (10)**	32 (5)	19 (2)	19 (5)	**0.335**	**33 (8)**	**0.420**	**24 (8)**	22 (2)	13 (2)	11 (6)
***OsGLR7.2***	0.232	80 (5)	0.325	52 (7)	66 (2)	38	14 (5)	0.343	53 (4)	0.326	20 (6)	47 (2)	14	6 (4)
***OsGLR9.1***	0.019	2 (2)	0.037	2 (2)	0	0	2 (2)	0.012	3 (2)	0.042	3 (2)	0	0	3 (2)
***OsGLR9.2***	0.895	587 (2)	0.930	275 (3)	549 (2)	237 (1)	38 (1)	0.188	52 (4)	0.120	15 (1)	51	14 (3)	1 (1)
***OsGLR9.3***	0.862	538 (2)	0.873	249 (4)	500 (3)	211 (1)	38 (1)	0.324	89 (3)	0.271	33 (1)	85	29 (2)	4 (1)
***OsGLR9.4***	0.998	728 (0)	0.998	325 (0)	727	324	1	0.477	102 (6)	0.450	38 (6)	92 (1)	28 (1)	10 (5)
***OsGLR9.5***	0.862	515 (7)	0.872	233 (8)	470 (3)	188 (2)	45 (5)	0.337	74 (4)	0.275	27 (2)	68	21 (2)	6 (2)
***OsGLR9.6***	0.367	132 (5)	0.408	70 (7)	107 (3)	45 (1)	25 (4)	0.175	49 (1)	0.180	19 (5)	44 (4)	14	5 (1)
***OsGLR9.7***	0.347	110 (8)	0.468	77 (10)	82 (3)	49 (1)	28 (7)	0.243	39 (6)	0.366	32 (6)	28 (2)	21 (2)	11 (4)
***OsGLR9.8***	**0.300**	**72 (7)**	**0.337**	**46 (8)**	48 (2)	22 (1)	24 (6)	**0.166**	**31 (3)**	**0.235**	**20 (5)**	24 (2)	13	7 (3)
***OsGLR9.9***	0.205	38 (7)	0.186	21 (6)	23 (1)	6 (2)	15 (5)	0.056	9 (2)	0.115	9 (4)	3 (2)	3	6 (2)
**Mean**	**0.377**	**157 (6.7)**	**0.427**	**85 (7.5)**	134.4 (2.2)	62.8 (1.6)	22.3 (5.7)	**0.257**	**43 (5.2)**	**0.262**	**21 (5.3)**	35.3 (2.8)	13.5 (3.0)	7.2 (3.3)

E_H_ and gcHapN (major gcHapN) are the Shannon’s equitability, and number of gcHaps (≥ 1% varieties) identified. The bold GLR genes were those used in functional analyses by the CRISPR-Case9 knockout experiments.

Additionally, the proportion of newly emerged gcHaps observed in modern varieties is significantly lower than that of lost gcHaps (**Figures 7B, C**). MVs-*Xian* gained average 62.8 new gcHaps/locus and 2.2 new major gcHaps that were absent in the LANs-*Xian*. The new gcHaps were apparently generated by intra-genic recombination during breeding, while all new major gcHaps were rare ones in the LANs-*Xian* but became major ones with significantly increased frequencies in the MVs-*Xian* (**Table 1**). We noted four GLR loci (*OsGLR9.2 - OsGLR9.5*) in a large cluster on chromosome 9 (**Figure 7A**), each of which had a huge number of gcHaps and showed more dramatically changes in gcHapN, suggesting genetic drift and unequal crossing-over occurred more frequently among duplicated GLR genes in the GLR gene clusters. The increased diversity and reduced gcHapN observed at many of the GLR loci suggested that significant frequency shifts in major gcHaps had occurred at these GLR loci in population *Xian* during breeding. Indeed, significant shifts in frequencies of the predominant gcHaps, F_(P)_, were observed at 13 of the 24 GLR loci, including significantly reduced F_(P)_ at nine of the GLR loci and significantly increased F_(P)_ at four of the GLR loci (**Supplementary Data 7**). Notably, at *OsGLR7.1* locus, the predominant allele (Hap1) had an F_(P)_ of 0.60 and 0.34 in populations LANs-*Xian* and MVs-*Xian*, or a major reduction in F_(P)_ by 0.25 in MVs-*Xian* (**Supplementary Figure 3**), accompanied by significantly increased F_(P)_ of five newly major gcHaps, all of which were rare gcHaps with frequencies <0.01 in the LANs-*Xian* but became major gcHaps in the MVs-*Xian*. Similar observations were noted on four other loci (*OsGLR6.11*, *OsGLR9.1*, *OsGLR6.5* and *OsGLR6.4*) where significantly reduced F_(P)_ was accompanied by emergences of 1-2 new major gcHaps in the MVs-*Xian* from rare ones in the in the LANs-*Xian*. The remaining 11 loci showing no significant differences in F_(P)_ between LANs-*Xian* and MVs-*Xian* included the four GLR loci (*OsGLR9.2 - OsGLR9.5*) where no or few predominant gcHaps were present.

In population *Geng*, the average *E_H_* of the 24 GLR genes was similar in the MVs-*Geng* (0.262) to 0.257 of the LANs-*Geng* (**Supplementary Data 6**). However, significant reduced diversity in the MVs-*Geng* was observed at only two GLR loci (*OsGLR6.4* and *OsGLR6.6*), while significantly increased diversity was observed at *OsGLR2.2*, *OsGLR7.1* and *OsGLR9.7* genes. Similarly, the MVs-*Geng* had an average of 20.6 gcHaps/locus, significantly less than 42.6 gcHaps/locus in the LANs-*Geng* (**Figure 7**; **Table 1**). In fact, the MVs-*Geng* lost an average 35.3 gcHaps/locus and 2.8 major gcHaps/locus that were present in the LANs-*Geng* but absent in the MVs-*Geng*, while the MVs-*Geng* gained an average 13.5 new gcHaps/locus and 3.0 new major gcHaps that were present in the MVs-*Geng* but absent in the LANs-*Geng* (**Table 1**). The new gcHaps were apparently generated by intra-genic recombination during breeding, while the new major gcHaps were all come from minor rare gcHaps in the population LANs-*Geng*. Significant shifts in frequencies of the predominant gcHaps, F_(P)_, were observed at 12 of the 24 *OsGLR* loci, including significantly reduced F_(P)_ at *OsGLR6.7*, *OsGLR6.9*, *OsGLR7.1* and *OsGLR9.7* and significantly increased F_(P)_ at *OsGLR6.4*, *OsGLR6.5*, *OsGLR6.6*, *OsGLR6.8*, *OsGLR6.10*, *OsGLR9.2*, *OsGLR9.5* and *OsGLR7.2* (**Supplementary Data 7**). It should be pointed out that populations *Xian* and *Geng* have different predominant gcHaps at all polymorphic GLR loci, except for *OsGLR6.1* and *OsGLR9.4*. This was because there is only a single predominant in both populations *Xian* and *Geng* at *OsGLR6.1* while there is absence of any gcHaps at *OsGLR9.4* whose frequency was higher than 1% in population *Xian*.

### Associations of the major gcHaps at rice GLR genes with important agronomic traits

**Figure 8:**
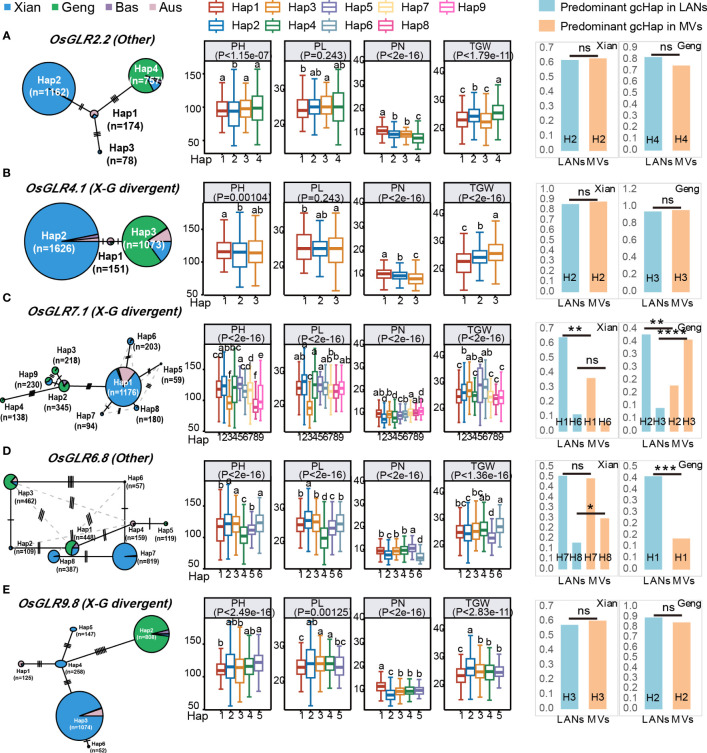
Haplotype networks of five GLR genes with CRSPR-9 mediated knocking-out and their associations with four agronomic traits in 3KRG. **(A)** Based on gcHap diversity and contribution to subspecific/population differentiation, the rice *OsGLR2.2* is classified as the other gene. **(B)** The *OsGLR4.1* is classified as a *X-G* divergent gene. **(C)** The *OsGLR7.1* is classified as a X-G divergent gene. **(D)** The rice *OsGLR6.8* is classified as the other gene. **(E)** The *OsGLR9.8* is classified as a *X-G* divergent gene. Within each haplotype network, two adjacent gcHaps are separated by mutational changes with hatching line indicating differences between the two most related haplotypes. The right side of each gene haplotype network corresponds to the phenotypic variation among the haplotypes. Boxplots are shown for the following four traits: plant height (PH), panicle length (PL), panicle number (PN) and 1000-grain weight (TGW). The *P* values under trait names indicate differences between the haplotypes assessed by two-way ANOVA, with different letters on the boxplots indicating statistically significant differences at *P* < 0.05 based on Duncan’s multiple range test. The bar charts on the right show the differences in frequency of the predominant gcHaps between landraces (LANs) and modern varieties (MVs) in *Xian* and *Geng*. Chi-square tests were used to determine significant differences in the proportions of the same gcHap between the different populations with ****P < 0.0001, ***P < 0.0001, **P < 0.01, *P < 0.05, and n.s., not significant.

To provide additional evidence for the functional importance of the 24 polymorphic GLR genes, we constructed the gcHap networks of the major alleles at 24 GLR genes in the four major rice populations, and analyzed the associations between the major gcHaps at each of the 24 *GLR* gene loci with phenotypic values of four agronomic traits, plant height (PH), panicle length (PL), panicle number per plant (PN) and thousand grain weight (TGW) in 3KRG (**Figure 8**; **Supplementary Figures 4**-**7**). Strong (P < 10^-7^) associations were observed in 61 (63.5%) of the 96 (24 x 4) cases and major alleles at many of the GLR gene were strongly associated with trait values of one or more traits. Notably, major alleles at *OsGLR2.2*, *OsGLR4.1*, *OsGLR7.1*, *OsGLR6.8* and *OsGLR9.8* were strongly associated with trait values of all four traits, except for PL in two cases (**Figure 8**), consistent with the results from above knock-out experiments (**Figure 4**). In particular, major alleles at ten GLR loci (*OsGLR6.4*, *OsGLR6.5*, *OsGLR6.6*, *OsGLR6.7*, *OsGLR6.8*, *OsGLR6.9*, *OsGLR6.10*, *OsGLR6.12 OsGLR7.1* and *OsGLR7.2*) were strongly associated with trait values of all four traits (**Figure 8** **;** **Supplementary Figures 4**-**7**). In contrast, no associations were detected for major alleles at *OsGLR6.1* and *OsGLR9.1* with the trait values of the four traits, largely because of too few major gcHaps present at the three loci (**Supplementary Data 4**).

### Comparison of trait values between the predominant and ‘unfavorable’ gcHaps of GLR genes in rice

In contrast to the predominant gcHap (with highest frequency) at a GLR locus in a rice population would be considered to be favored by natural selection during evolution, the major gcHap with the lowest frequencies in the population would more likely to be the ‘unfavorable’ one. To test if this scenario was holding true, we compared the phenotypic differences between the predominant gcHap and ‘unfavorable’ one at each of the 24 GLR loci for 15 agronomic traits. Of the total 24 x 15 = 360 comparisons, significant differences were detected in 149 (41.4%) cases between the predominant gcHaps and unfavorable at all GLR gene loci except for *OsGLR6.8*, *OsGLR9.7* and *OsGLR9.8* (**Supplementary Figure 8**-**31**; **Supplementary Data 8**). Of the 15 traits, DTH was detected at 16 (66.7%) of the GLR loci, followed by PN at 14 (58.3%) of the GLR loci, consistent with the result of the gene knockout experiments that the five knockout GLR genes had large and consistent effects on PN and their phenotypic effects were influenced strongly by the two environments of contrasting day-lengths that are known to great influence on DTH of different rice varieties (**Figure 4**). Across the 24 GLR loci, significant differences in mean trait values between the predominant gcHap and unfavorable one were detected for almost all traits for *OsGLR9.1* (14 traits), *OsGLR9.9* (14 traits), and *OsGLR9.2* (13 traits), indicating these three GLR genes were more likely affecting more traits than other GLR loci (**Supplementary Data 8**).

### Mining ‘favorable’ allele(s) of rice GLR loci for improved productivity

**Figure 9:**
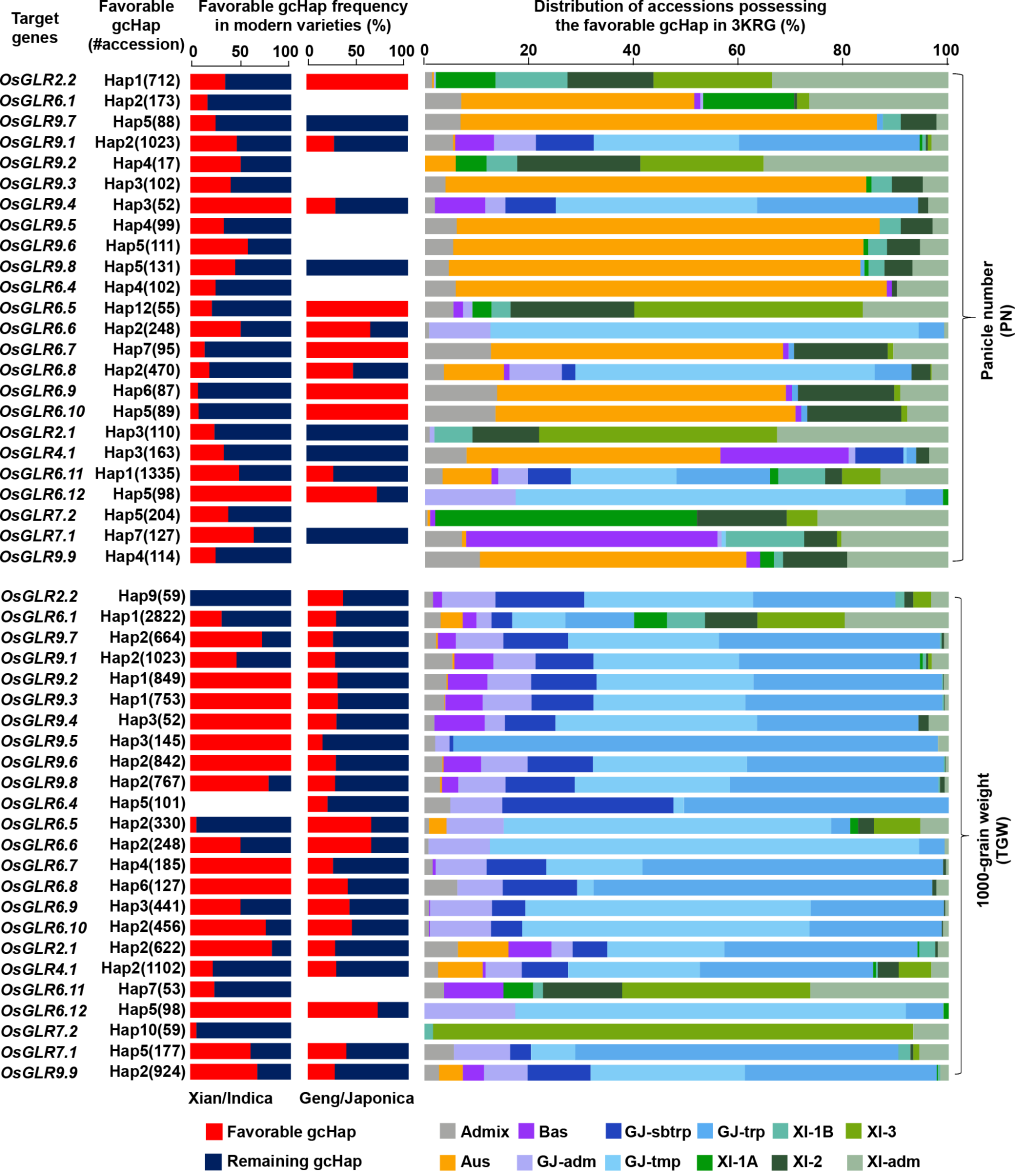
Frequencies of the favorable gcHaps of all rice GLR genes affecting important agronomic traits (PN and TGW) in *Xian/indica* (*XI*) and *Geng/japonica* (*GJ*) modern varieties and different rice subpopulations. The favorable gcHap of a gene is defined as the gcHap associated with the highest trait value. “#accession” indicates the number of accessions that possess the favorable gcHap. Five subpopulations of *XI* (*XI-1A*, *XI-1B*, *XI-2*, *XI-3*, and *XI-adm*) and four subpopulations of *GJ* (temperate *GJ*: *GJ-tmp*, subtropical *GJ*: *GJ-sbtrp*, tropical *GJ*: *GJ-trp*, and *GJ-adm*) were classified by Wang et al. (Wang et al., 2018).

As described above, there were significant differences between or among the major gcHaps at most rice GLR loci regarding their effects on traits of agronomic importance. Thus, it is reasonable to assume the presence of ‘favorable’ gcHap(s) at most GLR loci for traits leading to increased productivity, even though the ‘favorable’ gcHap(s) may differ across different target environments. Thus, there should be one or more ‘favorable’ alleles, defined as the gcHap(s) associated with trait values for high productivity. Then, the frequencies of these most ‘favorable’ gcHaps in the MVs and different rice populations would of great interest for rice breeders. **Figure 9** and **Supplementary Figure 32** show the frequencies of the ‘favorable’ gcHaps at 24 rice GLR genes that affects PN, TGW, PH and PL in populations MVs-*Xian* and MVs-*Geng* and the four major rice populations of 3KRG. The general observation was that the ‘favorable’ allele frequency varied considerably across different yield traits, different GLR loci, and across populations MVs-*Xian* and MVs-*Geng* or different rice populations. For example, the Hap3 at *OsGLR9.2* and Hap5 at *OsGLR6.12* were associated with the highest PN in 3KRG accessions, which reached fixation in MVs-*Xian*, but had low frequencies in MVs-*Geng* (**Figure 9**). The same was true for eight GLR loci (*OsGLR9.2* - *OsGLR9.6*, *OsGLR6.7*, *OsGLR6.8* and *OsGLR6.12*) with the highest TGW. In contrast, Hap1 at *OsGLR2.2*, Hap12 at *OsGLR6.5*, Hap6 at *OsGLR6.9*, and Hap5 at *OsGLR6.10* were associated with the highest PN in 3KRG accessions, which reached fixation in MVs-*Geng*, but had low frequencies in MVs-*Xian*. This was consistent with the empirical observation in rice breeding that the extremely high values for any specific yield components may not generally result in the highest productivity. This result also suggested that the ‘favorable’ alleles for different yield traits could be very different across the two rice subspecies or genetic backgrounds and possibly across different environments.

## Discussion

Although glutamate receptors are specialized proteins that perform a crucial role in signal transduction and communication in both animals and plants. Present as a large gene family in the rice genome, the molecular properties and functional characteristics of most rice GLR genes remain largely unknown. The phylogenetic analysis based on sequence similarity classified the 26 rice GLR genes into four major groups with groups II and III closely related to maize GLR genes, while group IV containing eight GLR genes clustered on rice chromosome 6 was unique ones only present rice only (**Figure 1**). In this study, we demonstrated an integrated strategy by limited experimental efforts plus comprehensive data mining and analyses to understand the functionalities of the rice GLR genes, focusing on the phenotypic characterization and genetic diversity of rice GLR genes because of their importance for potential utilization in rice improvement. In this regard, our results revealed several important properties of the rice GLR genes that led us to several conclusions on the functionalities and evolutionary history of rice GLR genes.

Our first conclusion was that the expansion of the rice GLR genes into a large gene family during evolution had gone through repeated gene duplication events evidenced by the presence of two large GLR gene clusters on rice chromosomes 9 and 6, and their greater gcHap diversity and rapid evolutionary rates (**Table 1**), and this expansion of the rice GLR genes had been accompanied with considerable functional differentiation, evidenced by their different tissue expression specificities (**Figure 2**), by their differentiated responses to different combinations of abiotic stresses and hormone treatments (**Figure 3**), and by their associations with different combinations of agronomic traits (**Supplementary Figures 8**-**31**).

Secondly, rice GLR genes may have played an important roles in rice sub-specific and population differentiation during evolution, evidenced by their very strong sub-specific and population differentiation at most rice GLR loci (**Figure 6**), by the great differentiated responses to day-length, the major factor determining rice subspecific adaptations to their environments (**Figure 4**), and by their differentiated responses to different abiotic stresses (**Figure 3**). In fact, the observed population differentiation at most GLR loci were far stronger than most of the 3,000+ cloned rice genes or genomewide SNPs analyzed previously (Wang et al., 2018; Zhang et al., 2021).

Thirdly, many rice GLR genes may have played an important role in rice improvement, suggested by the strong and large phenotypic effects of the five GLR gene knockout mutants on rice yield traits (**Figure 4**), by the significant shifts of the predominant gcHaps and diversity changes at many rice GLR loci (**Figures 5** **–** **7**, **Table 1**), and by their strong associations of the major gcHaps with agronomic traits at many GLR loci (**Figures 8** **–** **9**; **Supplementary data 8**). This is not surprising since acting as signal receptors, GLR genes are expected to play important regulatory roles involved in many processes of rice growth and development and their responses to environmental cues. However, it remains to be elucidated in future how different rice GLR genes are involved in these important processes at the molecular level.

Fourthly, the observed diversity increases at some of the GLR loci during breeding were somehow unexpected since modern rice breeding is believed by many to cause reduced diversity in MVs. For example, the convergent phenotypic selection during breeding for similar phenotypes associated for high productivity in virtually all breeding programs, which include more compact plant type (erect leaves and smaller culm and leaf angles), large panicles (more), moderate sizes of TGW, high SR, more PN, and tolerance/resistance to the common abiotic/biotic stresses, etc. Also, the fact that only a very small portion of germplasm accessions maintained in genebanks worldwide have been utilized as parents in specific breeding programs would expected to result in severe genetic bottleneck and thus reduced genetic diversity at most gene loci. Indeed, we observed average losses of 39.3% (20.8%) of gcHaps (major gcHaps) at the 24 GLR loci during *Xian* breeding, which were accompanied with the emergences of 62.8 (18.4%) and new gcHaps per locus and 2.2 (28.6%) new major gcHaps/locus in MVs-*Xian* (**Figure 7**). The average losses of gcHaps (major gcHaps) at the 24 GLR loci reached were pronounced during *Geng* breeding, reaching 46.3% (58.3%), which were accompanied with an average emergences of 13.5 (17.7%) new gcHaps/locus and 3.0 (62.5%) new major gcHaps/locus during *Geng* breeding. In other words, the losses of gcHaps from the genetic bottleneck effects during modern breeding were more severe for MVs-*Geng* than MVs-*Xian*. This was expected since line hybridization is a common practice of modern breeding, which would result in greatly increased recombination as compared to the extremely low outcrossing rate in rice landraces. The much greater numbers of gcHap losses/gains observed in the two large GLR clusters on chromosomes 9 and 6 (**Table 1**) strongly suggest that more frequently occurred unequal crossing-over events in the large gene clusters would not only generate gene copy number variation but also new alleles of the duplicated GLR genes. More interestingly, significant reduced frequencies of the predominant gcHaps, losses of a few major gcHaps and the emergences of new major gcHaps in MVs-*Xian* and MVs-*Geng* from the rare ones in LANs-*Xian* and LANs-*Geng* at specific GLR loci would suggest cases that the predominant and/or major gcHaps favored by natural selection were against by the artificial selection during modern breeding. This also implies that many rare gcHaps might be of value in rice improvement. Taken together, modern breeding activities in the past had the greater impact on the GLR gene loci than the 3,000+ cloned genes analyzed previously (Zhang et al., 2021), implying again the greater importance of the GLR genes in rice improvement.

Finally, our results would suggest the potential values of the natural variation at most rice GLR loci for improving the productivity and tolerances to abiotic stresses. All rice GLR genes, except for *OsGLR6.2*, *OsGLR6.3*, *OsGLR6.1* and *OsGLR9.1*, are potential values to be utilized in rice trait improvement. Specifically, *OsGLR2.1*, *OsGLR2.2*, *OsGLR4.1*, *OsGLR6.12*, *OsGLR7.1* and *OsGLR9.8* could potentially be used for improving rice tolerances to abiotic stresses, while others can potentially be used for improving rice yield traits. However, challenges remain in the identification of ‘favorable’ alleles at specific GLR loci for improving specific yield traits because of the greater G x E interaction of most rice GLR genes on yield traits observed in this study. Thus, additional efforts are needed to obtain the required information in order to facilitate their application to improving rice productivity in future.

## Data availability statement

The datasets presented in this study can be found in online repositories. The names of the repository/repositories and accession number(s) can be found in the article/**Supplementary Material**.

## Author contributions

YS and ZL designed the experiments. WZ, HL, FZ, XW, SR, SH, CheZ, FW, JL, YL, ChaZ, ML, and YS performed the experiments and data analyses. ZL, WZ, and YS wrote the manuscript. All authors contributed to the article and approved the submitted version.

## Supplementary material

Supplementary Data Sheet 1 Sequence information of plant and animal GLR genes.

Supplementary Figure 1 Gene structures of knockout mutants of five rice *OsGLR* genes. **(A-E)** The genes represented by **(A-E)** are *OsGLR*2.2, *OsGLR*9.8, *OsGLR*6.8, *OsGLR4.1* and *OsGLR7.1*.

Supplementary Figure 2 Classification of gene categories of rice *OsGLR* genes based on their gcHap diversity and contribution to subspecific/population differentiation, with conserved genes (*E_H_* < 0.3 in 3KRG and *I_Nei_* ≥ 0.8 between pairwise populations) and the *Xian*-*Geng* (*X-G*) divergent genes (*E_H_* < 0.3 in both Xian and Geng and *I_Nei_* ≤ 0.2 between *Xian* and *Geng*).

Supplementary Figure 3 Frequency shifts in the predominant gcHaps at 24 *OsGLR* genes between the landraces and modern varieties.

Supplementary Figure 4 Haplotype networks of five overexpressed *OsGLR* genes and their associations with four agronomic traits in 3KRG. Within each haplotype network, two adjacent gcHaps are separated by mutational changes with hatching line indicating differences between the two most related haplotypes. The right side of each gene haplotype network corresponds to the phenotypic variation among the haplotypes. Boxplots are shown for the following four traits: plant height (PH), panicle length (PL), panicle number (PN) and 1000-grain weight (TGW). The *P* values under trait names indicate differences between the haplotypes assessed by two-way ANOVA, with different letters on the boxplots indicating statistically significant differences at *P* < 0.05 based on Duncan’s multiple range test. The bar charts on the right show the differences in frequency of the predominant gcHaps between landraces (LANs) and modern varieties (MVs) in *Xian* and *Geng*. Chi-square tests were used to determine significant differences in the proportions of the same gcHap between the different populations with *****P* < 0.0001, ***P* < 0.01, **P* < 0.05, and n.s., not significant.

Supplementary Figure 5 Haplotype networks of four GLR genes and their associations with four agronomic traits in 3KRG. Within each haplotype network, two adjacent gcHaps are separated by mutational changes with hatching line indicating differences between the two most related haplotypes. The right side of each gene haplotype network corresponds to the phenotypic variation among the haplotypes. Boxplots are shown for the following four traits: plant height (PH), panicle length (PL), panicle number (PN) and 1000-grain weight (TGW). The *P* values under trait names indicate differences between the haplotypes assessed by two-way ANOVA, with different letters on the boxplots indicating statistically significant differences at *P* < 0.05 based on Duncan’s multiple range test. The bar charts on the right show the differences in frequency of the predominant gcHaps between landraces (LANs) and modern varieties (MVs) in *Xian* and *Geng*. Chi-square tests were used to determine significant differences in the proportions of the same gcHap between the different populations with *****P* < 0.0001, ***P* < 0.01, **P* < 0.05, and n.s., not significant.

Supplementary Figure 6 Haplotype networks of five *OsGLR* genes and their associations with four agronomic traits in 3KRG. Within each haplotype network, two adjacent gcHaps are separated by mutational changes with hatching line indicating differences between the two most related haplotypes. The right side of each gene haplotype network corresponds to the phenotypic variation among the haplotypes. Boxplots are shown for the following four traits: plant height (PH), panicle length (PL), panicle number (PN) and 1000-grain weight (TGW). The *P* values under trait names indicate differences between the haplotypes assessed by two-way ANOVA, with different letters on the boxplots indicating statistically significant differences at *P* < 0.05 based on Duncan’s multiple range test. The bar charts on the right show the differences in frequency of the predominant gcHaps between landraces (LANs) and modern varieties (MVs) in *Xian* and *Geng*. Chi-square tests were used to determine significant differences in the proportions of the same gcHap between the different populations with *****P* < 0.0001, ***P* < 0.01, **P* < 0.05, and n.s., not significant.

Supplementary Figure 7 Haplotype networks of remaining GLR genes and their associations with four agronomic traits in 3KRG. Within each haplotype network, two adjacent gcHaps are separated by mutational changes with hatching line indicating differences between the two most related haplotypes. The right side of each gene haplotype network corresponds to the phenotypic variation among the haplotypes. Boxplots are shown for the following four traits: plant height (PH), panicle length (PL), panicle number (PN) and 1000-grain weight (TGW). The *P* values under trait names indicate differences between the haplotypes assessed by two-way ANOVA, with different letters on the boxplots indicating statistically significant differences at *P* < 0.05 based on Duncan’s multiple range test. The bar charts on the right show the differences in frequency of the predominant gcHaps between landraces (LANs) and modern varieties (MVs) in *Xian* and *Geng*. Chi-square tests were used to determine significant differences in the proportions of the same gcHap between the different populations with *****P* < 0.0001, ***P* < 0.01, **P* < 0.05, and n.s., not significant.

Supplementary Figure 8 Comparison and analysis of 15 agronomic traits among the predominant gcHap, unfavorable gcHap, and major gcHaps of *OsGLR2.1*.

Supplementary Figure 9 Comparison and analysis of 15 agronomic traits among the predominant gcHap, unfavorable gcHap, and major gcHaps of *OsGLR2.2*.

Supplementary Figure 10 Comparison and analysis of 15 agronomic traits among the predominant gcHap, unfavorable gcHap, and major gcHaps of *OsGLR4.1*.

Supplementary Figure 11 Comparison and analysis of 15 agronomic traits among the predominant gcHap, unfavorable gcHap, and major gcHaps of *OsGLR6.1*.

Supplementary Figure 12 Comparison and analysis of 15 agronomic traits among the predominant gcHap, unfavorable gcHap, and major gcHaps of *OsGLR6.4*.

Supplementary Figure 13 Comparison and analysis of 15 agronomic traits among the predominant gcHap, unfavorable gcHap, and major gcHaps of *OsGLR6.5*.

Supplementary Figure 14 Comparison and analysis of 15 agronomic traits among the predominant gcHap, unfavorable gcHap, and major gcHaps of *OsGLR6.6*.

Supplementary Figure 15 Comparison and analysis of 15 agronomic traits among the predominant gcHap, unfavorable gcHap, and major gcHaps of *OsGLR6.7*.

Supplementary Figure 16 Comparison and analysis of 15 agronomic traits among the predominant gcHap, unfavorable gcHap, and major gcHaps of *OsGLR6.8*.

Supplementary Figure 17 Comparison and analysis of 15 agronomic traits among the predominant gcHap, unfavorable gcHap, and major gcHaps of *OsGLR6.9*.

Supplementary Figure 18 Comparison and analysis of 15 agronomic traits among the predominant gcHap, unfavorable gcHap, and major gcHaps of *OsGLR6.10*.

Supplementary Figure 19 Comparison and analysis of 15 agronomic traits among the predominant gcHap, unfavorable gcHap, and major gcHaps of *OsGLR6.11*.

Supplementary Figure 20 Comparison and analysis of 15 agronomic traits among the predominant gcHap, unfavorable gcHap, and major gcHaps of *OsGLR6.12*.

Supplementary Figure 21 Comparison and analysis of 15 agronomic traits among the predominant gcHap, unfavorable gcHap, and major gcHaps of *OsGLR7.1*.

Supplementary Figure 22 Comparison and analysis of 15 agronomic traits among the predominant gcHap, unfavorable gcHap, and major gcHaps of *OsGLR7.2*.

Supplementary Figure 23 Comparison and analysis of 15 agronomic traits among the predominant gcHap, unfavorable gcHap, and major gcHaps of *OsGLR9.1*.

Supplementary Figure 24 Comparison and analysis of 15 agronomic traits among the predominant gcHap, unfavorable gcHap, and major gcHaps of *OsGLR9.2*.

Supplementary Figure 25 Comparison and analysis of 15 agronomic traits among the predominant gcHap, unfavorable gcHap, and major gcHaps of *OsGLR9.3*.

Supplementary Figure 26 Comparison and analysis of 15 agronomic traits among the predominant gcHap, unfavorable gcHap, and major gcHaps of *OsGLR9.4*.

Supplementary Figure 27 Comparison and analysis of 15 agronomic traits among the predominant gcHap, unfavorable gcHap, and major gcHaps of *OsGLR9.5*.

Supplementary Figure 28 Comparison and analysis of 15 agronomic traits among the predominant gcHap, unfavorable gcHap, and major gcHaps of *OsGLR9.6*.

Supplementary Figure 29 Comparison and analysis of 15 agronomic traits among the predominant gcHap, unfavorable gcHap, and major gcHaps of *OsGLR9.7*.

Supplementary Figure 30 Comparison and analysis of 15 agronomic traits among the predominant gcHap, unfavorable gcHap, and major gcHaps of *OsGLR9.8*.

Supplementary Figure 31 Comparison and analysis of 15 agronomic traits among the predominant gcHap, unfavorable gcHap, and major gcHaps of *OsGLR9.9*.

Supplementary Figure 32 Frequencies of the favorable gcHaps of all rice *OsGLR* genes affecting important agronomic traits (PH and PL) in *Xian/indica* (*XI*) and *Geng/japonica* (*GJ*) modern varieties and different rice subpopulations. The favorable gcHap of a gene was defined as the gcHap associated with the highest trait value. “#accession” indicates the number of accessions that possess the favorable gcHap. Five subpopulations of *XI* (*XI-1A*, *XI-1B*, *XI-2*, *XI-3*, and *XI-adm*) and four subpopulations of *GJ* (temperate *GJ*: *GJ-tmp*, subtropical *GJ*: *GJ-sbtrp*, tropical *GJ*: *GJ-trp*, and *GJ-adm*) were classified by Wang et al. (Wang et al., 2018).

## References

[B1] Cheng Y. Tian Q. Zhang W. H. (2016). Glutamate receptors are involved in mitigating effects of amino acids on seed germination of *Arabidopsis thaliana* under salt stress. Environ. Exp. Bot. 130, 68–78. doi: 10.1016/j.envexpbot.2016.05.004

[B2] Cheng Y. Zhang X. Sun T. Tian Q. Zhang W. H. (2018). Glutamate receptor homolog3.4 is involved in regulation of seed germination under salt stress in *Arabidopsis* . Plant Cell Physiol. 59 (5), 978–988. doi: 10.1093/pcp/pcy034 29432559

[B3] Green M. N. Gangwar S. P. Michard E. Simon A. A. Portes M. T. Barbosa-Caro J.. (2021). Structure of the *Arabidopsis thaliana* glutamate receptor-like channel GLR3.4. Mol. Cell 81 (15), 3216–3226.e3218. doi: 10.1016/j.molcel.2021.05.025 34161757 PMC8349882

[B4] Kang J. Turano F. J. (2003). The putative glutamate receptor 1.1 (AtGLR1.1) functions as a regulator of carbon and nitrogen metabolism in *Arabidopsis thaliana* . Proc. Natl. Acad. Sci. U.S.A. 100 (11), 6872–6877. doi: 10.1073/pnas.1030961100 12738881 PMC164539

[B5] Kong D. Ju C. Parihar A. Kim S. Cho D. Kwak J. M. (2015). Arabidopsis glutamate receptor homolog3.5 modulates cytosolic Ca2+ level to counteract effect of abscisic acid in seed germination. Plant Physiol. 167 (4), 1630–1642. doi: 10.1104/pp.114.251298 25681329 PMC4378146

[B6] Lam H. M. Chiu J. Hsieh M. H. Meisel L. Oliveira I. C. Shin M.. (1998). Glutamate-receptor genes in plants. Nature 396 (6707), 125–126. doi: 10.1038/24066 9823891

[B7] Li H. Jiang X. Lv X. Ahammed G. J. Guo Z. Qi Z.. (2019). Tomato GLR3.3 and GLR3.5 mediate cold acclimation-induced chilling tolerance by regulating apoplastic H(2) O(2) production and redox homeostasis. Plant Cell Environ. 42 (12), 3326–3339. doi: 10.1111/pce.13623 31329293

[B8] Li J. Zhu S. H. Song X. W. Shen Y. Chen H. M. Yu J.. (2006). A rice glutamate receptor-like gene is critical for the division and survival of individual cells in the root apical meristem. Plant Cell 18 (2), 340–349. doi: 10.1105/tpc.105.037713 16377757 PMC1356543

[B9] Liu S. Zhang X. Xiao S. Ma J. Shi W. Qin T.. (2021). A single-nucleotide mutation in a *GLUTAMATE RECEPTOR-LIKE* gene confers resistance to fusarium wilt in *Gossypium hirsutum* . Adv. Sci. (Weinh) 8 (7), 2002723. doi: 10.1002/advs.202002723 33854882 PMC8025038

[B10] Lu G. H. Wang X. P. Liu J. H. Yu K. Gao Y. Liu H. Y.. (2014). Application of T-DNA activation tagging to identify *glutamate receptor-like* genes that enhance drought tolerance in plants. Plant Cell Rep. 33 (4), 617–631. doi: 10.1007/s00299-014-1586-7 24682459

[B11] Michard E. Lima P. T. Borges F. Silva A. C. Portes M. T. Carvalho J. E.. (2011). *Glutamate receptor-like* genes form Ca2+ channels in pollen tubes and are regulated by pistil D-serine. Science 332 (6028), 434–437. doi: 10.1126/science.1201101 21415319

[B12] Mousavi S. A. R. Chauvin A. Pascaud F. Kellenberger S. Farmer E. E. (2013). *GLUTAMATE RECEPTOR-LIKE* genes mediate leaf-to-leaf wound signalling. Nature 500 (7463), 422–426. doi: 10.1038/nature12478 23969459

[B13] Nei M. (1972). Genetic distance between populations. Am. Nat. 106 (949), 283–292. doi: 10.1086/282771

[B14] Paradis E. (2010). pegas: an R package for population genetics with an integrated-modular approach. Bioinformatics 26 (3), 419–420. doi: 10.1093/bioinformatics/btp696 20080509

[B15] Philippe F. Verdu I. Morere-Le Paven M. C. Limami A. M. Planchet E. (2019). Involvement of Medicago truncatula glutamate receptor-like channels in nitric oxide production under short-term water deficit stress. J. Plant Physiol. 236, 1–6. doi: 10.1016/j.jplph.2019.02.010 30836205

[B16] Project, r.g (2014). The 3,000 rice genomes project. Gigascience 3, 7. doi: 10.1186/2047-217X-3-7 24872877 PMC4035669

[B17] Shao Q. Gao Q. Lhamo D. Zhang H. Luan S. (2020). Two glutamate- and pH-regulated Ca(2+) channels are required for systemic wound signaling in Arabidopsis. Sci. Signal 13 (640), eaba1453. doi: 10.1126/scisignal.aba1453 32665412

[B18] Sheldon A. L. (1969). Equitability indices: dependence on the species count. Ecology 50 (3), 466–467. doi: 10.2307/1933900

[B19] Shi Y. Zeng W. Xu M. Li H. Zhang F. Chen Z.. (2022). Comprehensive Analysis of *Glutamate Receptor-like* Genes in Rice (Oryza sativa L.): Genome-Wide Identification, Characteristics, Evolution, Chromatin Accessibility, gcHap Diversity, Population Variation and Expression Analysis. Curr. Issues Mol. Biol. 44 (12), 6404–6427. doi: 10.3390/cimb44120437 36547098 PMC9777005

[B20] Simon A. A. Navarro-Retamal C. Feijó J. A. (2023). Merging signaling with structure: functions and mechanisms of plant glutamate receptor ion channels. Annu. Rev. Plant Biol 74, 415–452. doi: 10.1146/annurev-arplant-070522-033255 PMC11479355 36854472

[B21] Singh S. K. Chien C. T. Chang I. F. (2016). The Arabidopsis glutamate receptor-like gene *GLR3.6* controls root development by repressing the Kip-related protein gene KRP4. J. Exp. Bot. 67 (6), 1853–1869. doi: 10.1093/jxb/erv576 26773810

[B22] Templeton A. R. Crandall K. A. Sing C. F. (1992). A cladistic analysis of phenotypic associations with haplotypes inferred from restriction endonuclease mapping and DNA sequence data. III. Cladogram estimation. Genetics 132 (2), 619–633. doi: 10.1093/genetics/132.2.619 1385266 PMC1205162

[B23] Vincill E. D. Clarin A. E. Molenda J. N. Spalding E. P. (2013). Interacting glutamate receptor-like proteins in Phloem regulate lateral root initiation in Arabidopsis. Plant Cell 25 (4), 1304–1313. doi: 10.1105/tpc.113.110668 23590882 PMC3663269

[B24] Wang P. H. Lee C. E. Lin Y. S. Lee M. H. Chen P. Y. Chang H. C.. (2019). The glutamate receptor-like protein GLR3.7 interacts with 14-3-3ω and participates in salt stress response in *Arabidopsis thaliana* . Front. Plant Sci. 10. doi: 10.3389/fpls.2019.01169 PMC6779109 31632419

[B25] Wang W. Mauleon R. Hu Z. Chebotarov D. Tai S. Wu Z.. (2018). Genomic variation in 3,010 diverse accessions of Asian cultivated rice. Nature 557 (7703), 43–49. doi: 10.1038/s41586-018-0063-9 29695866 PMC6784863

[B26] Wudick M. M. Portes M. T. Michard E. Rosas-Santiago P. Lizzio M. A. Nunes C. O.. (2018). CORNICHON sorting and regulation of GLR channels underlie pollen tube Ca(2+) homeostasis. Science 360 (6388), 533–536. doi: 10.1126/science.aar6464 29724955

[B27] Xia L. Zou D. Sang J. Xu X. Yin H. Li M.. (2017). Rice Expression Database (RED): An integrated RNA-Seq-derived gene expression database for rice. J. Genet. Genomics 44 (5), 235–241. doi: 10.1016/j.jgg.2017.05.003 28529082

[B28] Xue N. Zhan C. Song J. Li Y. Zhang J. Qi J.. (2022). The glutamate receptor-like 3.3 and 3.6 mediate systemic resistance to insect herbivores in Arabidopsis. J. Exp. Bot. 73 (22), 7611–7627. doi: 10.1093/jxb/erac399 36214841 PMC9730813

[B29] Yu B. Wu Q. Li X. Zeng R. Min Q. Huang J. (2022). GLUTAMATE RECEPTOR-like gene *OsGLR3.4* is required for plant growth and systemic wound signaling in rice (*Oryza sativa*). New Phytol. 233 (3), 1238–1256. doi: 10.1111/nph.17859 34767648

[B30] Zeng W. Shi J. Qiu C. Wang Y. Rehman S. Yu S.. (2020). Identification of a genomic region controlling thermotolerance at flowering in maize using a combination of whole genomic re-sequencing and bulked segregant analysis. Theor. Appl. Genet. 133 (10), 2797–2810. doi: 10.1007/s00122-020-03632-x 32535640

[B31] Zhang F. Wang C. Li M. Cui Y. Shi Y. Wu Z.. (2021). The landscape of gene-CDS-haplotype diversity in rice: Properties, population organization, footprints of domestication and breeding, and implications for genetic improvement. Mol. Plant 14 (5), 787–804. doi: 10.1016/j.molp.2021.02.003 33578043

[B32] Zheng Y. Luo L. Wei J. Chen Q. Yang Y. Hu X.. (2021). Corrigendum to: The glutamate receptors AtGLR1.2 and AtGLR1.3 increase cold tolerance by regulating jasmonate signaling in *Arabidopsis thaliana* . Biochem. Biophys. Res. Commun. 566, 211–213. doi: 10.1016/j.bbrc.2021.06.061 34176593

