# Supplementary figures and images for: Functional characterization and allelic mining of OsGLR genes for potential uses in rice improvement
Source: Front Plant Sci. 2023 Aug 11;14:1236251. doi: 10.3389/fpls.2023.1236251 (PMC10450912; doi:10.3389/fpls.2023.1236251)

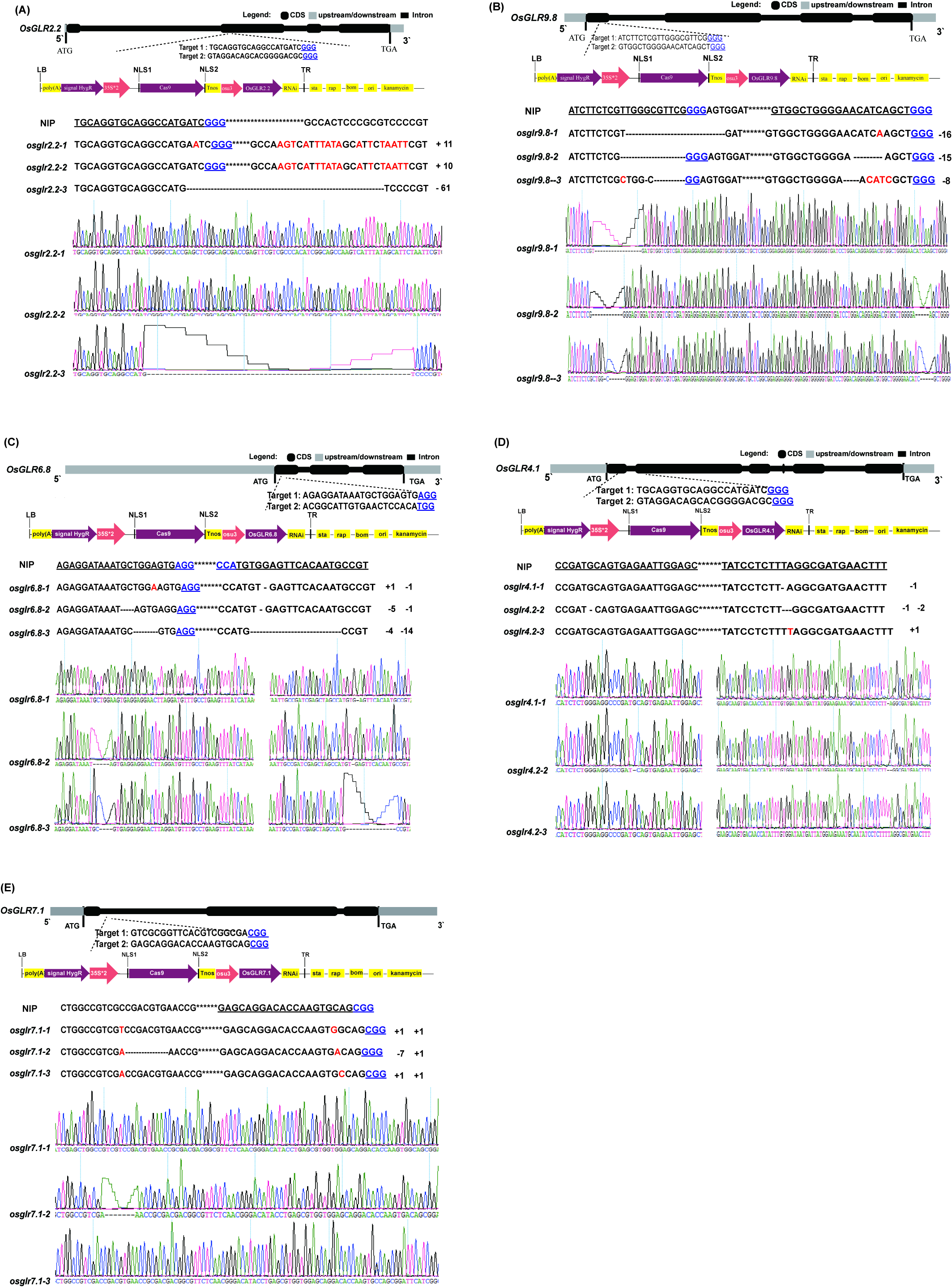

Supplement: Supplementary Figure 1 — Gene structures of knockout mutants of five rice OsGLR genes. (A-E) The genes represented by (A-E) are OsGLR2.2, OsGLR9.8, OsGLR6.8, OsGLR4.1 and OsGLR7.1. [file DataSheet_3.zip › Supplementary Figure 1 Gene knockout experiments on five rice glutamate-like receptor genes.tif]

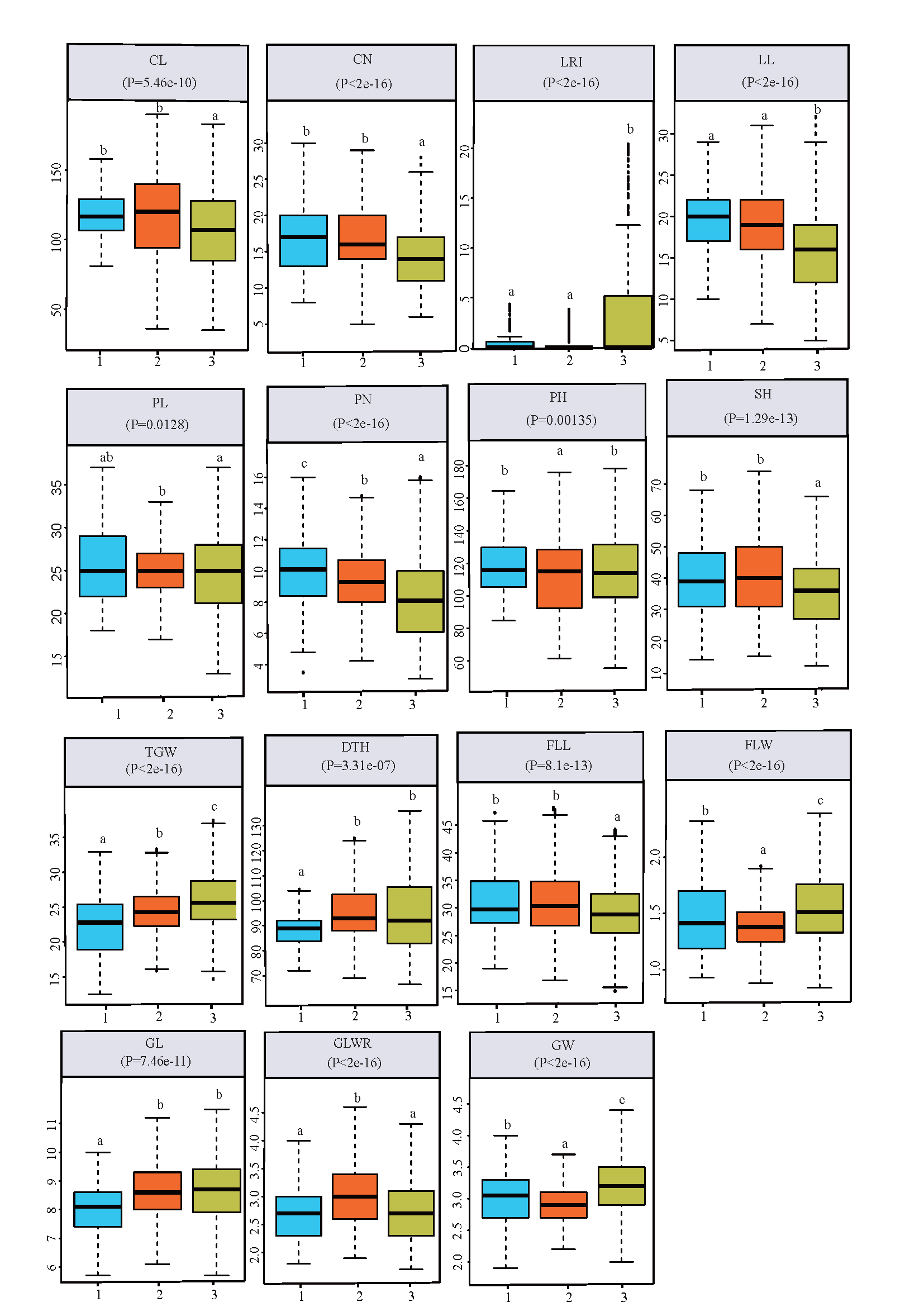

Supplement: Supplementary Figure 1 — Gene structures of knockout mutants of five rice OsGLR genes. (A-E) The genes represented by (A-E) are OsGLR2.2, OsGLR9.8, OsGLR6.8, OsGLR4.1 and OsGLR7.1. [file DataSheet_3.zip › Supplementary Figure 10 glr4_1.tif]

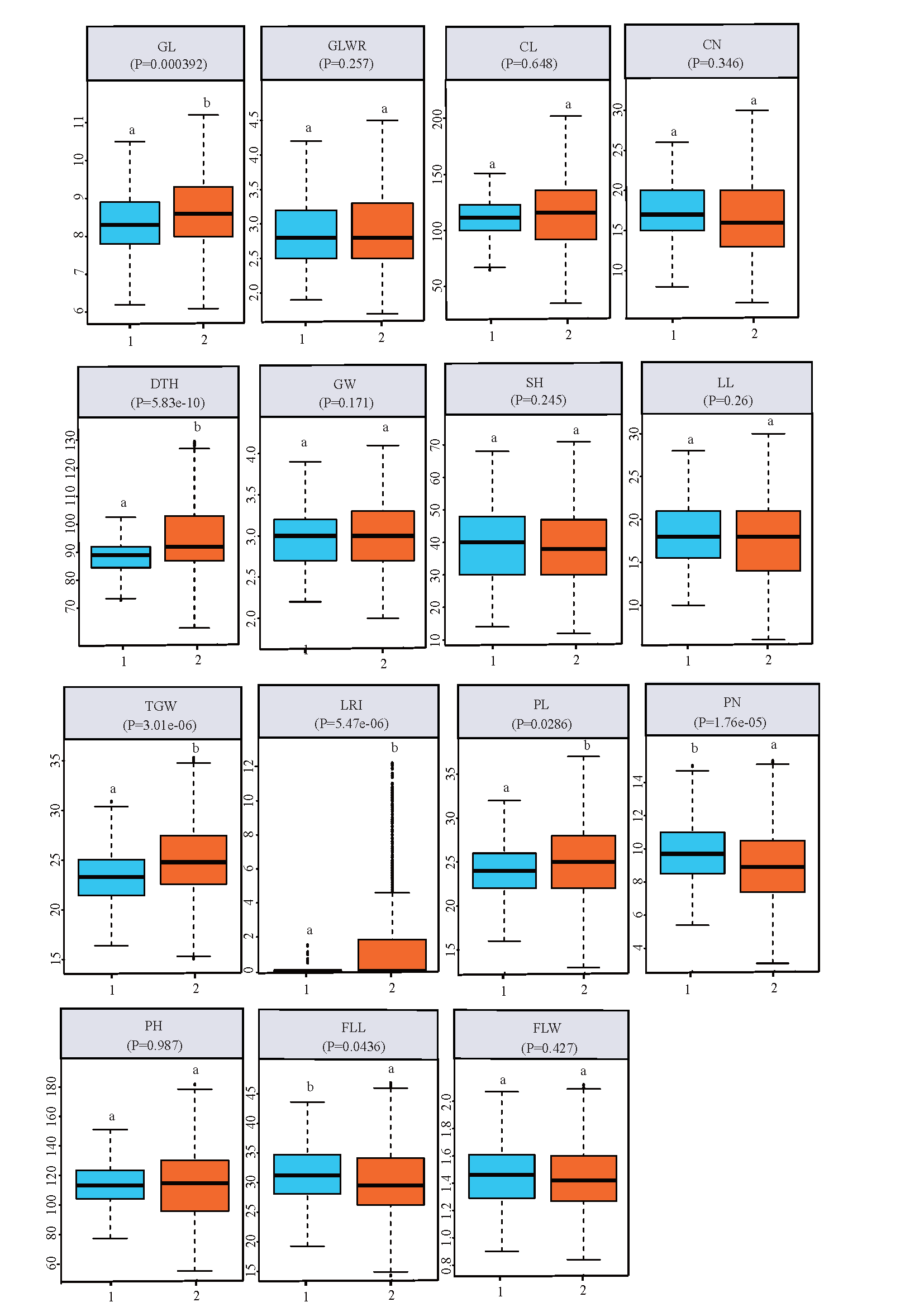

Supplement: Supplementary Figure 1 — Gene structures of knockout mutants of five rice OsGLR genes. (A-E) The genes represented by (A-E) are OsGLR2.2, OsGLR9.8, OsGLR6.8, OsGLR4.1 and OsGLR7.1. [file DataSheet_3.zip › Supplementary Figure 11 glr6_1.tif]

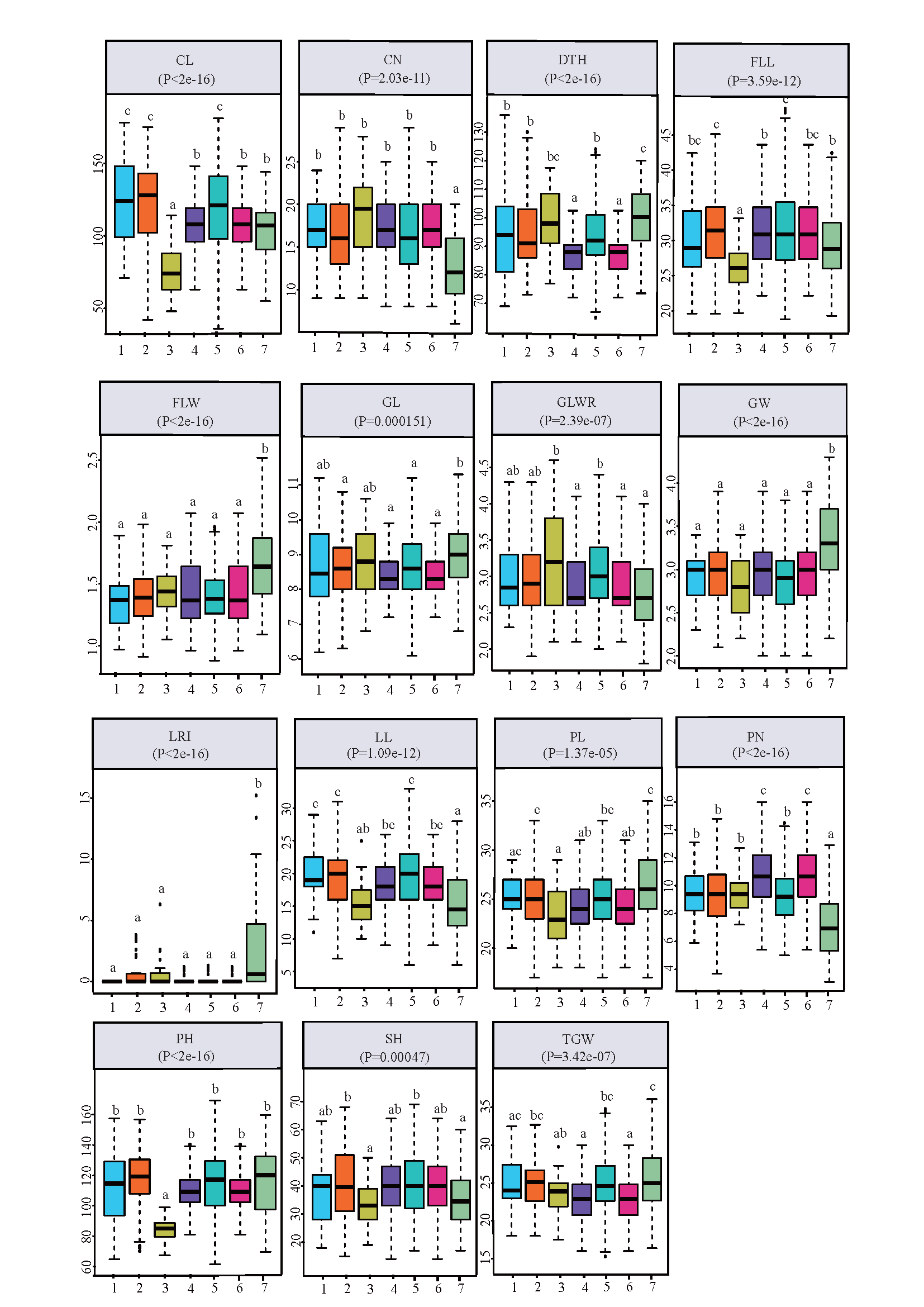

Supplement: Supplementary Figure 1 — Gene structures of knockout mutants of five rice OsGLR genes. (A-E) The genes represented by (A-E) are OsGLR2.2, OsGLR9.8, OsGLR6.8, OsGLR4.1 and OsGLR7.1. [file DataSheet_3.zip › Supplementary Figure 12 glr6_4.tif]

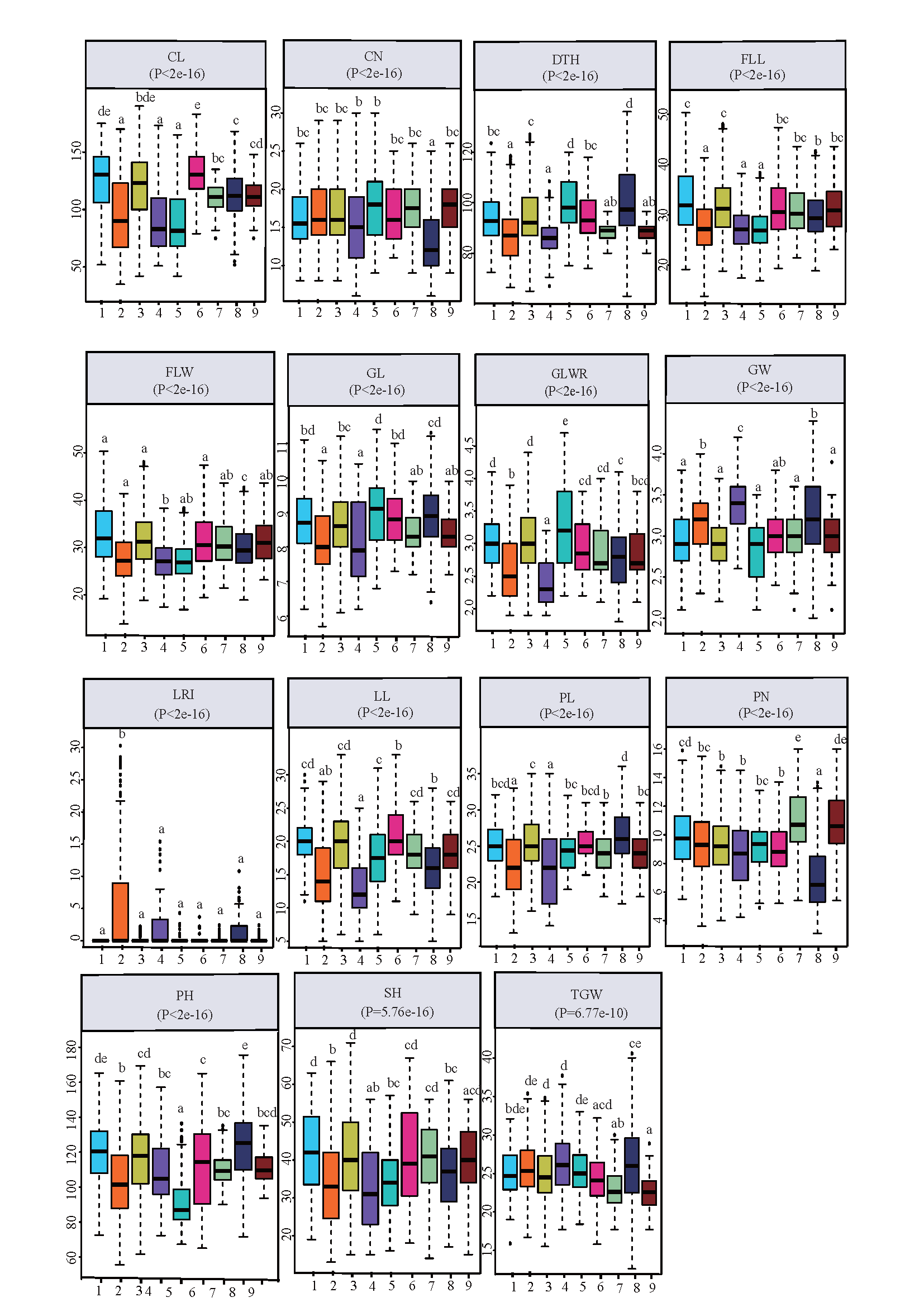

Supplement: Supplementary Figure 1 — Gene structures of knockout mutants of five rice OsGLR genes. (A-E) The genes represented by (A-E) are OsGLR2.2, OsGLR9.8, OsGLR6.8, OsGLR4.1 and OsGLR7.1. [file DataSheet_3.zip › Supplementary Figure 13 glr6_5.tif]

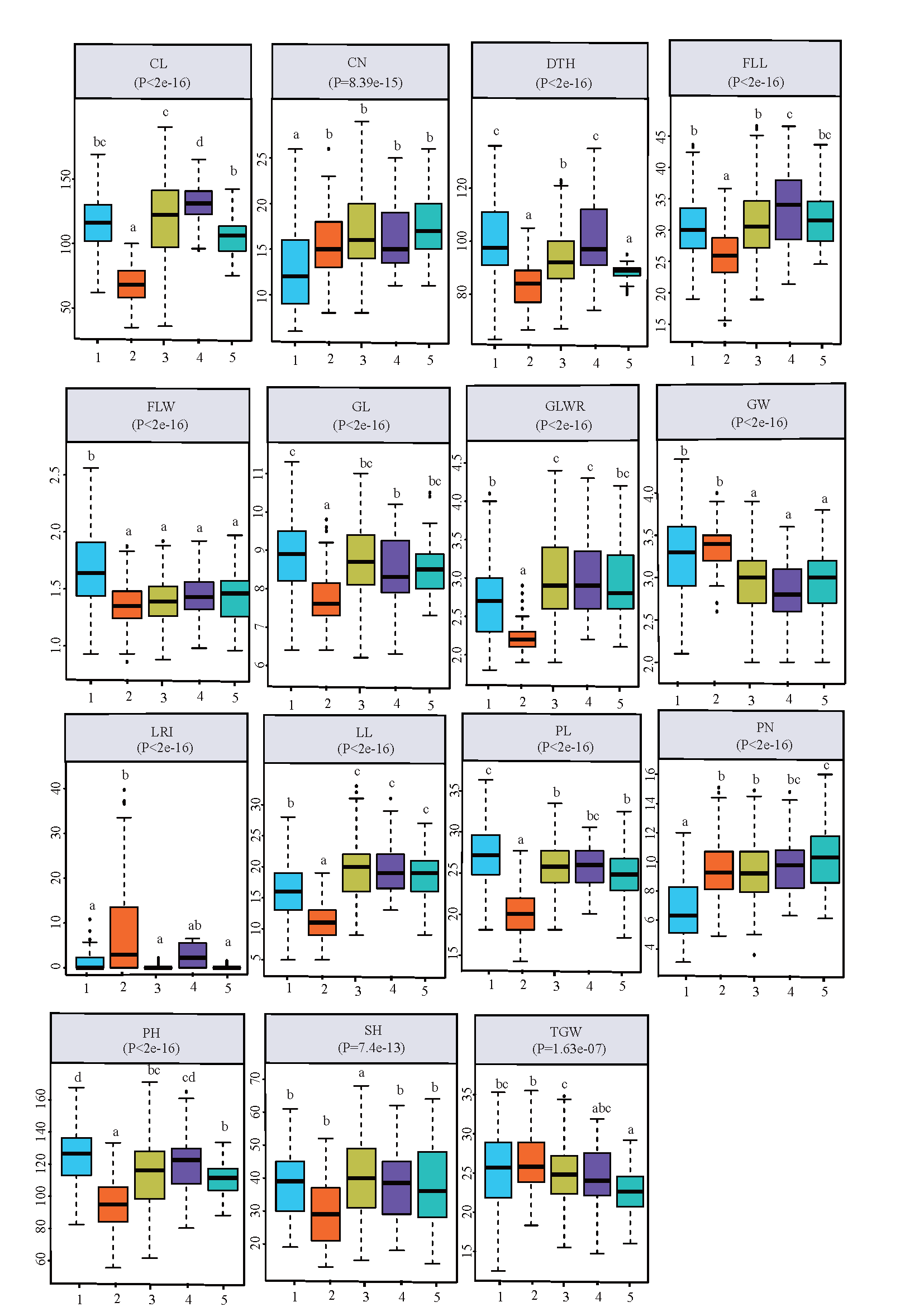

Supplement: Supplementary Figure 1 — Gene structures of knockout mutants of five rice OsGLR genes. (A-E) The genes represented by (A-E) are OsGLR2.2, OsGLR9.8, OsGLR6.8, OsGLR4.1 and OsGLR7.1. [file DataSheet_3.zip › Supplementary Figure 14 glr6_6.tif]

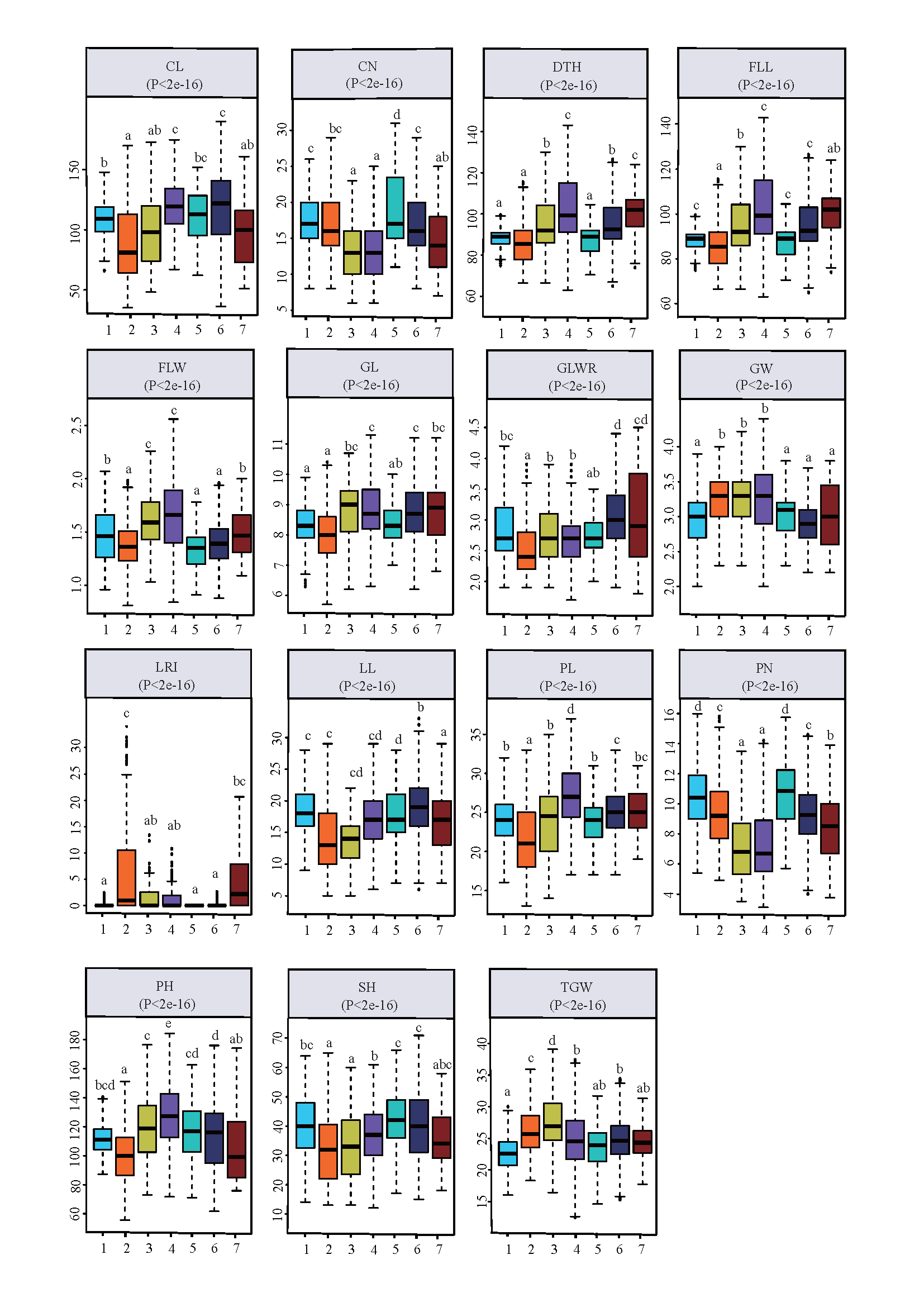

Supplement: Supplementary Figure 1 — Gene structures of knockout mutants of five rice OsGLR genes. (A-E) The genes represented by (A-E) are OsGLR2.2, OsGLR9.8, OsGLR6.8, OsGLR4.1 and OsGLR7.1. [file DataSheet_3.zip › Supplementary Figure 15 glr6_7.tif]

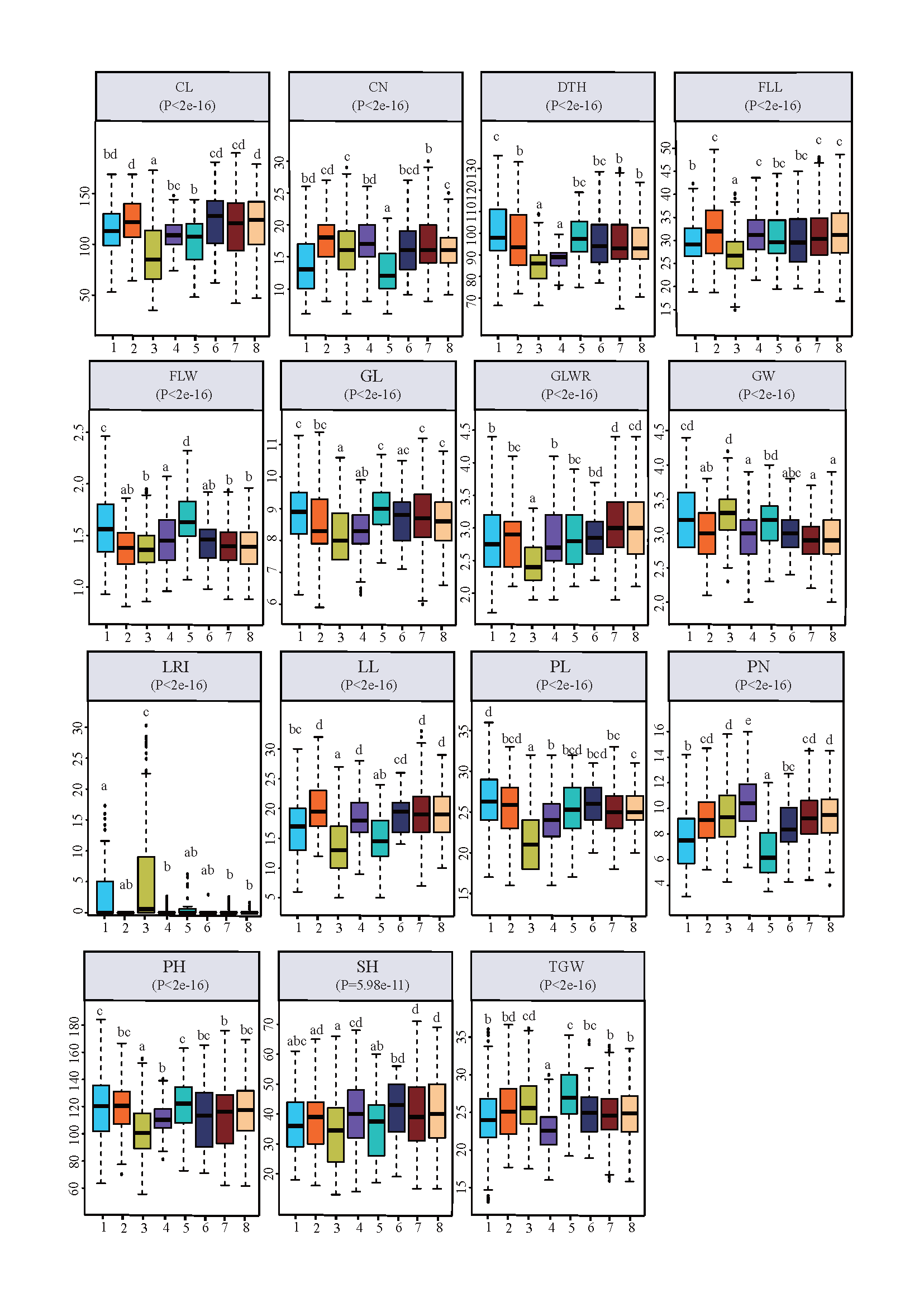

Supplement: Supplementary Figure 1 — Gene structures of knockout mutants of five rice OsGLR genes. (A-E) The genes represented by (A-E) are OsGLR2.2, OsGLR9.8, OsGLR6.8, OsGLR4.1 and OsGLR7.1. [file DataSheet_3.zip › Supplementary Figure 16 glr6_8.tif]

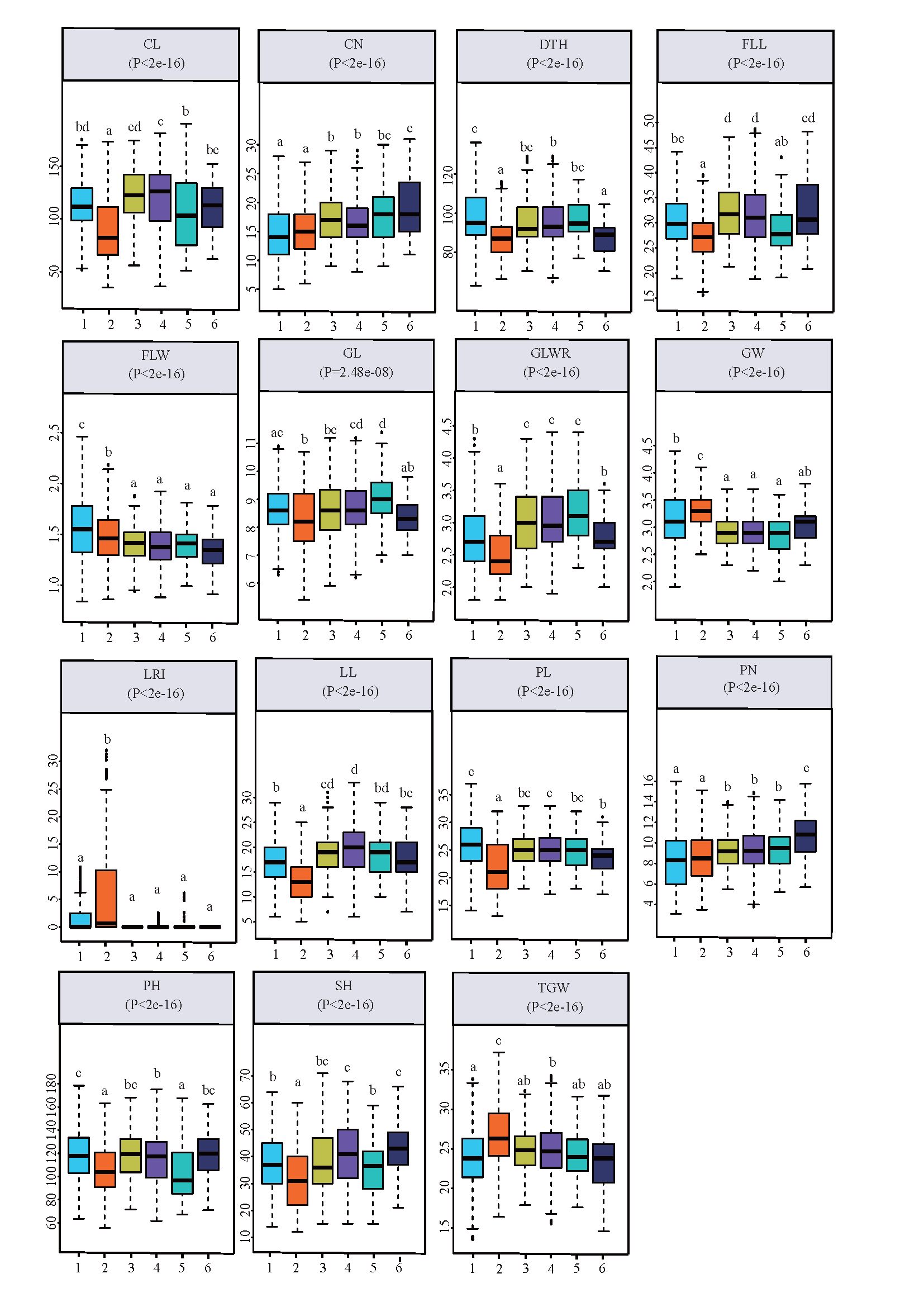

Supplement: Supplementary Figure 1 — Gene structures of knockout mutants of five rice OsGLR genes. (A-E) The genes represented by (A-E) are OsGLR2.2, OsGLR9.8, OsGLR6.8, OsGLR4.1 and OsGLR7.1. [file DataSheet_3.zip › Supplementary Figure 17 glr6_9.tif]

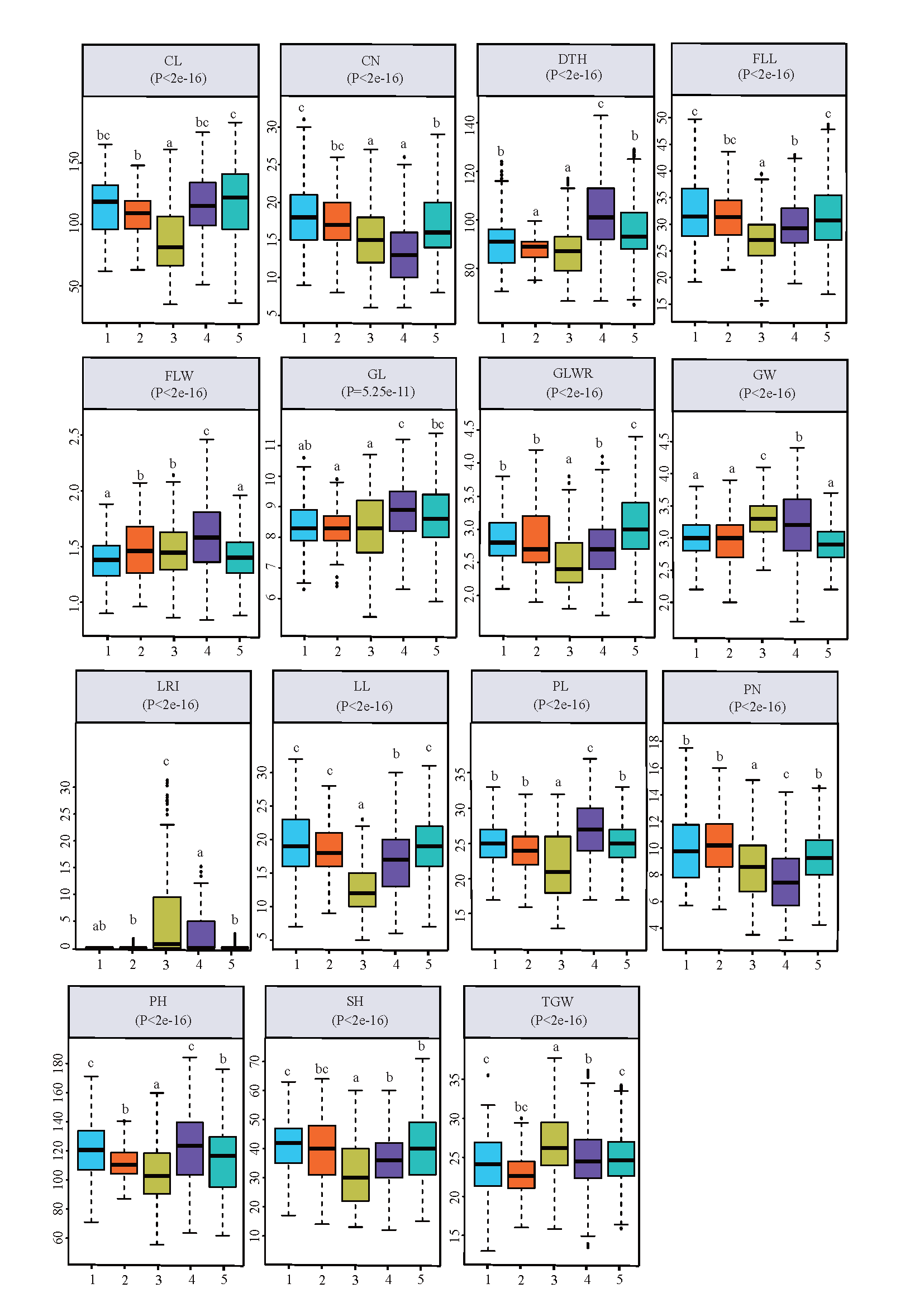

Supplement: Supplementary Figure 1 — Gene structures of knockout mutants of five rice OsGLR genes. (A-E) The genes represented by (A-E) are OsGLR2.2, OsGLR9.8, OsGLR6.8, OsGLR4.1 and OsGLR7.1. [file DataSheet_3.zip › Supplementary Figure 18 glr6_10.tif]

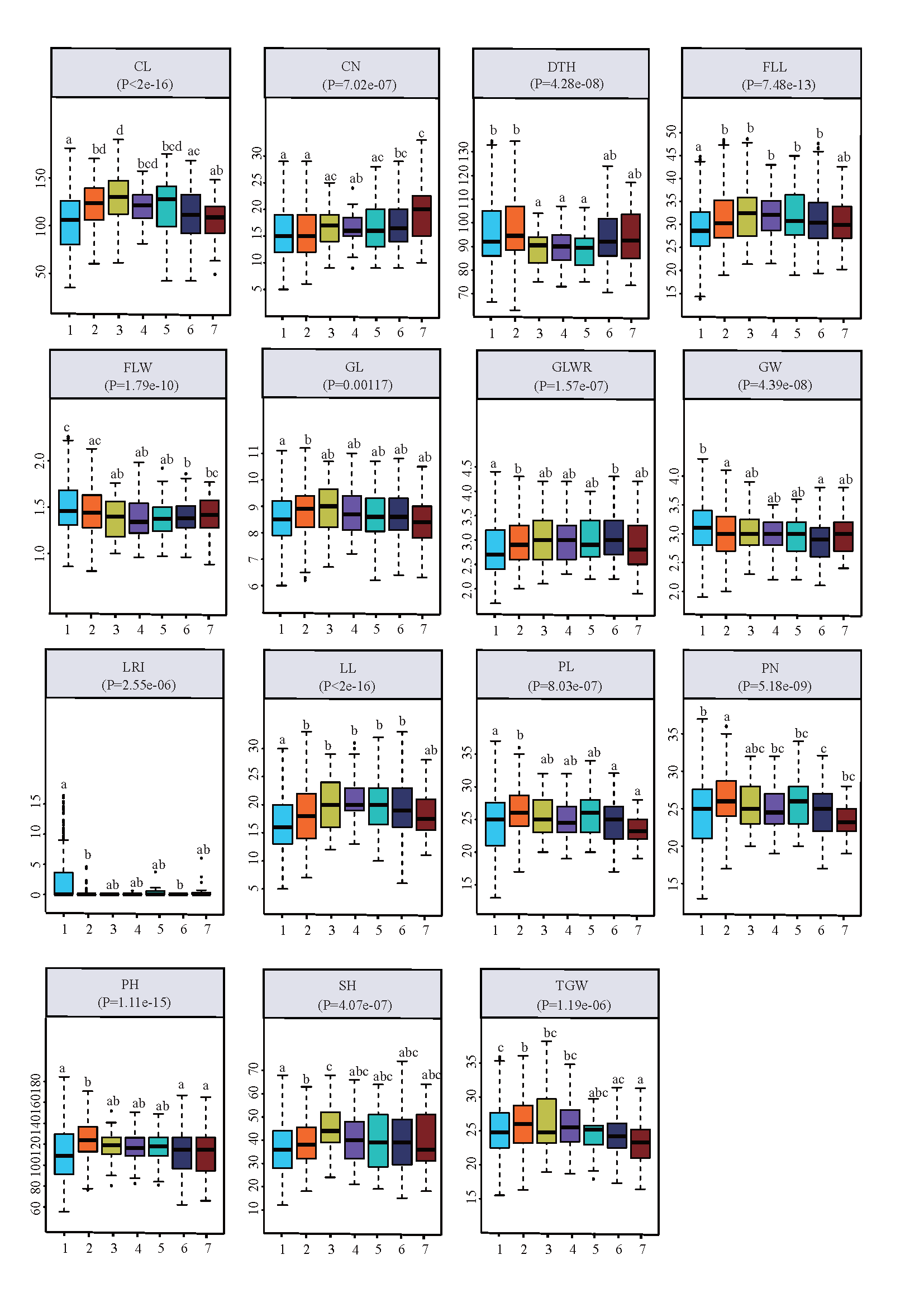

Supplement: Supplementary Figure 1 — Gene structures of knockout mutants of five rice OsGLR genes. (A-E) The genes represented by (A-E) are OsGLR2.2, OsGLR9.8, OsGLR6.8, OsGLR4.1 and OsGLR7.1. [file DataSheet_3.zip › Supplementary Figure 19 glr6_11.tif]

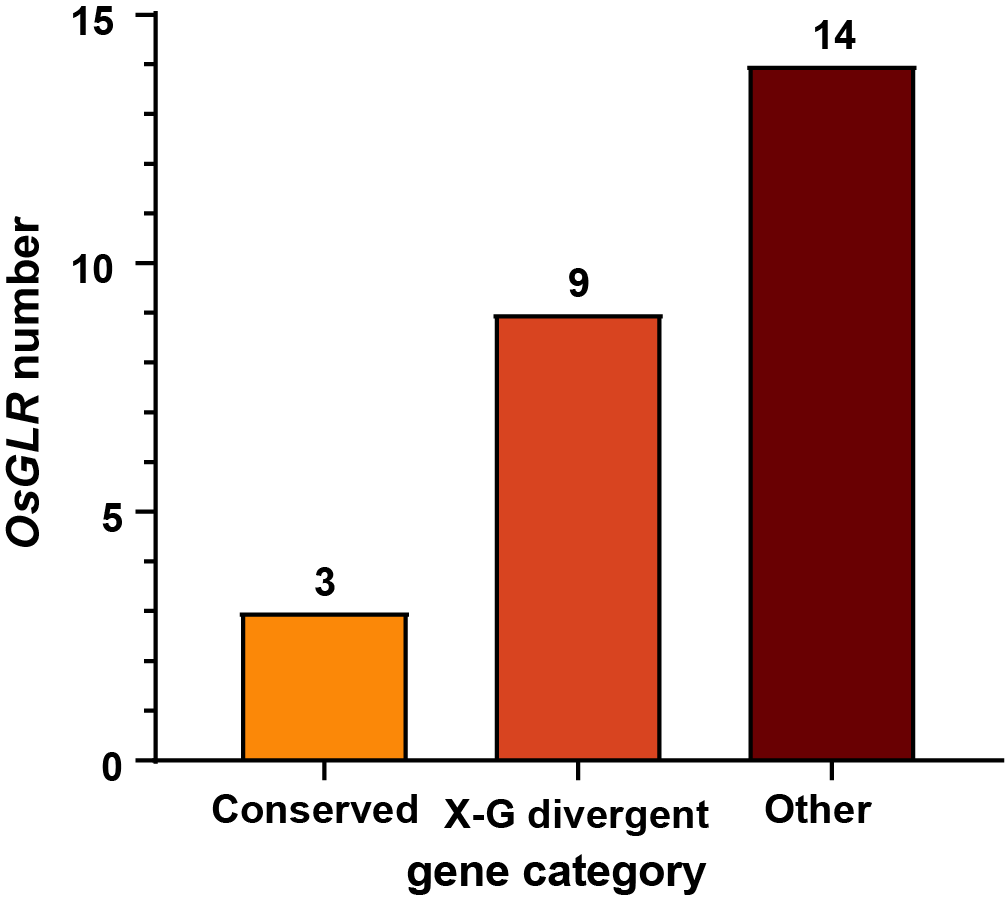

Supplement: Supplementary Figure 1 — Gene structures of knockout mutants of five rice OsGLR genes. (A-E) The genes represented by (A-E) are OsGLR2.2, OsGLR9.8, OsGLR6.8, OsGLR4.1 and OsGLR7.1. [file DataSheet_3.zip › Supplementary Figure 2 Classification of gene categories of rice OsGLR genes .tif]

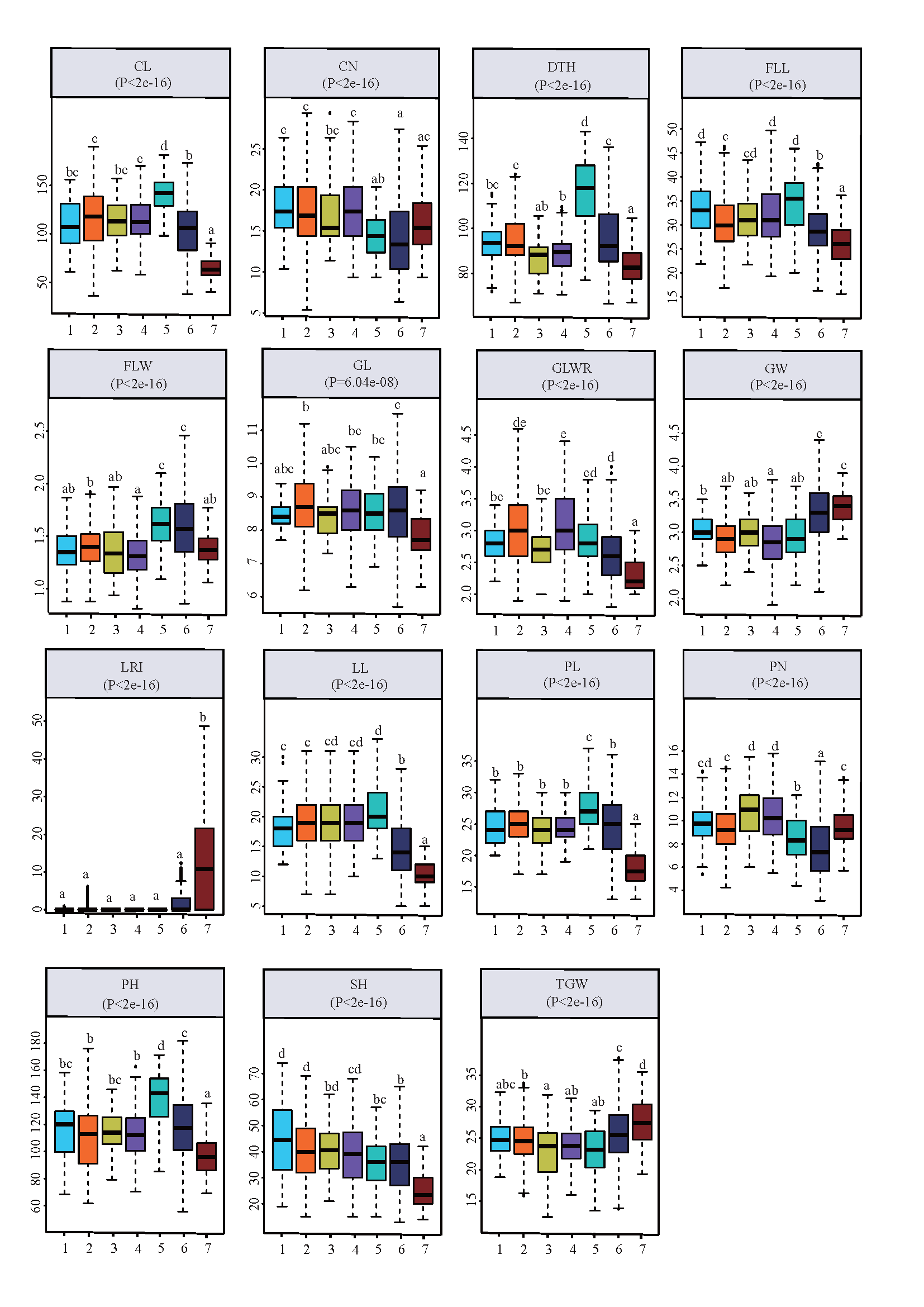

Supplement: Supplementary Figure 1 — Gene structures of knockout mutants of five rice OsGLR genes. (A-E) The genes represented by (A-E) are OsGLR2.2, OsGLR9.8, OsGLR6.8, OsGLR4.1 and OsGLR7.1. [file DataSheet_3.zip › Supplementary Figure 20 glr6_12.tif]

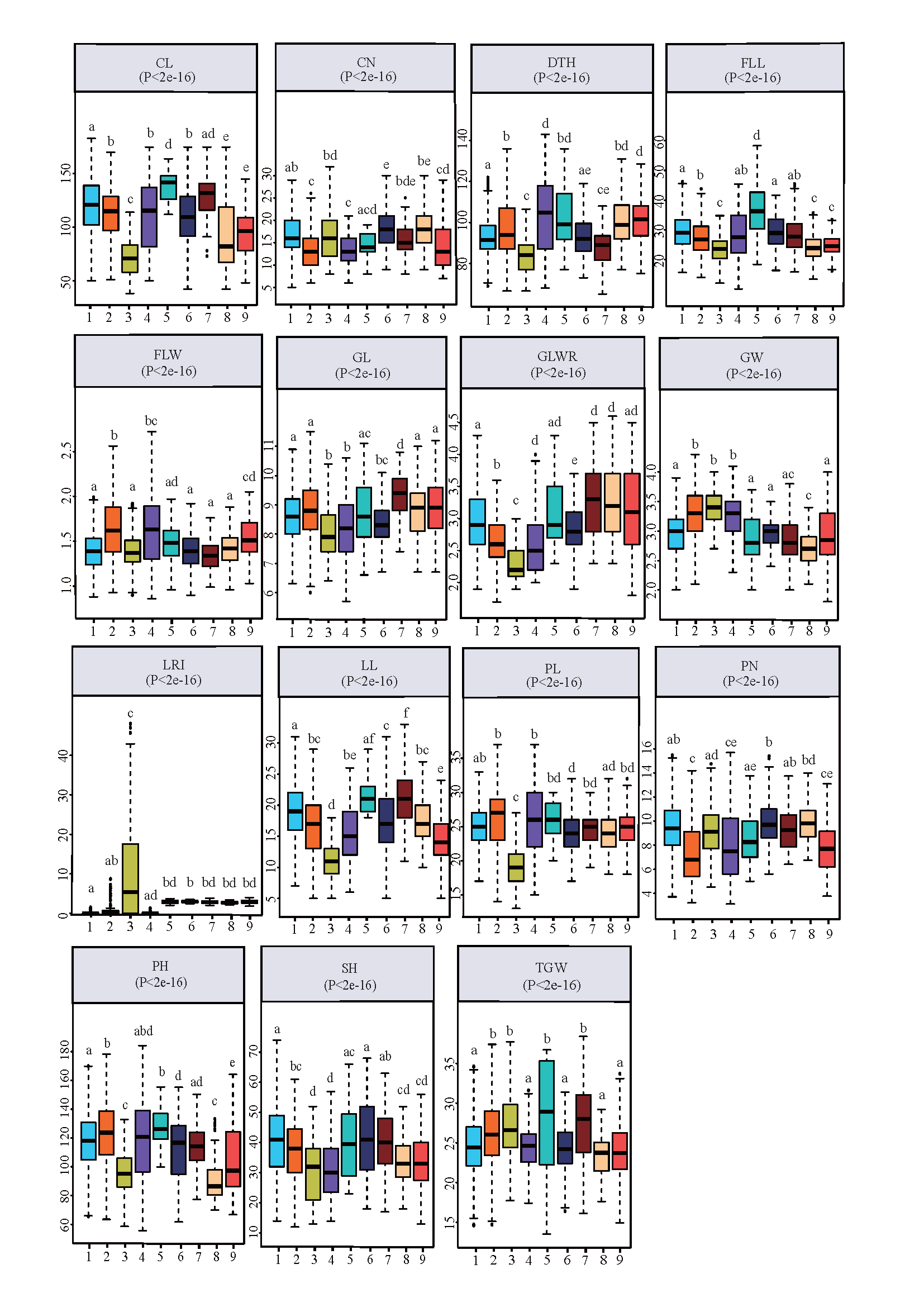

Supplement: Supplementary Figure 1 — Gene structures of knockout mutants of five rice OsGLR genes. (A-E) The genes represented by (A-E) are OsGLR2.2, OsGLR9.8, OsGLR6.8, OsGLR4.1 and OsGLR7.1. [file DataSheet_3.zip › Supplementary Figure 21 glr7_1.tif]

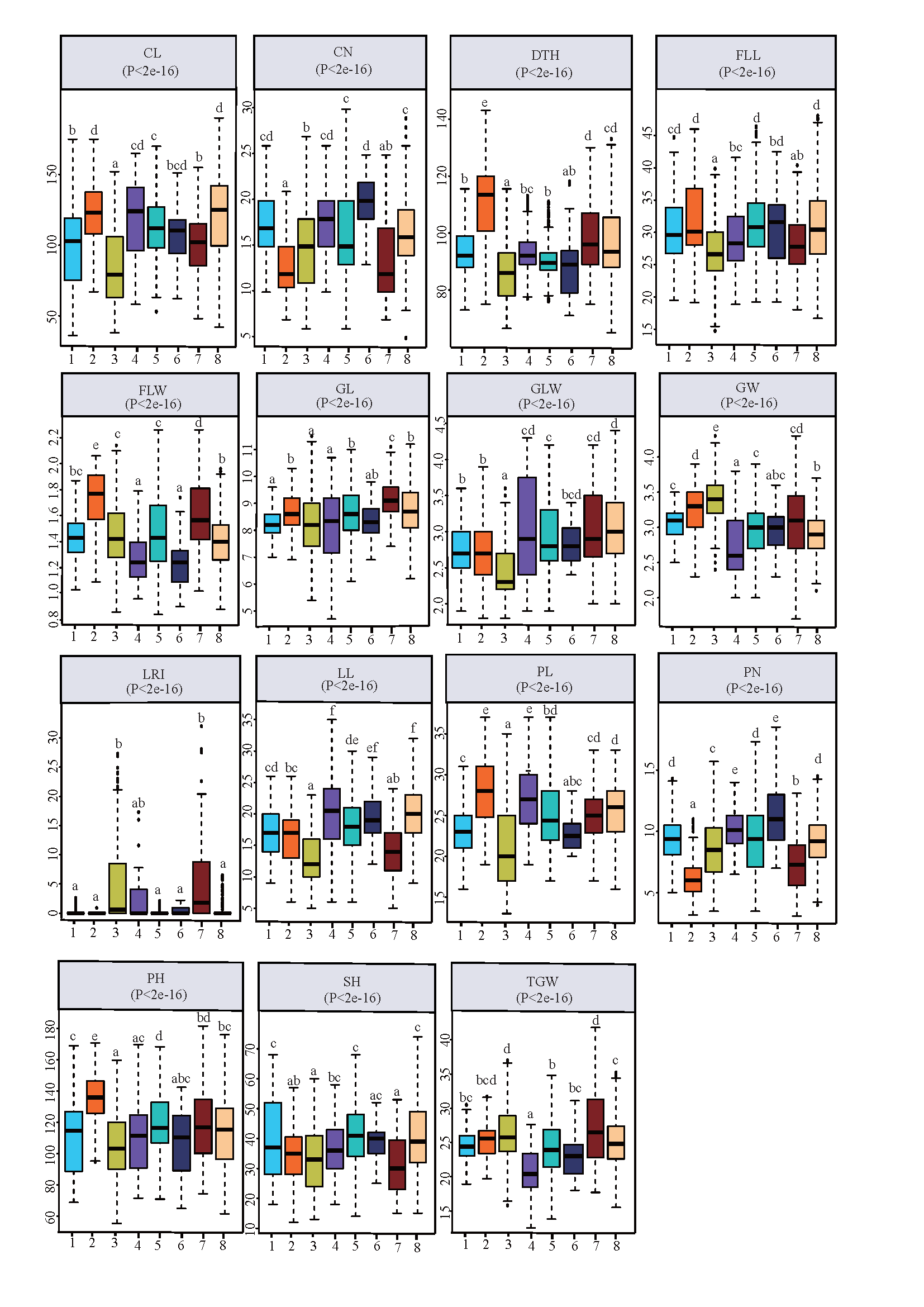

Supplement: Supplementary Figure 1 — Gene structures of knockout mutants of five rice OsGLR genes. (A-E) The genes represented by (A-E) are OsGLR2.2, OsGLR9.8, OsGLR6.8, OsGLR4.1 and OsGLR7.1. [file DataSheet_3.zip › Supplementary Figure 22 glr7_2.tif]

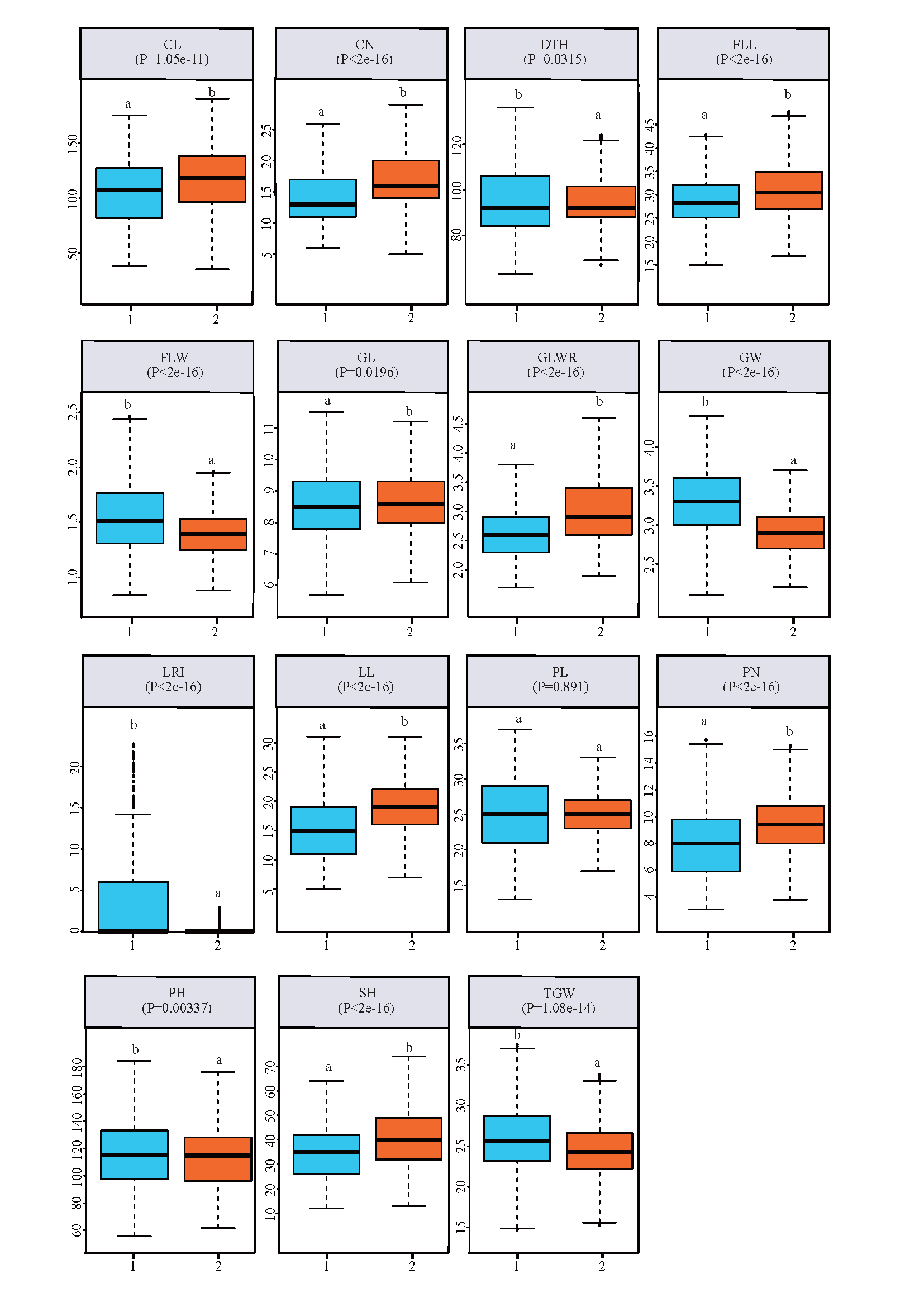

Supplement: Supplementary Figure 1 — Gene structures of knockout mutants of five rice OsGLR genes. (A-E) The genes represented by (A-E) are OsGLR2.2, OsGLR9.8, OsGLR6.8, OsGLR4.1 and OsGLR7.1. [file DataSheet_3.zip › Supplementary Figure 23 glr9_1.tif]

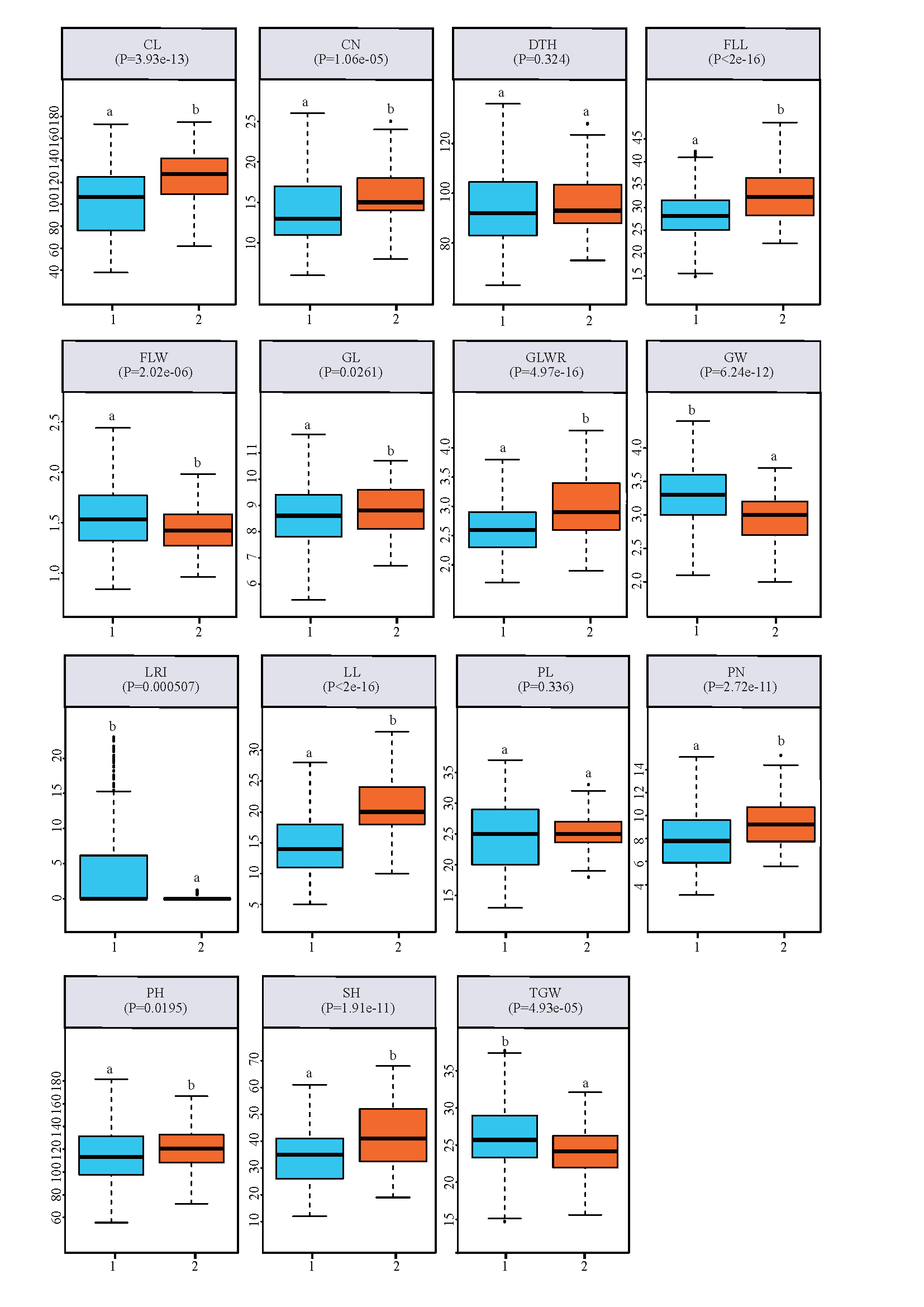

Supplement: Supplementary Figure 1 — Gene structures of knockout mutants of five rice OsGLR genes. (A-E) The genes represented by (A-E) are OsGLR2.2, OsGLR9.8, OsGLR6.8, OsGLR4.1 and OsGLR7.1. [file DataSheet_3.zip › Supplementary Figure 24 glr9_2.tif]

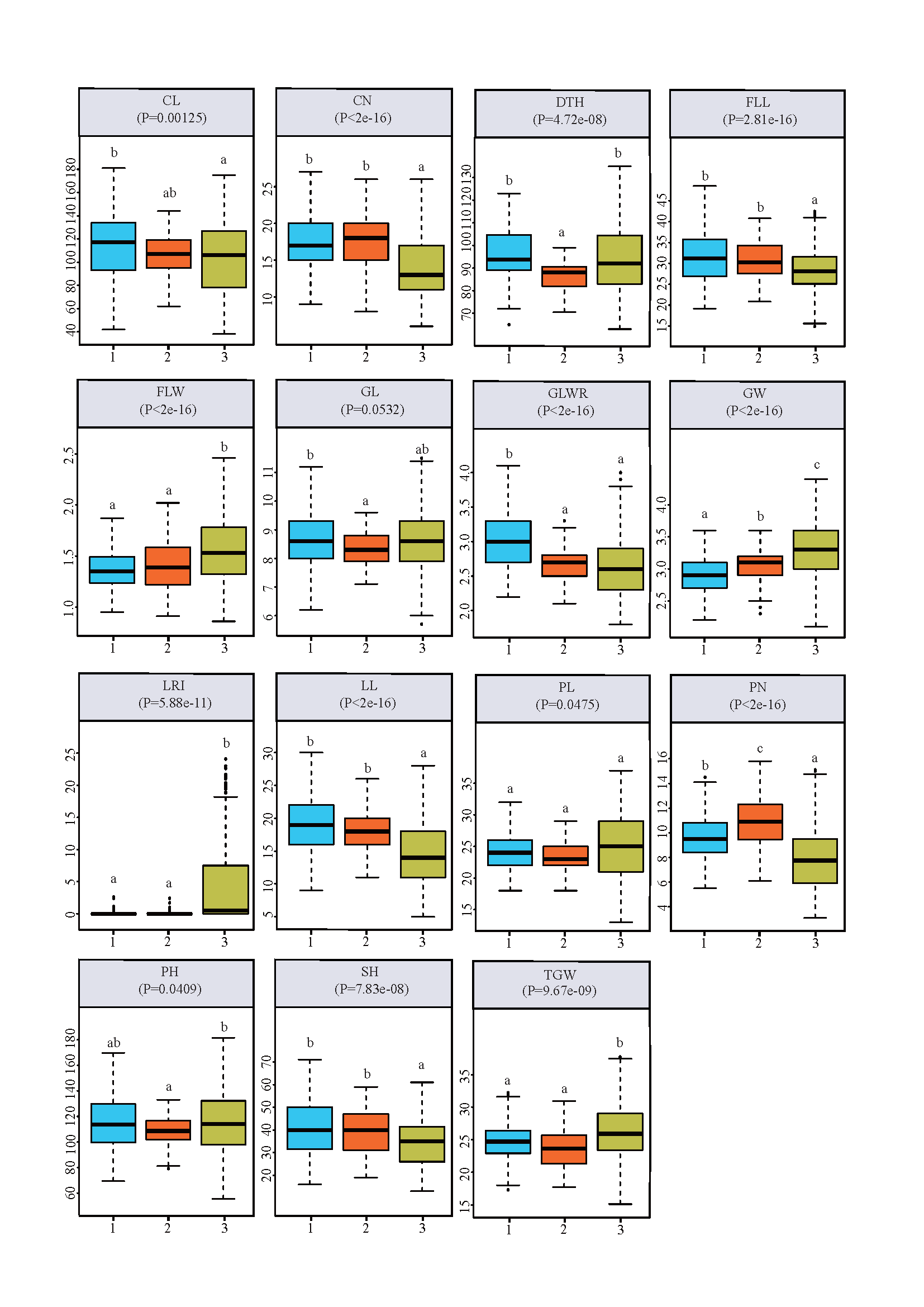

Supplement: Supplementary Figure 1 — Gene structures of knockout mutants of five rice OsGLR genes. (A-E) The genes represented by (A-E) are OsGLR2.2, OsGLR9.8, OsGLR6.8, OsGLR4.1 and OsGLR7.1. [file DataSheet_3.zip › Supplementary Figure 25 glr9_3.tif]

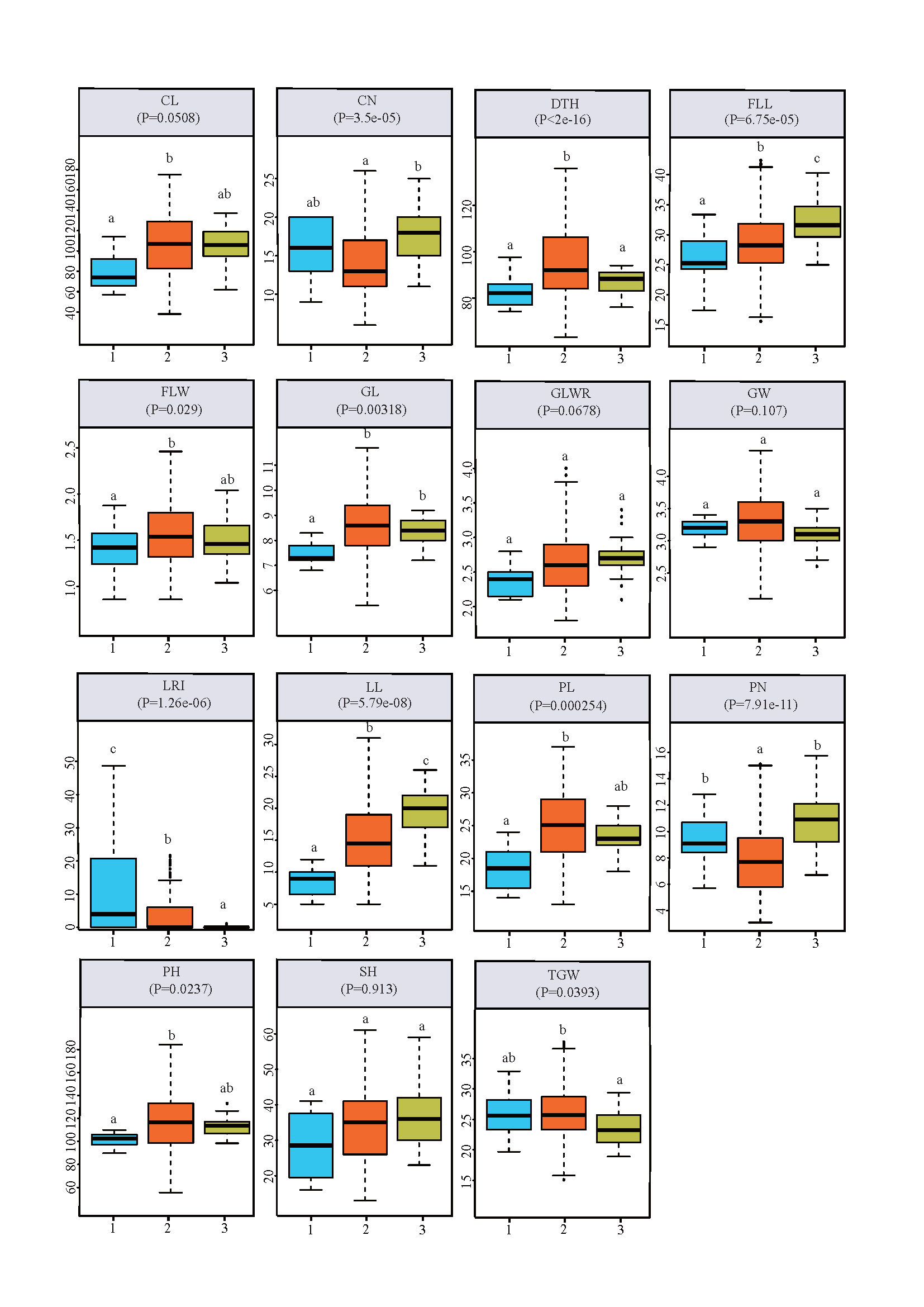

Supplement: Supplementary Figure 1 — Gene structures of knockout mutants of five rice OsGLR genes. (A-E) The genes represented by (A-E) are OsGLR2.2, OsGLR9.8, OsGLR6.8, OsGLR4.1 and OsGLR7.1. [file DataSheet_3.zip › Supplementary Figure 26 glr9_4.tif]

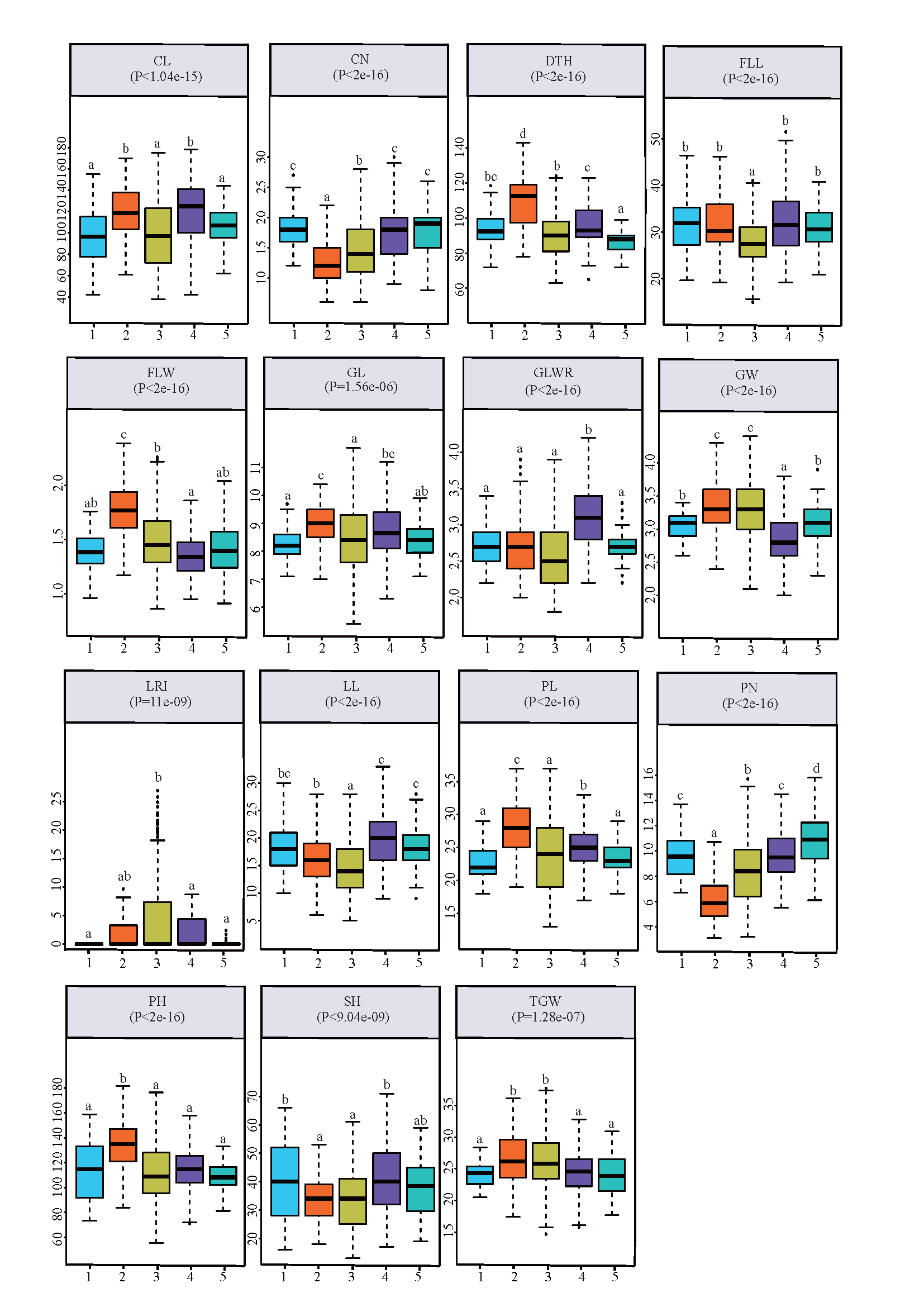

Supplement: Supplementary Figure 1 — Gene structures of knockout mutants of five rice OsGLR genes. (A-E) The genes represented by (A-E) are OsGLR2.2, OsGLR9.8, OsGLR6.8, OsGLR4.1 and OsGLR7.1. [file DataSheet_3.zip › Supplementary Figure 27 glr9_5.tif]

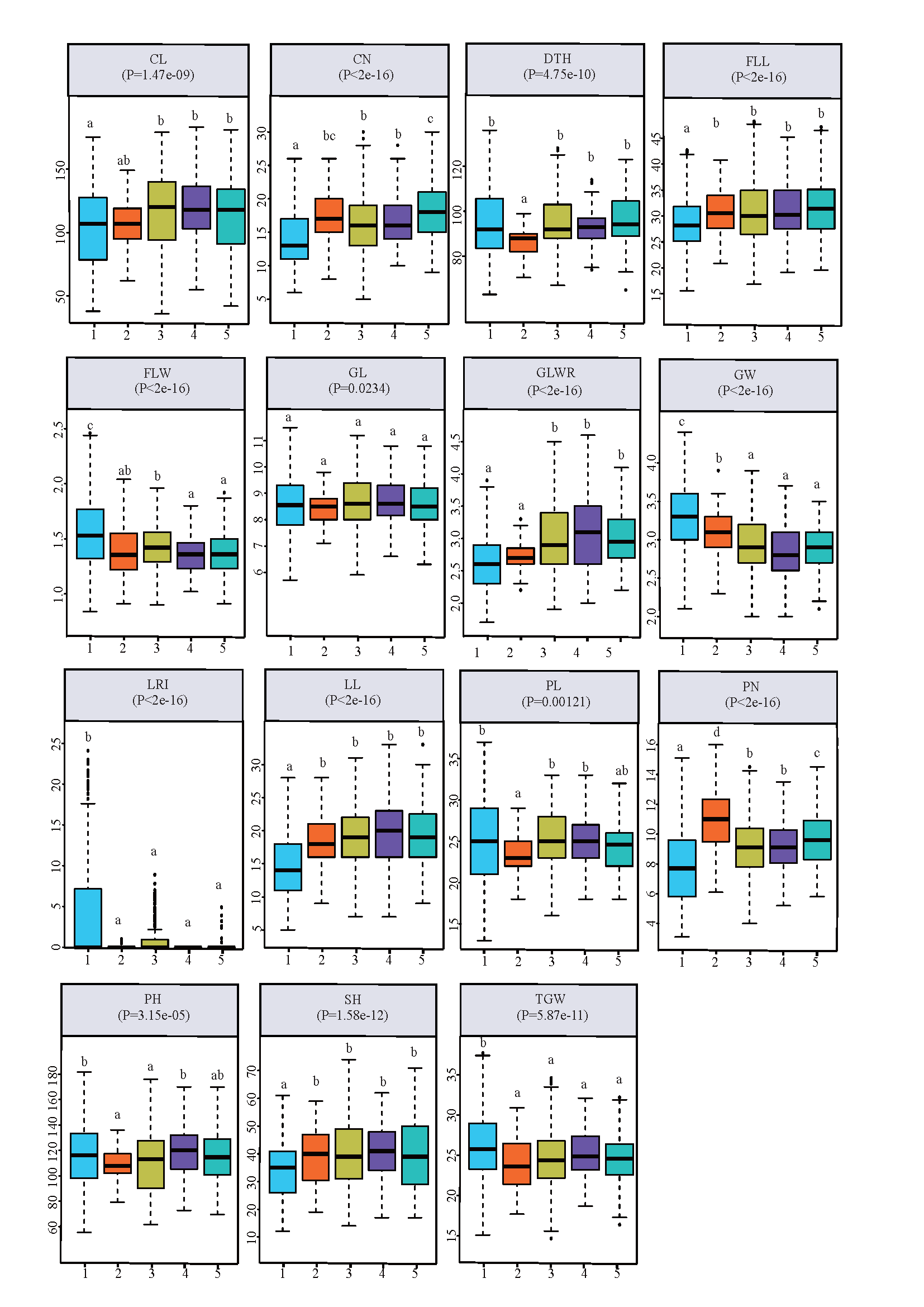

Supplement: Supplementary Figure 1 — Gene structures of knockout mutants of five rice OsGLR genes. (A-E) The genes represented by (A-E) are OsGLR2.2, OsGLR9.8, OsGLR6.8, OsGLR4.1 and OsGLR7.1. [file DataSheet_3.zip › Supplementary Figure 28 glr9_6.tif]

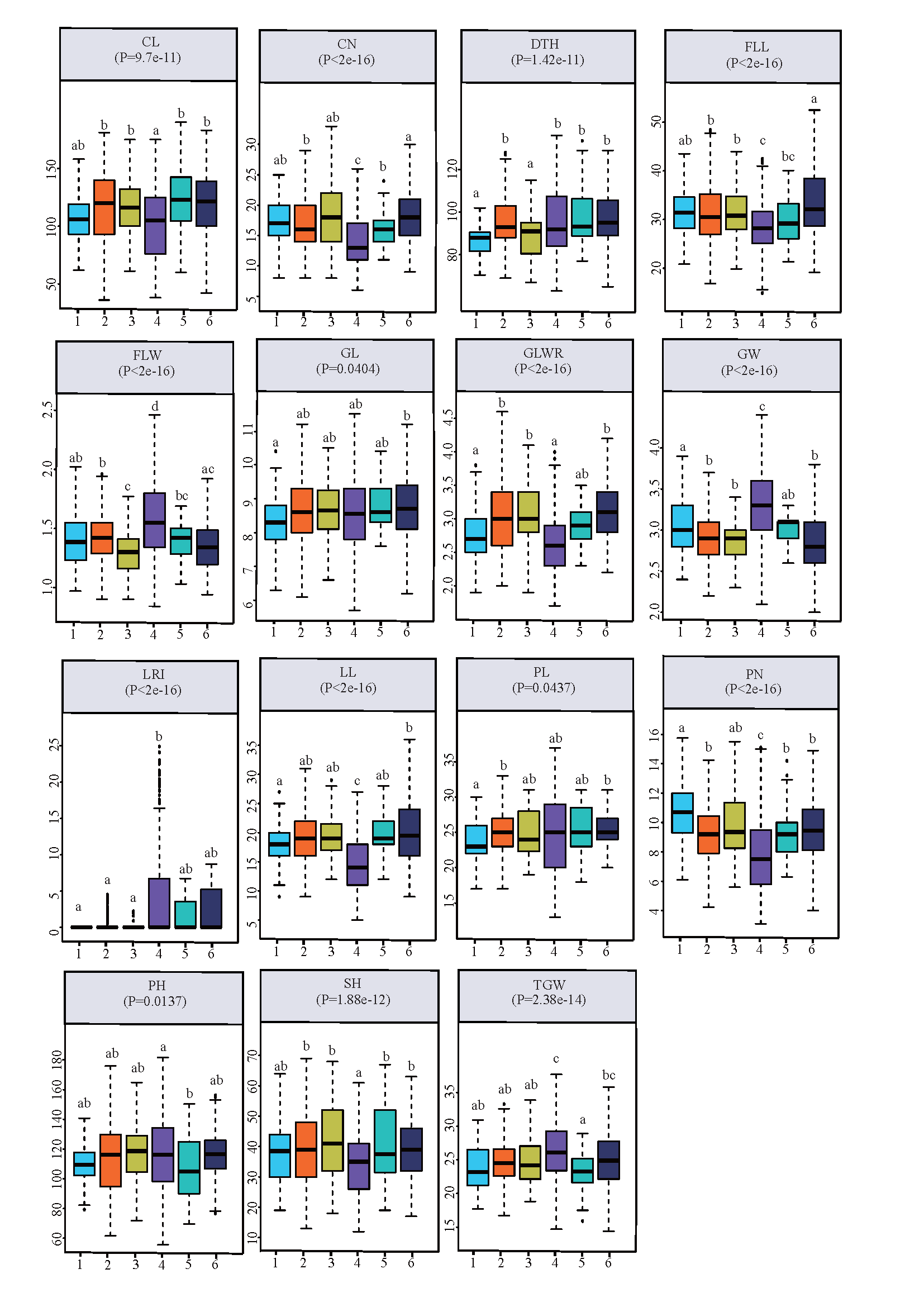

Supplement: Supplementary Figure 1 — Gene structures of knockout mutants of five rice OsGLR genes. (A-E) The genes represented by (A-E) are OsGLR2.2, OsGLR9.8, OsGLR6.8, OsGLR4.1 and OsGLR7.1. [file DataSheet_3.zip › Supplementary Figure 29 glr9_7.tif]

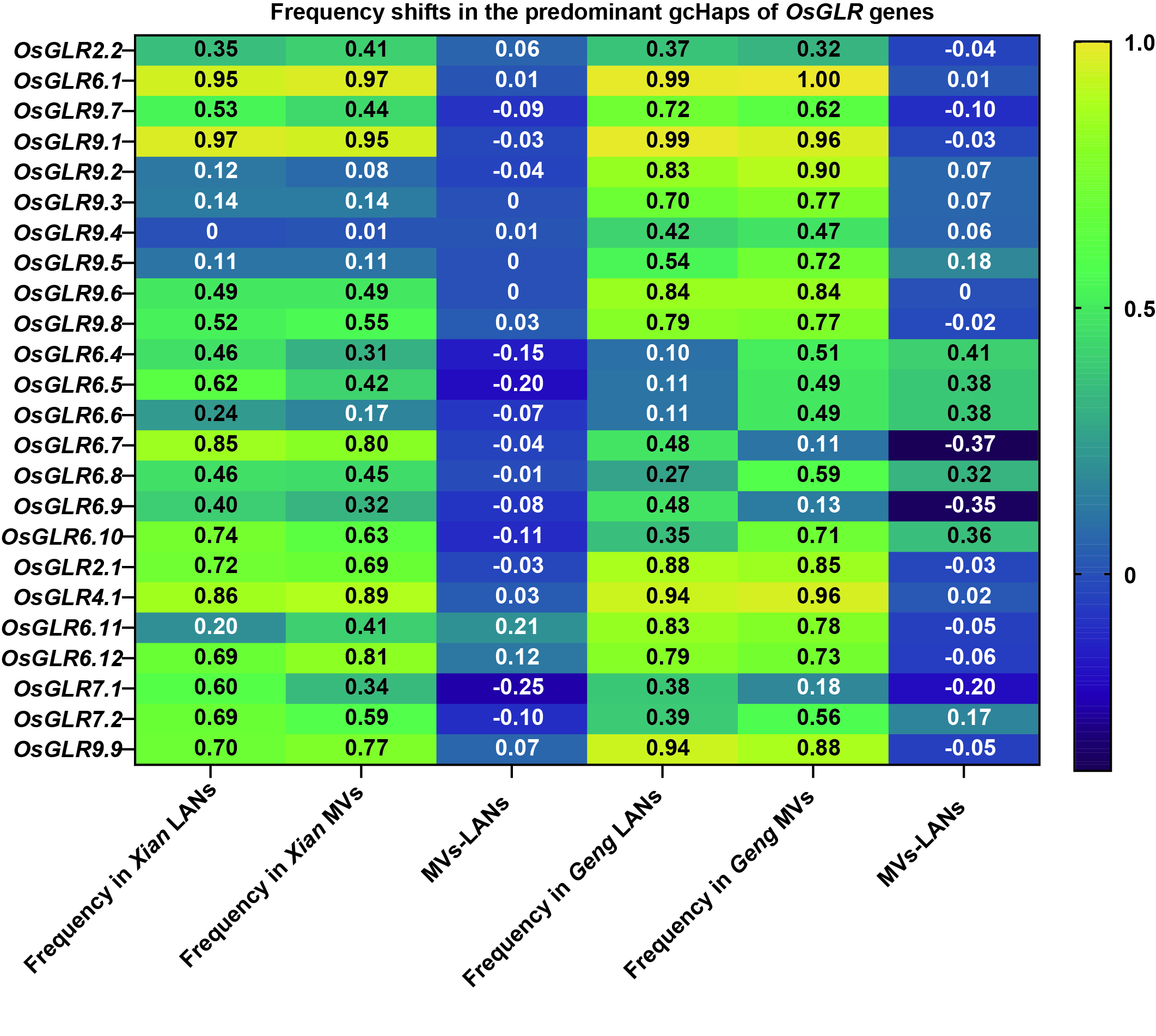

Supplement: Supplementary Figure 1 — Gene structures of knockout mutants of five rice OsGLR genes. (A-E) The genes represented by (A-E) are OsGLR2.2, OsGLR9.8, OsGLR6.8, OsGLR4.1 and OsGLR7.1. [file DataSheet_3.zip › Supplementary Figure 3 Frequency shifts in the predominant gcHaps at 24 GLR genes between the landraces and modern varieties.tif]

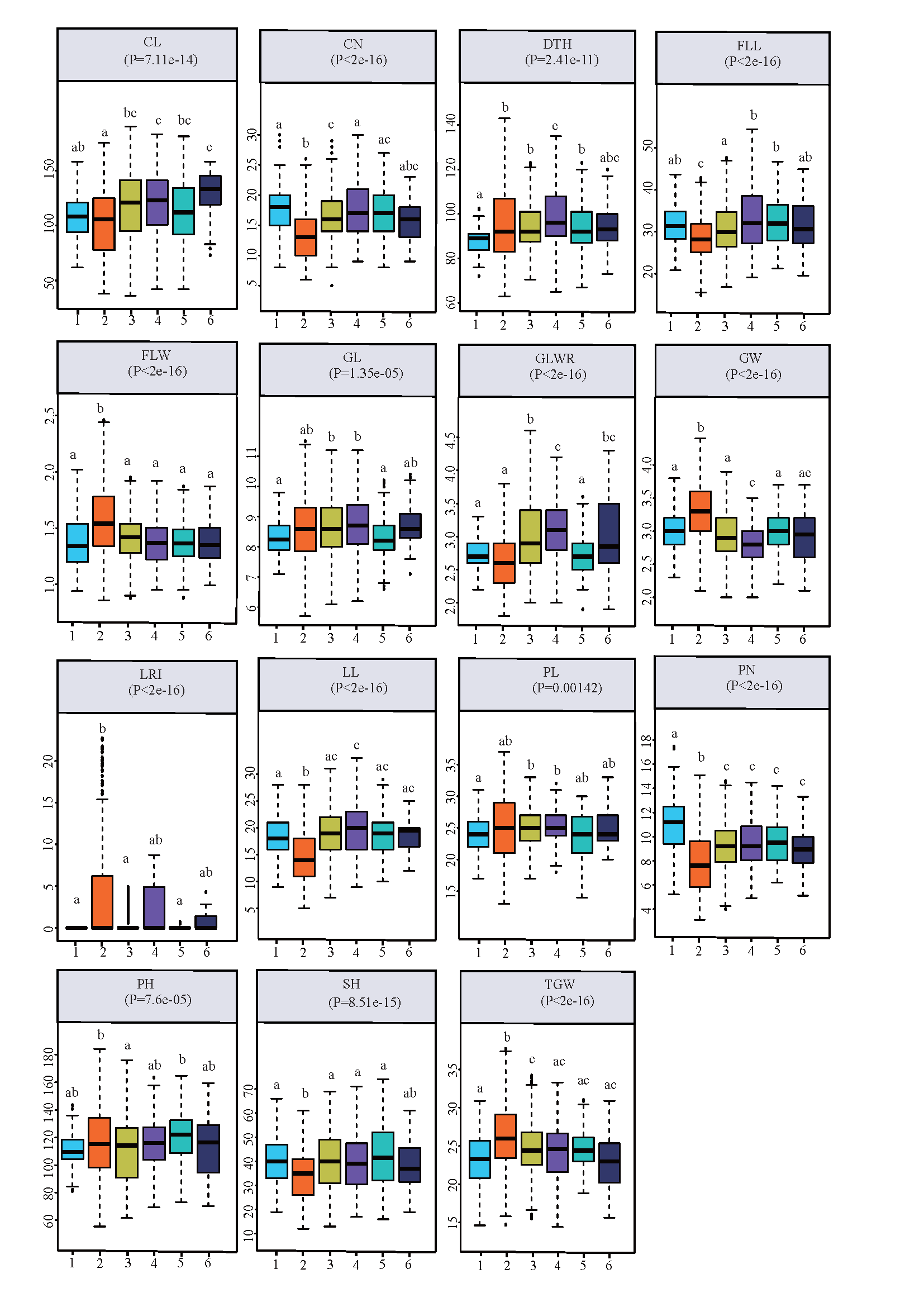

Supplement: Supplementary Figure 1 — Gene structures of knockout mutants of five rice OsGLR genes. (A-E) The genes represented by (A-E) are OsGLR2.2, OsGLR9.8, OsGLR6.8, OsGLR4.1 and OsGLR7.1. [file DataSheet_3.zip › Supplementary Figure 30 glr9_8.tif]

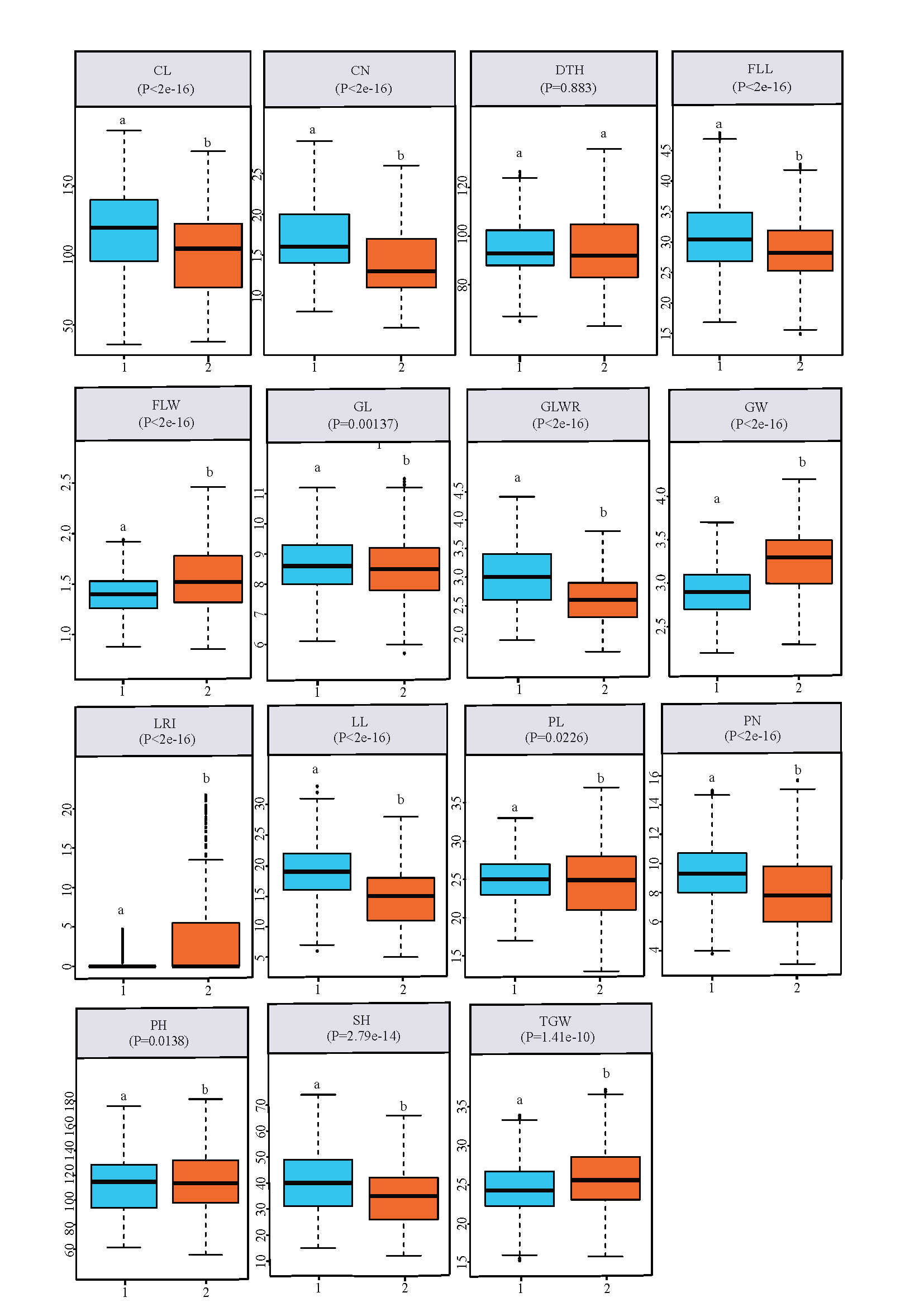

Supplement: Supplementary Figure 1 — Gene structures of knockout mutants of five rice OsGLR genes. (A-E) The genes represented by (A-E) are OsGLR2.2, OsGLR9.8, OsGLR6.8, OsGLR4.1 and OsGLR7.1. [file DataSheet_3.zip › Supplementary Figure 31 glr9_9.tif]

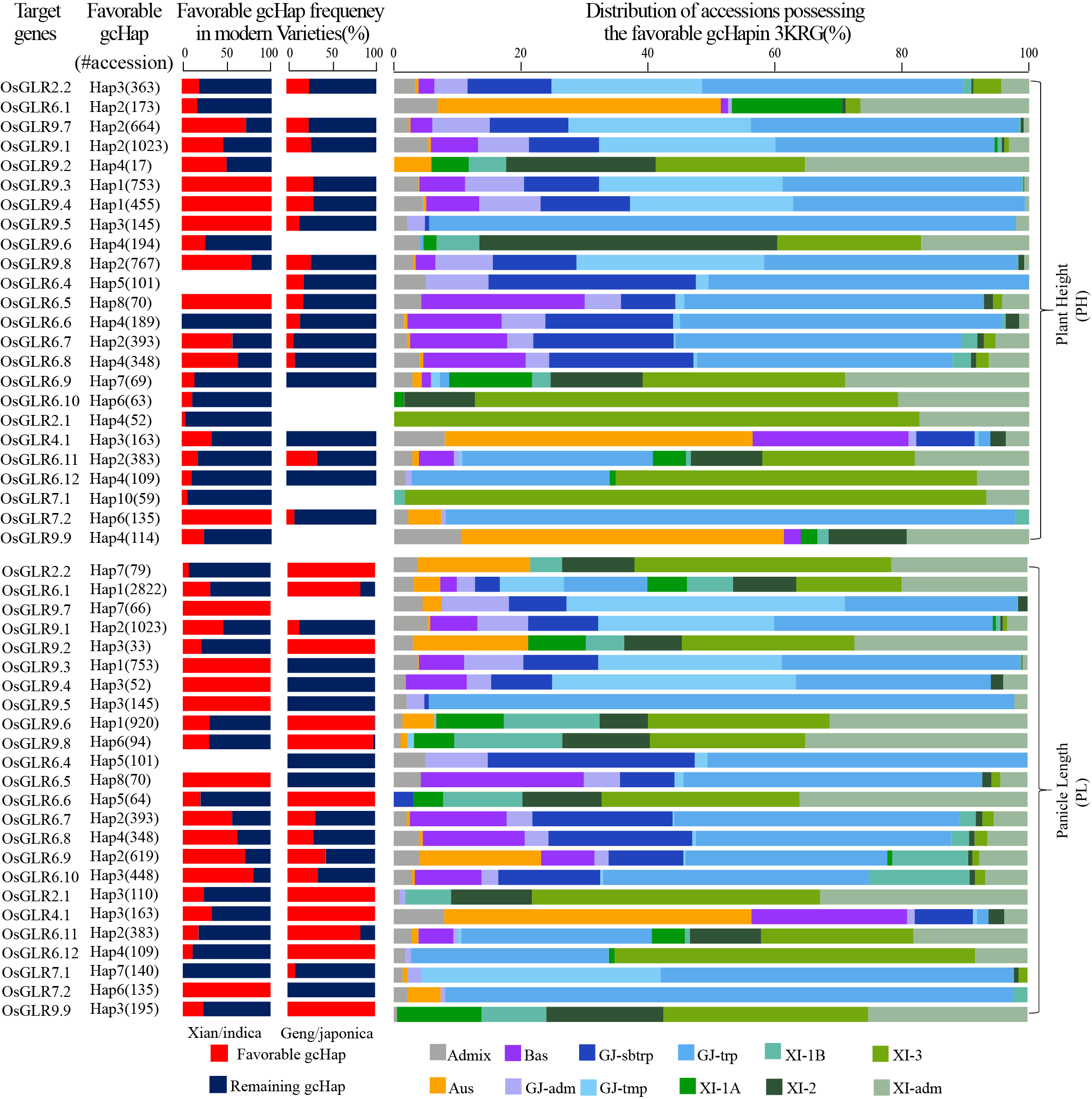

Supplement: Supplementary Figure 1 — Gene structures of knockout mutants of five rice OsGLR genes. (A-E) The genes represented by (A-E) are OsGLR2.2, OsGLR9.8, OsGLR6.8, OsGLR4.1 and OsGLR7.1. [file DataSheet_3.zip › Supplementary Figure 32 Frequencies of the ‘‘favorable’’ gcHaps of all rice OsGLR genes affecting important agronomic traits (PH and PL).tif]

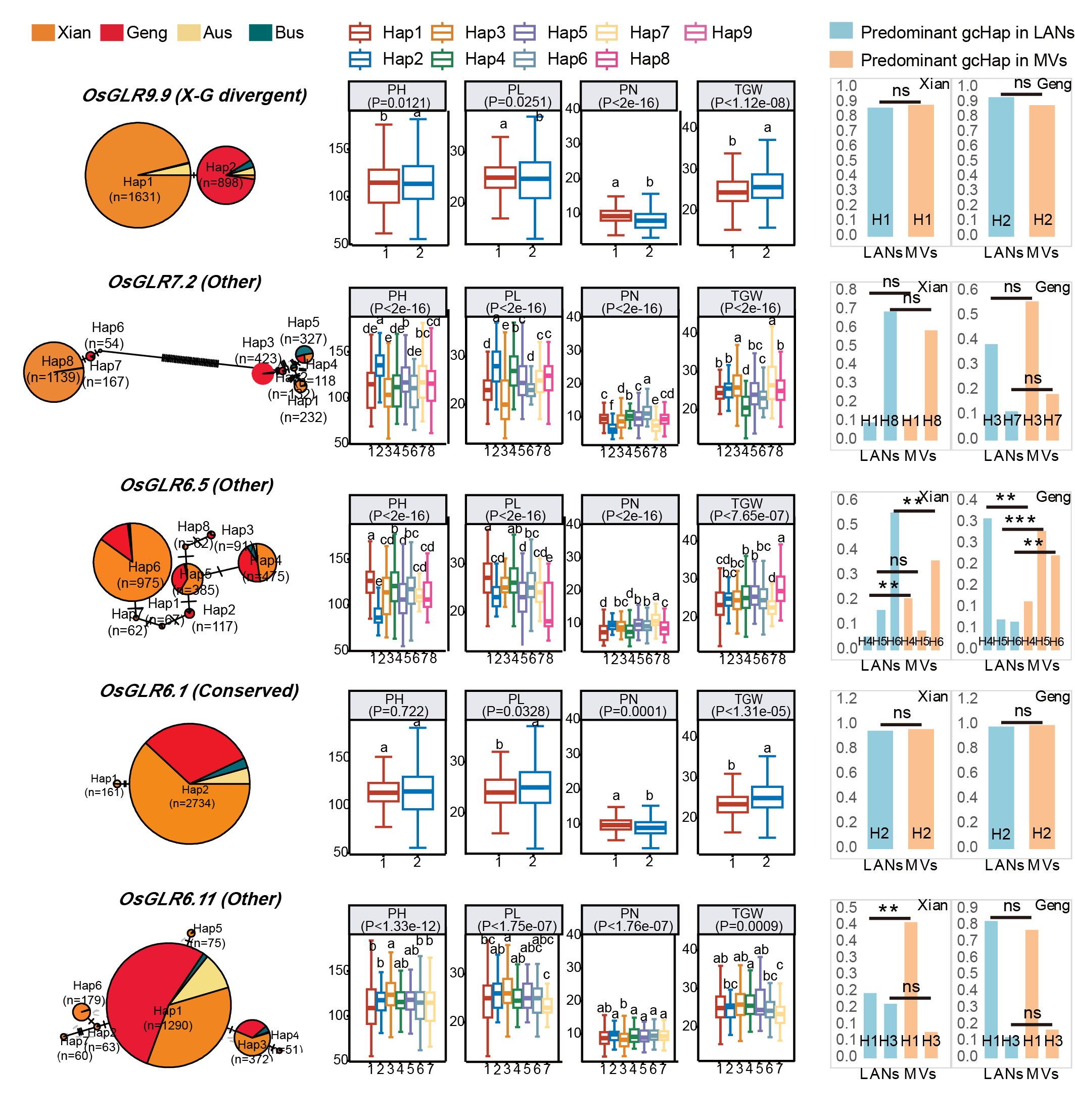

Supplement: Supplementary Figure 1 — Gene structures of knockout mutants of five rice OsGLR genes. (A-E) The genes represented by (A-E) are OsGLR2.2, OsGLR9.8, OsGLR6.8, OsGLR4.1 and OsGLR7.1. [file DataSheet_3.zip › Supplementary Figure 4 Haplotype networks of five overexpressed OsGLR genes.tif]

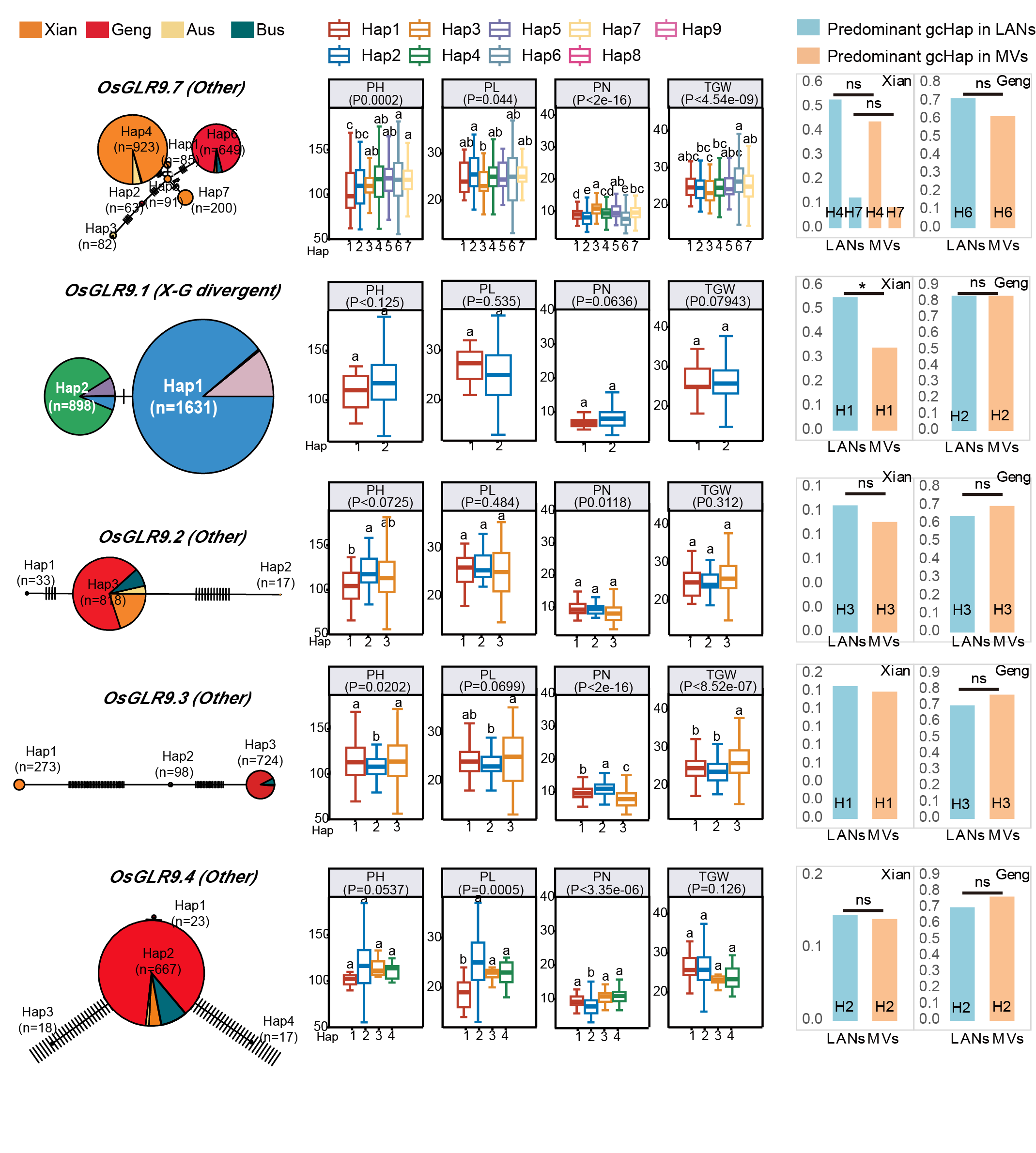

Supplement: Supplementary Figure 1 — Gene structures of knockout mutants of five rice OsGLR genes. (A-E) The genes represented by (A-E) are OsGLR2.2, OsGLR9.8, OsGLR6.8, OsGLR4.1 and OsGLR7.1. [file DataSheet_3.zip › Supplementary Figure 5 Haplotype networks of remaining OsGLR genes.tif]

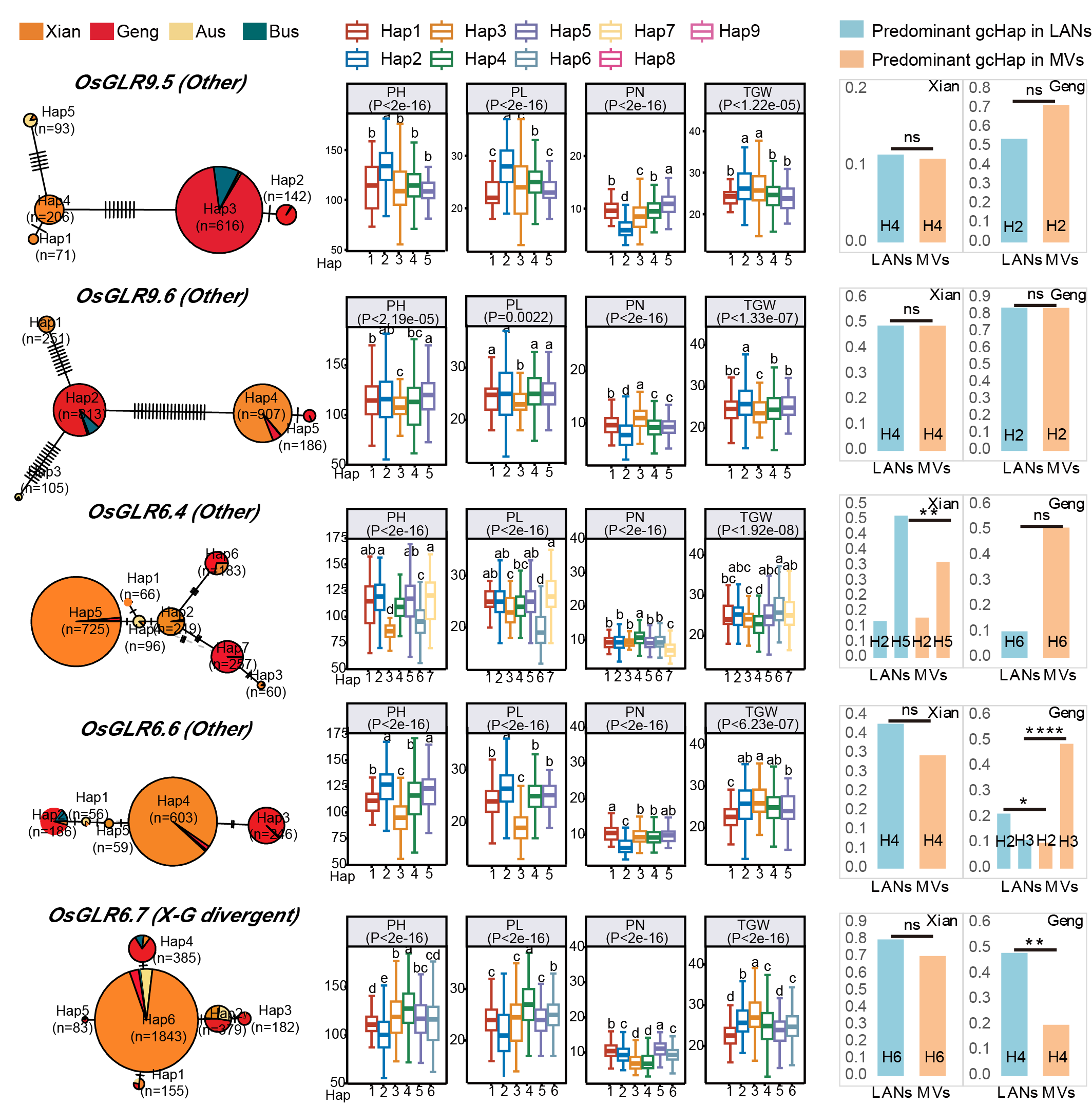

Supplement: Supplementary Figure 1 — Gene structures of knockout mutants of five rice OsGLR genes. (A-E) The genes represented by (A-E) are OsGLR2.2, OsGLR9.8, OsGLR6.8, OsGLR4.1 and OsGLR7.1. [file DataSheet_3.zip › Supplementary Figure 6 Haplotype networks of remaining OsGLR genes.tif]

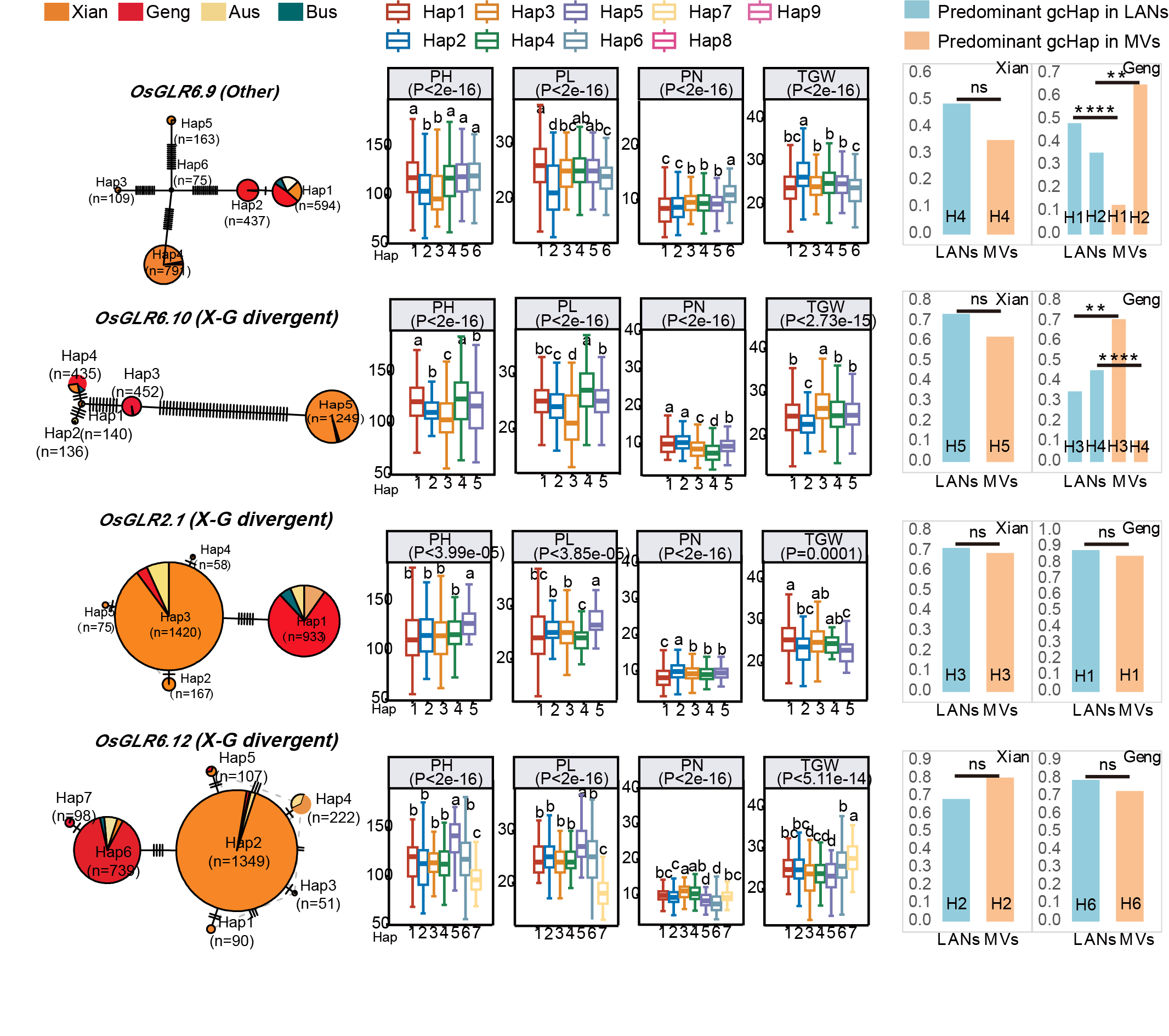

Supplement: Supplementary Figure 1 — Gene structures of knockout mutants of five rice OsGLR genes. (A-E) The genes represented by (A-E) are OsGLR2.2, OsGLR9.8, OsGLR6.8, OsGLR4.1 and OsGLR7.1. [file DataSheet_3.zip › Supplementary Figure 7 Haplotype networks of remaining OsGLR genes.tif]

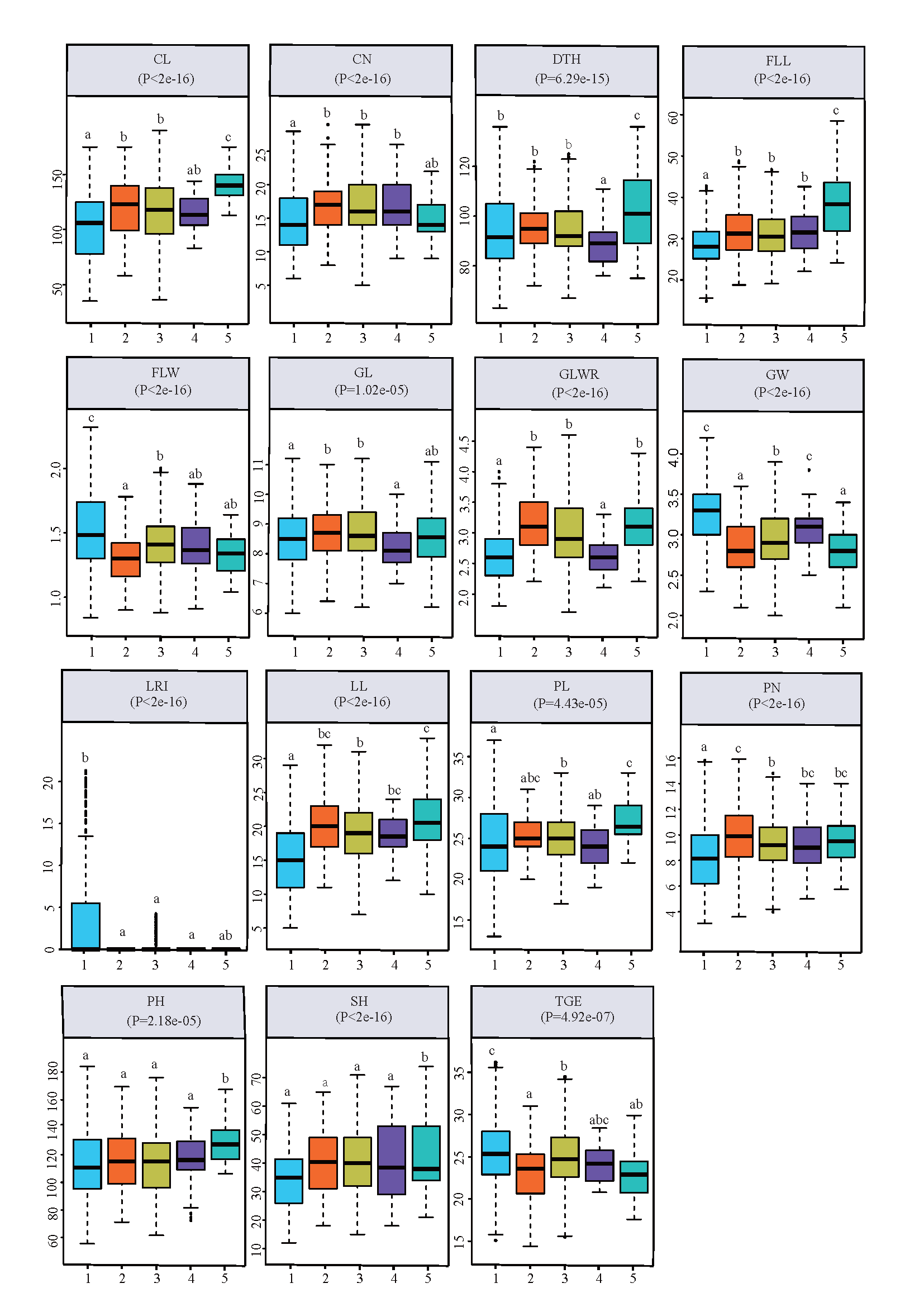

Supplement: Supplementary Figure 1 — Gene structures of knockout mutants of five rice OsGLR genes. (A-E) The genes represented by (A-E) are OsGLR2.2, OsGLR9.8, OsGLR6.8, OsGLR4.1 and OsGLR7.1. [file DataSheet_3.zip › Supplementary Figure 8 osglr2_1.tif]

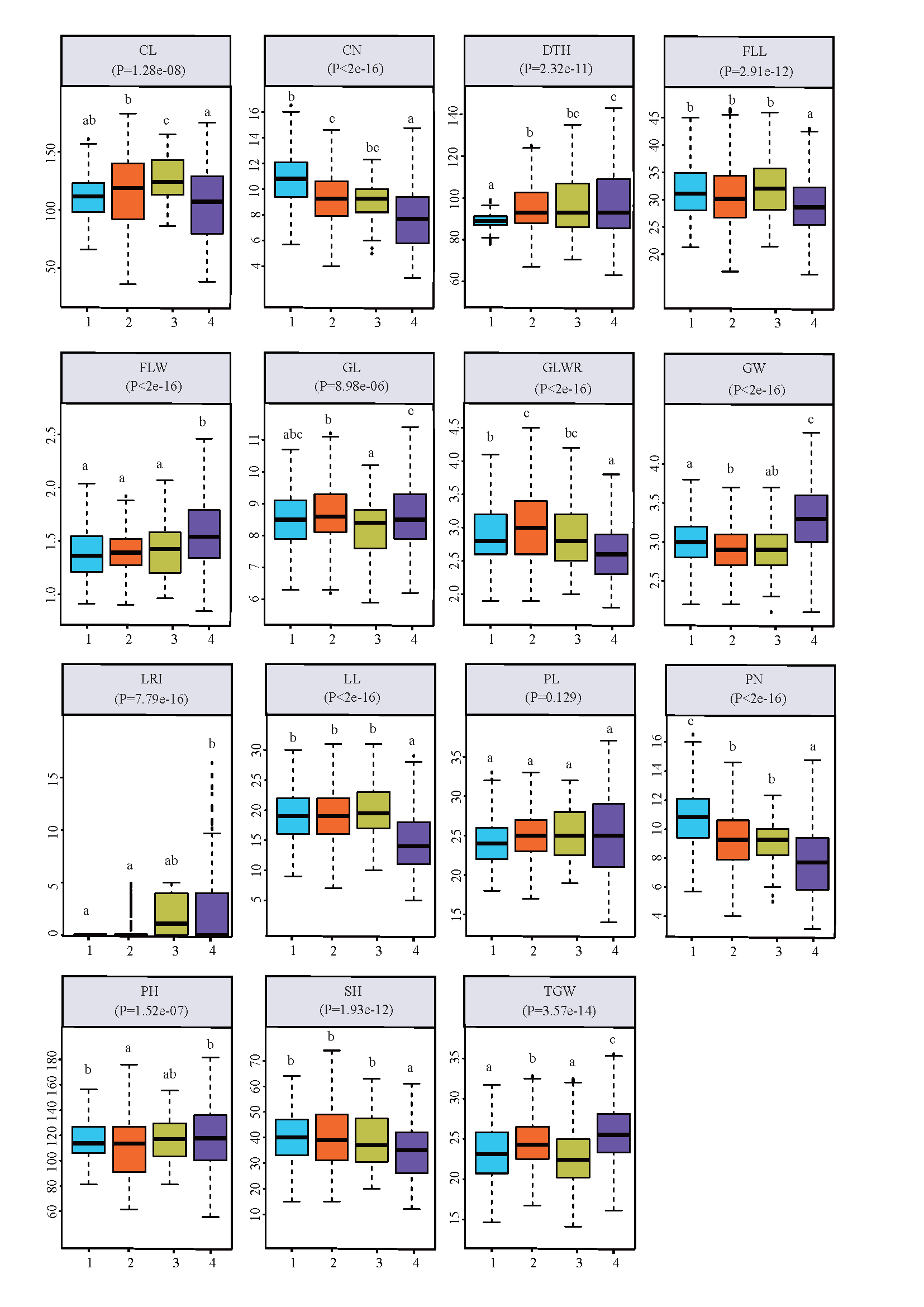

Supplement: Supplementary Figure 1 — Gene structures of knockout mutants of five rice OsGLR genes. (A-E) The genes represented by (A-E) are OsGLR2.2, OsGLR9.8, OsGLR6.8, OsGLR4.1 and OsGLR7.1. [file DataSheet_3.zip › Supplementary Figure 9 osglr2_2.tif]
